# Chemical Exposure: European Citizens’ Perspectives, Trust, and Concerns on Human Biomonitoring Initiatives, Information Needs, and Scientific Results

**DOI:** 10.3390/ijerph18041532

**Published:** 2021-02-05

**Authors:** Maria Uhl, Ricardo R. Santos, Joana Costa, Osvaldo Santos, Ana Virgolino, David S. Evans, Cora Murray, Maurice Mulcahy, Dorothy Ubong, Ovnair Sepai, Joana Lobo Vicente, Michaela Leitner, Silvia Benda-Kahri, Daniela Zanini-Freitag

**Affiliations:** 1 Environment Agency Austria, 1090 Vienna, Austria; michi_leitner@gmx.at (M.L.); silvia.benda-kahri@umweltbundesamt.at (S.B.-K.); 2 Environmental Health Behaviour Lab, Instituto de Saúde Ambiental, Faculdade de Medicina, Universidade de Lisboa, 1649-028 Lisboa, Portugal; ricardoreis@medicina.ulisboa.pt (R.R.S.); jfcosta@medicina.ulisboa.pt (J.C.); osantos@medicina.ulisboa.pt (O.S.); avirgolino@medicina.ulisboa.pt (A.V.); 3 Unbreakable Idea Research, 2550-426 Painho, Portugal; 4 Health Service Executive, Department of Public Health, Merlin Park Regional Hospital, H91N973 Galway, Ireland; david.evans@hse.ie; 5 Health Service Executive, Environmental Health Service, H91EW40 Galway, Ireland; cora.murray@hse.ie (C.M.); maurice.mulcahy@hse.ie (M.M.); 6 Public Health England, London SE1 8UG, UK; dorothy.ubong@phe.gov.uk (D.U.); ovnair.sepai@phe.gov.uk (O.S.); 7 European Environment Agency, 1050 Copenhagen, Denmark; joana.lobo@eea.europa.eu

**Keywords:** human biomonitoring, focus group, citizen reflections, chemical exposure, policy decision making

## Abstract

Over the last few decades, citizen awareness and perception of chemical products has been a topic of interest, particularly concerning national and international policy decision makers, expert/scientific platforms, and the European Union itself. To date, few qualitative studies on human biomonitoring have analysed communication materials, made recommendations in terms of biomonitoring surveillance, or asked for feedback in terms of specific biomonitoring methods. This paper provides in-depth insight on citizens’ perceptions of knowledge of biomonitoring, impact of chemical exposure on daily life, and claims on how results of research should be used. Four semi-structured focus groups were held in Austria, Portugal, Ireland, and the United Kingdom (UK). The cross-sectional observational qualitative design of this study allows for better understanding of public concern regarding chemicals, application, and use of human biomonitoring. The main findings of this study include citizens’ clear articulation on pathways of exposure, the demand on stakeholders for transparent decision-making, and sensitivity in communication of results to the public. Validated and trustful communication is perceived as key to empowering citizens to take action. The results can be used to facilitate decision-making and policy development, and feeds into the awareness needs of similar and future projects in human biomonitoring. Furthermore, it also brings to light ideas and concepts of citizens’ in shaping collaborative knowledge between citizens’, experts, scientists, and policy makers on equal terms.

## 1. Introduction

Citizens are exposed to a wide range of environmental chemicals in their daily lives, in different contexts and via multiple routes, including indoors and outdoors (e.g., air, soil, and water contamination [1,2]; consumer products (e.g., cosmetics, cleaning agents, textiles) [3,4,5]; food, as chemicals are widely used in food production (e.g., antimicrobial drugs in food-producing animals [6], including pesticides to control pests that affect crop production [7], food packaging [8], and pollinators which are directly involved in around one-third of global food production [9]); drinking water [10]; and at work [11,12]. Increased vulnerability to chemical exposure occurs during childhood [13] and pregnancy [14,15], which may impair lifetime health [16].

According to the Special Eurobarometer 456: Chemical Safety (with considerable variation by member states), 65% of citizens from 28 European Union (EU) member states are “a little” or “very much” concerned about being exposed to chemicals in their daily lives, while 45% of the respondents felt well informed about the potential dangers of chemicals included in consumer products. Perceptions regarding the safety of consumer products containing chemicals are divided, with 46% of EU citizens answering “not really” or “not at all”, and 49% tend to agree to some extent with the safety of these products [17].

Public perception of a chemical risk is linked to individual and place attributes; it is not limited to the type and strength of the exposure to a hazardous chemical. Previous studies have shown that chemical cues, for instance, may result in more attributions of symptoms to a chemical cause, even if no exposure occurred in reality [18]. This suggests that people assess risks by different means, according to the probabilistic risk assessments conducted by experts [19]. Different components (deliberative, affective, or experiential [20]), and many factors are involved in the formation of risk perceptions, including literacy and numeracy levels [21], dispositional situations [22], personal experiences, information availability and presentation [23], and physical and psychological well-being of the exposed individual [24]. Health risk perception of chemical exposure has been assessed in numerous settings and targeted populations (e.g., drinking water quality [25], chemical household products [24,26], food additives and contaminants [27], research laboratory workers [28], or recreational beach use by children [29]), as a starting point, to build-up effective risk communication strategies and public health interventions, which ultimately may lead to risk perception and health behaviour changes [20].

The inclusion of citizen perspectives and perceptions is part of a systematic, transparent, and participatory strategy within the Human Biomonitoring Initiative for Europe [30] (HBM4EU). The HBM4EU is a programme involving 30 European countries aimed at coordinating and advancing human biomonitoring in Europe while providing evidence that supports policymaking. A wide range of stakeholders, including governmental bodies, EU agencies, non-governmental organizations, and industry and trade unions participate in the initiative.

One main concern within the HBM4EU consortium is to involve all actors in chemical safety, including the citizens, to (i) identify societal challenges and needs; (ii) set research priorities that address those needs; (iii) ensure that HBM4EU activities are legitimate and credible; (iv) implement procedures that are transparent and accountable; (v) generate benefits for the society; and (vi) disseminate HBM4EU results. Strategies engaging those actors prove to enable a more effective risk governance, a greater acceptance by the public towards human biomonitoring, and, at the same time, bring citizen perspectives into public health policy developments [31,32]. This encourages continuous (health) literacy actions (providing fact sheets, tailor made information) targeted to citizens with different backgrounds in an inclusive, easily accessible approach.

In line with efforts to engage citizens in the initiative, surveys of European citizens were conducted within the HBM4EU, to inform the prioritization process (i.e., identification of relevant substances and their health effects following chemical exposure should be addressed), which serves as a basis for the work undertaken under the project. Despite this initial effort to gather some input from non-specialists in chemical exposure, numerous questions regarding European citizens’ perceptions, concerns and beliefs on chemical exposure and safety, as well as on HBM (i.e., HBM literacy) remain unexplored. Evidence from related fields demonstrate that citizens’ engagement in sensitive questions that directly or indirectly affect them is crucial to support decision making and to avoid distrust in the authorities [33,34]. Considering this, this paper describes the results of four focus groups organized within the HBM4EU framework in four European countries, Austria, Portugal, Ireland, and the United Kingdom (UK). The objectives of this study were to gather further understanding on (i) citizens’ perception of chemical exposure in their daily lives and HBM; (ii) their concerns regarding exposure to chemical substances; (iii) beliefs towards chemical exposure and safety, as well as regarding HBM. Altogether, the results from each of the four group discussions are valuable contributions to support the design, implementation, and communication of future HBM actions, as well as to bridge the gap between science and citizens.

## 2. Materials and Methods

This is a cross-sectional observational qualitative study, with data collected through semi-structured focus groups conducted in four European countries: Austria, Portugal, Ireland, and the UK. In each country, one focus group composed of citizens was moderated by HBM4EU partners. Data collection took place in Austria (February 2018), Portugal (May 2019), Ireland (September 2019), and the UK (October 2019).

A qualitative approach using focus groups was employed to provide an in-depth understanding of citizen’s knowledge, beliefs, and attitudes in terms of chemical exposure [35]. It was felt that a quantitative approach would lose depth and meaning, and may omit key issues of relevance to policy makers. This is also particularly appropriate, as there have been few qualitative studies of human biomonitoring. Focus groups can provide richer information about the knowledge, attitudes, and experiences of the participants. They obtain information from the interactions with and between the participants. Their intent is

“not to infer but to understand, not to generalize but determine the range, and not to make statements about the population but to provide insights about how people in the groups perceive a situation”

[36], (p. 66).

Theoretical saturation is under discussion with four focus groups undertaken; similar thoughts from different focus groups do reflect that some common concerns are cross-sectional to different countries. Apart from the topic(s) under discussion, intensive phases of interaction indicate that the topics are key and relevant for the participants in the group discussion [37].

### 2.1. Participants: Sampling and Inclusion/Exclusion Criteria

Participant recruitment followed a purposive sampling (non-random, conceptually driven) in each of the four countries. Eligible participants were adult citizens (18+ years old), both sexes. An effort was made to ensure heterogeneity regarding age, educational level, urban/rural residence, and professional background, in order to gather different perspectives about the topics under discussion. Citizens were excluded from participating in focus groups if they could not master the language that the focus group was conducted in (German, English, or Portuguese, according to the country). This was also the case if participants were not able to provide written consent to participate in the focus group. Participants were invited to take part in the focus groups through different ways, according to the country. Selection from a panel of citizens from previous qualitative studies followed by invitation via email (Portugal), invitation via email and through online posts on institutional homepages or social media accounts of the HBM4EU institutions (Ireland and Austria), invitation via citizen pool from occasional citizen dialogues and through snowball principle (Austria), invitation from a long standing people panel (UK) facilitated by Public Health England (PHE), and invitation from advertisements posted in public/community buildings within the adjacent geographical area of the focus group venue (Ireland). Sample size ranged between seven (UK) and fourteen (Austria) participants. A total of four focus groups were undertaken. This provided sufficient data to analyse themes. A review of 40 focus group studies [38] found that 80% of all themes could be identified within 2–3 focus groups and 90% by 3–6 focus groups.

### 2.2. Setting, Procedures and Instruments of Data Collection

Focus groups took place within the facilities of the research units or local institutions. A moderator and a co-moderator, both experienced in these types of group discussions, conducted the focus groups. The co-moderator assumed a less active role in the discussion and was mainly responsible for introducing topics not covered by the moderator and for taking field notes. The session started with the moderator seeking consent to audio record the discussion, followed by an overview of the main sections to be discussed (Table 1) and a five-minute introduction to the HBM4U project. Part of the focus group topic guide involved participants having to imagine that they were in the year 2023. This was used to encourage discussion on potential changes and impacts that the HBM4U project may have had, and the best way to disseminate information. The moderators assumed a non-directive semi-structured approach, proposing the topics for discussion and promoting group interaction. The topic guide questions were flexible in terms of order and depth of discussion, so national teams could reorganize the discussion to reflect local needs. The duration of the focus groups was variable and ranged from one to three hours (full session). Participants were given light refreshments during the sessions, which also contributed to “break the ice” and promote group interaction.

The full sessions were audiotaped and fully transcribed. The transcript and text material were rechecked prior and during the coding process by at least two experienced researchers (transcripts of FG and code list can be found in Supplementary Material).

### 2.3. Data Analysis

Content analysis was independently undertaken by two researchers of the study team, per country, following grounded theory principles [39,40]. Each researcher independently read the transcripts, noting key words or phrases (open coding). These were used to construct a coding framework of similar categories. The coding framework was triangulated for interpretative validity [41] by the moderators and co-moderators of the focus groups. Conceptual triangulation was enhanced by a review of the literature. The coding framework was then used to identify key themes emerging from the data gathered in the group discussions. Further interpretative and significance validity was assured by providing open access to the anonymized corpus (enclosed raw text data of transcripts is provided as Supplementary Material).

### 2.4. Ethical Issues

Prior to taking part in the focus groups, all participants signed an informed consent form. Within that, it was explained that participation was voluntary, that they could withdraw at any time and that all data collected were anonymous and confidential. The focus group moderator also sought consent to audio record the discussion and asked for participants’ permission to take photographs during the group discussion. The conduct of the focus groups in HBM4EU was approved by the project management board. Adherence to rules of the GDPR (General Data Protection Regulation) was assured by the project teams carrying out the focus groups in accordance with national policy. In all focus groups, citizens gave informed consent through a declaration of consent on data protection of the affected persons (according to Art 6 Para 1 lit a GDPR) Citizen Workshop on Human-Biomonitoring in the EU. Additionally, in Portugal, positive approval for conduction of the focus group was obtained by the Ethics Committee of the Centro Académico de Medicina de Lisboa (Reference 306/19). Ethics committee approval was not required in the other three countries.

## 3. Results

Sample characterisation, by country, in terms of sociodemographic variables, is provided in Table 2.

### 3.1. Citizens’ Perceptions and Concerns about Chemical Risks and Their Impact on Human Health

In the following section, specific statements of participants are cited and discussed in relation to current challenges in risk assessment and management. Participants were concerned about the health risks associated to the exposure to a variety of chemicals, namely through various sources of exposure they are exposed to in their daily lives and via consumer products and food. Few participants named specific chemical substances; asbestos and phthalates were mentioned in the Portuguese focus group, whereas diesel fumes were referred to in the Irish focus group. Some participants expressed their general concern about polluted water and industry-related air pollution (visible steam).

“It’s those things that the plastics we use in our daily life have… the leaching into the beverages we drink, the food we eat, the food we heat in the microwave, inside of plastic boxes, all those contacts we made, as stated before, also through the clothes we wear, what we touch, and the effects even to our offspring”

(Portugal, male, lines 108–114).

“…we often walk Sandymount Strand with our dog and there is a non-ending plume of what is supposed to be steam coming from it; but what’s in steam? But I presume that EPA are monitoring that.”

(Ireland, male, lines 31–33).

In one focus group, participants identified main exposure pathways to chemicals (e.g., outdoor air, food, drinking water, and clothes) and established a link between exposure pathways and different sources of pollution. For example, car exhaust emissions and car brake dust were linked to chemical exposure through outdoor air. Pesticides used in crops and flavourings, preservatives, and colour additives used in soft drinks production were linked to chemical exposure through food; environmental reservoirs of antibiotic resistant microorganisms and industrial wastewater discharges were linked to chemical exposure through drinking water.

Two groups mentioned mixtures of chemical substances and interactions between these substances:

“…you know I used shampoo this morning, and I have a toothpaste which I believe is better than the fluoride ones, but how do I know is there something still in that toothpaste that is not good, and I tried to have as little as possible in terms of makeup, and you know, I am using really good pure baby-type creams and things like that, but what about all the stuff I do not know about.”

(Ireland, female, lines 17–22).

“…was mentioning about things in combination as well. Mercury with other exposures seems to have more of an effect. How can this be monitored? What do we do about mixtures.”

(UK, male, part 2, lines 255–256).

Some participants in another focus group identified the “chain of effects” of the human influence on the whole ecosystem:

“One of the one of the things that I think goes on in research that you do is you look at single chemicals. And one of the things that I think perhaps isn’t looked at enough is the cocktail of chemicals that we’re absorbing. Because we seem to have a lot more stuff that’s going on. And the, it’s actually how, how, what, what can be done about that, for example…”

(UK, female, part 1, lines 577–581).

“…when you get in things that are coming in…outside the EU, for example, from South America, taking the example of grapes. What? How do we know they’re not filled with pesticides?”

(UK, female, part 1, lines 610–611).

Indirect exposure from using products that were in contact with potentially hazardous substances at any stage of the production chain was also an expressed concern. For example, Irish and UK citizens were concerned about the use of pesticides to protect crops from pests, especially in the case of fruit production:

“but if you cut an apple from the shop in half, it stays perfect, so what is making that stay perfect, there something, you know, whereas when they just pick it off the tree with no pesticides or anything, it would start, the minute you cut it, it would start to go brown, but when you buy them, they don’t, they stay like that.”

(Ireland, female, lines 57–61).

A UK citizen mentioned farm animals and the use of antibiotics and antibiotic resistance:

“…factory farmed animals are routinely fed antibiotics as a prophylactic, rather than actually to treat condition and those antibiotics, presumably and I’m saying presumably because of, I don’t know, got access to research, but they’re in the meat and therefore when we eat that meat we’re taking in antibiotic.”

(UK, female, part 1, lines 584–587).

A Portuguese participant mentioned the irrigation of agricultural fields using potentially contaminated water from the rivers that is captured through artisanal boreholes:

“My main concern is not so much about direct consumption, which I believe is well regulated and well controlled, but it is much more about indirect consumption, that is, water that is used to irrigate the crops we later eat and that is not so scrutinised, it is often taken directly from the river or another watercourse (…ah…) and also in Portugal, the percentage of people who still only use water pumped from artisanal boreholes is higher than expected.”

(Portugal, male, lines 270–276).

Although participants generally expressed some concern in terms of exposure to environmental pollution, some participants pointed out that it is important to avoid alarmism, as this can be equally harmful to health. According to them, it is important to establish trust in the governing authorities instead:

“Because it makes us worried and when we are worried, we live less. I guess.”

(Portugal, male, line 139).

“I always guide myself by the way I trust or not in the authorities that regulate it.”

(Portugal, male, lines 231–232).

### 3.2. Communicating Information about Chemical Exposure

Participants in the four focus groups acknowledged the key role of communication in empowering citizens to take action. It empowers future consumers’ decision-making and opens possibilities for them to put pressure on regulatory authorities. The depth of the discussion on communication of information on chemical exposure and HBM varied across groups, with some participants referring to generic types of communication (such as websites, articles in newspapers) whereas others mentioned specific types of communication.

“We would need a database available to the public, one we can gather more information from. What is happening? What is it about a certain substance? What does it do […] with a certain chemical? Empowerment!”

(Austria, female, lines 282–284).

Anecdotal evidence of citizen empowerment after transparency in information communication was provided by a Portuguese participant:

“We had phenomena like the history of mad cow’s disease, etc., if it weren’t for public pressure, maybe we still ate contaminated beef today, wouldn’t we?”

(Portugal, male, lines 785–787).

**Figure 1:**
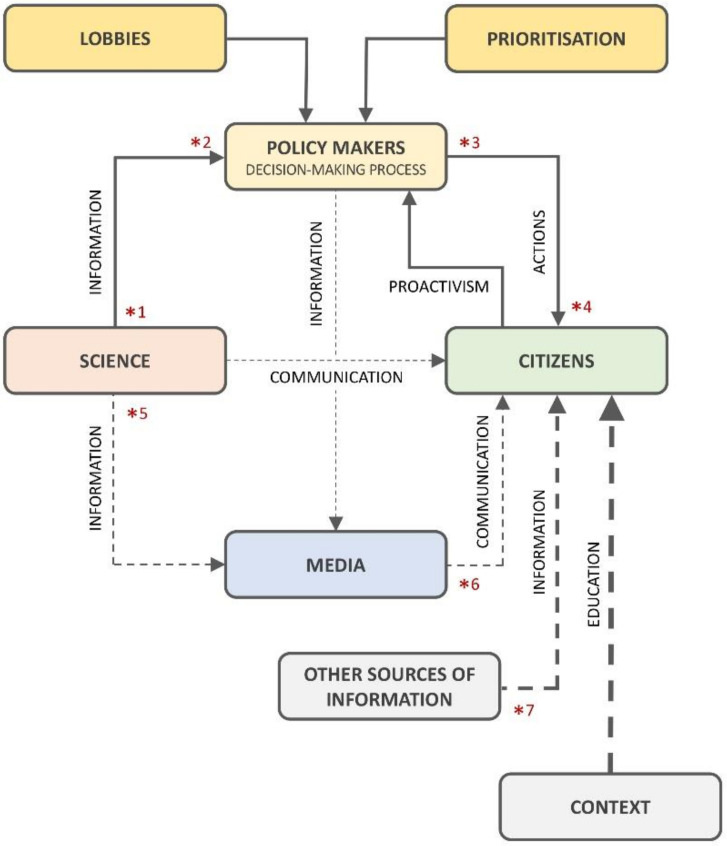
Model of information flow and communication of HBM knowledge (communication from media and science, information from other sources, and education from the context, all together seems to affect, at different levels, citizen perceptions on the health impact of chemical exposure. The asterisks indicate critical points related to the way information is emitted or received by the stakeholders. Continuous lines represent that information or actions flows in closed circuits, i.e., not taken into consideration citizens perceptions or needs. Dotted lines indicate an open flow of information to which citizens are exposed to. The thickness of these lines is related to the level of influence on citizens’ perceptions and attitudes. Therefore, the context (subjectively understood here as the combination of individual, social, and place attributes) seems to have more influence than other sources of information (e.g., social media), media, and science communication) (empirical data driven model: citizen perspective).

A model of the communication process in HBM involving science, decision makers, and citizens emerged from the Portuguese focus group. Participants mentioned that science produces information that is used to support decisions by policy makers, ultimately leading to action. Interestingly, national authorities, such as the Portuguese Environment Agency, were not identified as being part of this communication process. The role of lobbying in the prioritisation process of political action offered some concern to the Portuguese participants in the group discussion, as well as the role of citizens, who were considered as passive receptors of political decisions that, according to them, follows a top-down approach. Overall, Portuguese participants in the focus group felt that communicating scientific results was a very important component of the “communication triangle of science” (Figure 1). Disseminating information to the public may contribute to increased citizen engagement, which in turn may empower them to put pressure on politicians to take effective actions that shall be oriented to meet citizens’ needs. Ultimately, this contributes to reduce mistrust in politicians.

“First, reach the public and then the public put pressure on the politicians.”

(Portugal, male, lines 1025–1026).

Threats to transparent and credible scientific data communication were also discussed. Participants in one of the group discussions stressed that scientific information should be communicated using credible sources, such as science experts. This was an important step to provide “certainty” and to protect citizens and public opinion from “fake news”. The environmental campaign led by Greta Thunberg was given as an example of an approach emphasising the importance of science and the need to “listen to the scientists”.

Participants in the UK focus group identified the need for executive and government agencies to produce detailed, focused, and tailored information to be delivered to the citizens. Some participants expressed their opinion that citizens should have “a right” to receive information when adverse human health effects are detected from chemicals to which they are directly or indirectly exposed to. A list compiled by medically trained individuals, providing information about hazardous and less harmful chemicals (e.g., products where the chemicals can be found, risks to particular groups), should be produced and delivered to the general population.

“Public Health, I think has a high priority for most people. Not just their own personal health, but the health of the population at large. For all sorts of reasons, I think it’s a good thing that people are more aware than was the case in my parents’ generation, or even my grandparents’ generation, they simply weren’t aware of these issues.

(UK, male, part 2, lines 722–725).

“…the public, I think, are becoming more and more aware of pollution, and heavy chemicals in water and food and plastic in the ocean, and all of these kinds of things. And what we need, I believe, is a guide from government or from executive agencies, or maybe both about a broad ballpark area where we can look for more information on these topics, and perhaps specific websites, and where we can find answers to questions. So I think the objective communication in this realm is to make the public more aware. And more aware in a more focused, targeted way than government is generally very good at so that we can begin to ask the right questions of people like you (PHE scientists).”

(UK; male, part1, lines 481–491).

“I believe we need to lead from medical practitioners, people with medically trained who can say there’s a whole load of chemicals in the air in the soil, we eat them, breath them, drink them, many of them are not dangerous, but some are and here’s a list of them.”

(UK, male, part 1, lines 705–707).

One person stressed that there was a need to build-up trust and confidence in government organisations. Several others felt that information should be provided by non-governmental organisations. One focus group highlighted the importance of an independent “watchdog” organisation that had authority:

“So, if we have governments looking to independent bodies like an independent water authority, you know, that is monitoring waters, water pollution levels.”

(Ireland, male, lines 362–364).

The need for the HBM4EU project to provide information, namely evidence on health effects from chemical exposure, in a user-friendly format, easily understandable by the general citizen, was stressed by the participants in the Irish focus group. One participant, holding a Master’s degree in biochemistry, commented that some information provided, e.g., on a chemical safety data sheet he had seen, was “totally unintelligible.” A different participant stressed that it would be important for this project to turn “scientific knowledge into understanding”.

“…we are going to have it in language a five-year-old can understand.”

(Ireland, male, lines 289–290).

### 3.3. Barriers to Reduce Pollution Levels

Pollution was identified by the participants in the focus groups as one important pathway of exposure to chemicals. As such, the discussion not only focused upon concerns about health effects after exposure to sources of pollution (see Section 3.1 Citizens’ perceptions and concerns about environmental risks and their impact on human health), but also several barriers that prevent active action to reduce pollution levels. Different expectations from different stakeholders were emphasised across focus groups as a main potential barrier to reduce pollution levels that could be surpassed if the stakeholders engage in a collaborative effort at many different levels. The government was pointed out by some participants as a barrier, because they would protect the industry over citizens’ interests. The rationale was that the industry sector creates jobs and contributes to the economical wealth of the country. As such, measures aimed at reducing pollution levels mainly target the consumer and less is required in this realm from private companies.

The role of “lobbyism” and the industry sector’s own agenda and interests in shaping political decisions was referred in two out of the four focus groups by some of the participants. Additional industry-related barriers were identified by the participants: Aggressive marketing strategies were mentioned as contributing to an overload of messages that makes it harder to distinguish relevant and credible information about hazardous substances. Constant product innovation by industry in highly industrialised areas of the world was also questioned: according to some citizens, this has led to innovative products based on new substances, whereas the actual need for constant product innovation was not always clear to everyday consumers, even though these products might offer more convenience for the consumer and profit for industry.

“…there are probably other chemical industries that are being protected because they bring in money.”

(Ireland, female, lines 98–99).

“Money drives so much. That is very, very sad at the expense of its own citizens…”

(Ireland, female, line 106).

### 3.4. Facilitating the Reduction in Pollution Levels

Several participants mentioned that science (and human biomonitoring in particular) had an important role to play regarding the reduction of pollution levels. For example, scientific research should be undertaken to find alternative substances that are less harmful to the environment and to human health. Participants noted that results of biomonitoring initiatives could be used to convince both politicians and industry about the need to regulate, deregulate, further investigate, and ban hazardous substances to human health and the ecosystem.

“Do we always need an innovation? So, do we have to develop new substances that we have to test again to find out whether they are ok, or maybe we can take a look and see what else is there?”

(Austria, female, line 190f).

“I think this initiative must be a bridge between the government and the industry. That matters a lot, I think.”

(Austria, male, line 371).

Some participants expected that biomonitoring would provide a better understanding of disease aetiology and the potential to reduce risk through changing behaviour. Others also expected that this scientific information would translate into policies and effective actions aimed at protecting human health. In one focus group (UK), all participants agreed that the government should prioritise a biomonitoring programme. It was felt that public health was a priority for most people, and such a programme would help raise awareness and inform the public in terms of what the government is doing to improve health—as an example of good governance. Some also highlighted the potential of biomonitoring to be used in longitudinal studies to monitor pollution exposure over time.

“One thing that would be interesting and I don’t know if it is already being done, and this would be to follow the exposure process of an individual over time, [that is] (…ah…) a study that started when the individual was a baby, or with breast milk or anything, and those kids would be followed, for example, with a new wave of data collection at 10 years old, a new wave at 20, a new wave at 30, in particular for people that lived in that area, and whom do not move that much around the world, so that data obtained, especially if they wanted to investigate the effect of some factory in the area or something.”

(Portugal, male, lines 523–530).

“I think the public actually is trying to form this question. What is it that you guys, whoever you guys are: GPS, our hospital, hospitals, governments, local authorities, school teachers, whoever you are, what is it that you’re doing to improve our health as a population or as a group? And I think a lot of people would like an answer to that question.”

(UK, male, part 2, lines 732–735).

Involving the public in research, in terms of feedback processes, was highlighted by several participants in one focus group as a mechanism that could be used to demonstrate to politicians the level of public interest in pollution prevention. This could facilitate further scientific research regarding harmful substances.

## 4. Discussion

Citizens’ awareness and perceptions of chemical products has been a topic of interest to the European Union for more than four decades. National human biomonitoring programmes that simultaneously monitor, improve chemical management, and better inform the public about the risks of exposure to chemical substances are running worldwide, such as the United States National Health and Nutrition Examination Survey (NHANES), the Canadian Health Measures Survey (CHMS), the Korean National Environmental Health Survey (KoNEHS), the German Environmental Survey (GerES), the French Longitudinal Study of Children (ELFE), and the Flemish Environment and Health Study (FLEHS).

Whereas human biomonitoring studies of high quality have been performed to assess the presence and concentration of chemicals in human biological matrices, there have been fewer qualitative studies of human biomonitoring. The few available have mainly focused on issues, such as evaluation of communication materials [42], educating the public and making recommendations in terms of human biomonitoring surveillance [43], assessing risk communication strategies associated to human biomonitoring campaigns [32], ethical challenges in biomonitoring results communication [44], public perception of chemical risks [45], or getting feedback in terms of specific human biomonitoring methods [46]. By gathering in-depth feedback from citizens, our study provides valuable insight into their level of knowledge about human biomonitoring, as well as their perceptions, attitudes and concerns regarding the impact of chemical exposure in their daily lives and health. The citizens pre-knowledge, different perceptions on environmental issues, individual contextual experiences do also have an impact on results achieved (compare section of sample characterisation).

The results of the focus groups conducted in Austria, Portugal, Ireland, and the UK reveal a general concern regarding chemical exposure on citizens’ health and their daily lives. This concern is grounded on the perceived risk and the perceived impact on both individual and community health, which seems to be linked to the way each one assesses the exposure to certain chemical substance, independently of its toxicity, and the context this exposure takes place. Participants expressed their concerns using heuristics and narratives from their own daily experiences, believing there is a cause–effect relationship between chemical exposure and health. Several studies have demonstrated that living near solid waste incinerators, industrial plants, or cropland with pesticide applications is related to an increased risk of adverse health outcomes [47]. However, other studies have found that there is a mismatch between beliefs about exposure and beliefs about toxicity [27], suggesting that public perception of chemical risks is mediated by people-related and place-related factors. Our results help reinforce these findings by providing a vivid description of human exposure in citizens’ everyday activities, such as taking a shower and eating food. Both direct and indirect exposure were discussed, as well as the impact of chemical exposure on future generations.

Knowledge levels, in terms of human biomonitoring and chemical exposure, varied between participants and different focus groups. Some were able to identify links between sources of exposure (such as chemicals in everyday products, industrial emissions) and their pathways (how an individual is exposed to chemicals, such as through food, air, drinking water, or consumer products). In addition, several demonstrated some level of understanding on human biomonitoring, although no participant provided an accurate definition. One participant pointed out that the extent of exposure to a chemical substance is also an important issue, claiming the need for longitudinal studies to monitor the health effect of long-term exposure to low-doses. The existence of human biomonitoring studies was recognised as a positive sign that authorities are actively monitoring the risks. This seems to contribute to make authorities trustworthiness and to give people some sense of relief.

Trust was addressed as a major concern. Some citizens felt that governments protected industry due to their role in the economy, with emphasis placed on the consumer as opposed to industry in terms of preventing exposure to chemicals. There was also a distrust in politicians reported in one focus group where lobbying was seen to influence political decision making. Indeed a new model of information flow and communication was developed by participants in this focus group that emphasised the importance of political lobbying, with national environmental authorities having little involvement in communication and the citizen having a passive role. The Eurobarometer 314, which assesses European attitudes toward chemicals in consumer products, reported that European citizens place the greatest trust in the European Union (35%), followed by the national authorities (32%) and the industry (21%) [48]. In fact, public trust toward industry and regulators has been declining since the 1980s, which impairs the risk communication [49].

High levels of trust reduce the perception of risks and increase the adoption of protective behaviours. During the H1N1 pandemics, van der Weerd et al. [50] reported that higher levels of trust in the Dutch government, but also fear/worry and perceived vulnerability, were all positively related to an intention to accept vaccination. In the case of COVID-19 pandemics, a global survey conducted in 19 countries have shown that higher levels of trust in information from credible sources were associated to a higher acceptance of a vaccine [51]. The credibility of an information source has been widely studied [52,53] and some authors have tried to identify its attributes, which includes expertise, objectivity, impartiality, fairness, trustworthiness, and goodwill [54,55]. Moreover, Ryu et al. [56] have shown that source credibility influence both trust in government and trust in regulation, and consequently the perceived risk. Therefore, trust and credibility are two critical factors that should be seriously considered in the development of a human biomonitoring communication strategy. The higher the credibility of an information source and the social trust in authorities, the higher the possibility of citizens to adopt protective measures and to accept regulations.

Communication was acknowledged by participants to be crucial. The way information is communicated and received by the different stakeholders—science, policy makers, citizens, and media—was seen to have a pivotal role in the quality of the policies and the public reactance to them. Some participants highlighted the unintelligibility of the information communicated by science and authorities, which is viewed as a barrier to the public understanding of what is being transmitted. Risk information was also pointed out as something that needs to be improved. Current information provided by agencies was felt to not sufficiently satisfy. There was a need to provide information, in a context-dependent manner that would help protect against misinformation and “fake news”. The designing of risk information has been studied by some authors, with emphasis placed in the message (expected to be clear), the source of the message (credibility and trust), and the target audience of the message (who is the target public) [57]. Once again, the source of information seems to have a crucial role, not only in terms of its credibility and trustworthy, but also the medium in which it is transmitted. According to the Eurobarometer 314, more than half of the European citizens would prefer to get information on safety and safe use concerning chemicals in consumer products directly from the product label and package information (54%), followed by the television (39%), although in some countries, citizens would prefer the television as a source of information in first place. In terms of provision of reliable information, the Europeans trust more in the consumer associations (54%), while 43% considered that scientists and researchers provide the most reliable information [48]. These data highlight the need to know the profile of the target audience to shape the message accordingly, to communicate more effectively.

For many years, the public health community has based their communication strategies on the view that if any campaign fails in terms of changing people’s attitudes, then the problem is the lack of information [58]. Over time, the information deficit model has been supplanted by the ecological model, which considers that population health and environmental outcomes are determined by the behaviours of the people in the population. In turn, these behaviours are determined by people-related and place-related factors, including attributes of people (beliefs, skills, behavioural modelling, social reinforcement, social norms) and attributes of places (laws, policies, cultural, and media messages) [59]. A communication strategy must consider this people and places framework to be effective in its goals. Keune et al. [32], for example, have shown the importance of adopting a two-way risk communication focused on participation and cooperation between stakeholders (scientists, policy makers, and citizens). Findings from this study supports the rationale that risk perception is influenced by several factors and not only a result of information deficit (personal experience, environmental consciousness, related knowledge, media coverage, communication strategy, and source information credibility).

The need to provide information and to communicate more efficiently has been recognised by the HBM4EU project and by other European research and governmental institutions. HBM4EU is actively addressing this gap by producing citizen-targeted information, such as videos on human biomonitoring and chemical exposure and factsheets on chemicals, which are shared on its website and through its stakeholder network. Research and government institutions have also started to involve the public in citizen science initiatives as strategies to foster and enable collaborative data collection and prolong public debate. Such initiatives were also being explored by the European Commission with available funding through Horizon 2020, as well as by the United Nations with citizen science also playing a key role in the Sustainable Development Goals [60,61]. Curieuzen Neuzen (Curioous Noses) [62] and CleanAir@School are some examples of citizen science projects on air quality.

In terms of prevention of chemical exposure, a clear message from the focus groups is the important role of science. Science was viewed as the cornerstone to preventing chemical exposure, with one focus group identifying science as a core element of a communication process. Science shall address knowledge gaps and communicate risk to the public in a way that understandable to them. By raising awareness, science may help to promote trust in risk assessment and management procedures and institutions. Two focus groups identified that the role of science was not only to study, identify, and monitor potentially harmful substances, but also to translate scientific information into policies and actions aimed to protect human health. As such, scientists need to play a key role in translating and communicating their findings into resulting actions aimed at reducing chemical exposure. If science is to play a role in promoting actions stemming from research, then there will be a need to promote stakeholder cooperation and engagement. Science needs to be able to influence policy development and implementation and to disseminate information. Multi-sectoral partnerships have been recommended by the Lancet Commission on Pollution and Health [63] to control and mitigate the effects of pollution on public health.

The study has demonstrated the value of qualitative methods (e.g., focus groups) to inform policy makers at both a national and European level. Such information can be used to help protect people against the dangers of chemical exposure. The focus groups were also useful in terms of empowering participants and providing them with information. Some, for example, referred to the need for people to be proactive and to take the initiative to directly influence decision makers. Others (having been given information about the HBM4EU project) were hopeful that the results would be effectively translated into political measures for the common good. However, it is worth recognising that the study does have some limitations. It was not possible to undertake the focus groups at the same time point in each country and the guideline of the focus groups did not address any specific event in particular. During the time passed, there was not a major initiative that dramatically changed the way communication about biomonitoring for the general public happened. HBM4EU also provides tailored information on human biomonitoring to the public during the project and beyond. The fact that only one focus group per country was undertaken meant that it was not possible to generate results for specific countries. Moreover, it is arguable if the collected information is comprehensive enough to adequately understand the phenomenon under investigation. Nevertheless, this would be a matter of conceptual exhaustiveness, rather than an issue of representativeness [64]. The overall conclusion drawn from the four focus groups do show similar argumentation paths and are not too different from each other. Therefore, limitations of the study need to be acknowledged, but are not that significant. 

There was also variation in the length of each focus group (60–180 min) and the number of participants (7–14). Although refreshments were offered during the focus groups, lengthy focus groups may have led to participant and facilitator exhaustion. Focus groups with a large number of participants may not have given everyone the opportunity to provide feedback [65]. Not only the number of participants and time needed to conduct the focus group differed, but also the insight of participants on biomonitoring varied among the citizen. Next to different levels of insight, varying characteristics in people with different expression levels become visible in focus groups. This also affects the group dynamic and time used in the focus group. In addition, individuals from lower educational backgrounds were under-represented in the focus groups. Studies have shown that perceptions of environmental issues, such as air quality, are affected by education level, along with other socio-demographic factors, such as age and gender [66]. Further studies highlighted the need to raise awareness and interest in exposure to chemicals and environmental issues among lower socioeconomic groups [67,68]. It is important to engage with these groups in developing policies to ensure that issues important to them are taken into consideration. This may require additional focus groups that target different segments of the population.

## 5. Conclusions

This study provided valuable feedback, in terms of people’s exposure to chemicals, and their knowledge, concerns, and perceptions of biomonitoring, which may be useful for targeted communication plans in support of risk governance strategies. People do have concerns about the health impact of chemical exposure; more information is required, which should be disseminated in a way that is easily accessible and understandable to citizens. The overall acceptance of biomonitoring demonstrates its potential in terms of developing national programmes. Moving forward, science should play a key role, in terms of both creating knowledge and offering procedures that involve citizens at high levels, as they make stronger claims to be included in such processes, and ask to be provided with trustful, correct, and validated information. Finally, science should be part of a multi-sectoral approach, to develop policies and action plans for a sustainable future.

## Figures and Tables

**Table 1:** Guidelines for conducting the focus group: relevant topics to be covered.

1	Introduction on human biomonitoring	- Pre-knowledge on human biomonitoring - Relation to the topic - Special interest on human biomonitoring studies/previous participation
2	Human biomonitoring now: exposure to chemical substances in our daily lives	- Substances of concern - Assessment of chemical exposure - Health problems related to chemical exposure - Relevance of Human biomonitoring results and the Human biomonitoring (HBM) initiative for everyday life - If applicable, areas and reasons of relevance
3	Thought experiment: human biomonitoring project—the future	- Expectation regarding the results: content - Expectations regarding the results: impact (e.g., policy making)
4	Prospective future: results of the human biomonitoring project	- Perception of achieved results and applicability of the results - Who receives information about the project’s results and how (i.e., communication channels) - Preferred communication channel to obtain information about the project

**Table 2:** Sample characterisation by country.

	Portugal	Ireland	United Kingdom	Austria
**Gender**				
Male	5 (50.0%)	5 (45.5%)	3 (43%)	7 (50%
Female	5 (50.0%)	6 (54.5%)	4 (57%)	7 (50%)
**Age**				
Age range	(24–63)	(18–74)	(52–72)	(20–72)
Mean age ± SD	46.6 ± 14.6	Age cohorts were recorded	64.0 ± 6.9	41.3 ± 4.6
**Education**	University degree (8), Secondary education (1), primary education (1)	Third education level (9), secondary education (2)	Professional background was recorded	University degree (9), secondary education (5)
**Previous knowledge about HBM**	Participants claimed lack of information on the topic	Few participants were previously exposed to the topic, either through the media or at work	Knowledge mainly claimed on media consumption (TV documentaries, movies)	Almost half of citizens with professional or educational background on the topic, all claimed interest on this

## Data Availability

The data presented in this study are available in Supplementary Material (link).

## Supplementary Materials

The following are available online at https://www.mdpi.com/1660-4601/18/4/1532/s1, Transcripts, Integrated code list.
