# Supplementary material for: Chemical Exposure: European Citizens’ Perspectives, Trust, and Concerns on Human Biomonitoring Initiatives, Information Needs, and Scientific Results
Source: Int J Environ Res Public Health. 2021 Feb 5;18(4):1532. doi: 10.3390/ijerph18041532 (PMC7914422; doi:10.3390/ijerph18041532)
Supplement: Supplementary file 1 [file ijerph-18-01532-s001.zip › HBM4EU_citizen perspectives_supplementary material_22_12_final.docx]

Article

Chemical exposure: European citizens’ perspectives, trust and concerns on human biomonitoring initiatives, information needs and (scientific) results

Maria Uhl^1,*^, Ricardo R. Santos^2^, Joana Costa^2^, Osvaldo Santos^2,3^, Ana Virgolino^2^, David Evans^4^, Cora Murray^4^, Maurice Mulcahy^4^, Dorothy Ubong^5^, Ovnair Sepai^5^, Joana Lobo Vicente^6^, Michaela Leitner^1^, Silvia Benda-Kahri^1^, Daniela Zanini-Freitag^1,*^

1. Environment Agency Austria, Austria; [maria.uhl@umweltbundesamt.at](mailto:maria.uhl@umweltbundesamt.at) (M.U.); michi_leitner@gmx.at (M.L.) silvia.benda-kahri@umweltbundesamt.at (S.B.K.); daniela.zanini-freitag@umweltbundesamt.at (D.Z.F.)

2. Environmental Health Behaviour Lab, Instituto de Saúde Ambiental, Faculdade de Medicina, Universidade de Lisboa, 1649-028 Lisboa, Portugal; ricardoreis@medicina.ulisboa.pt (R.R.S.); osantos@medicina.ulisboa.pt (O.S.); avirgolino@medicina.ulisboa.pt (A.V.); jfcosta@medicina.ulisboa.pt (J.C.)

3. Unbreakable Idea Research, 2550-426 Painho, Portugal; osantos@medicina.ulisboa.pt (O.S.)

4. Merlin Park Regional Hospital, Galway, Ireland; david.evans@hse.ie (D.E.); cora.murray@hse.ie (C.M.); maurice.mulcahy@hse.ie (M.M.)

5. Public Health England, UK; dorothy.ubong@phe.gov.uk (D.U.); ovnair.sepai@phe.gov.uk (O.S.)

6. European Environment Agency, Copenhagen, Denmark; joana.lobo@eea.europa.eu (J.L.V.)

* Correspondence: maria.uhl@umweltbundesamt.at; Tel.: 01/313043605; daniela.zanini-freitag@umweltbundesamt.at, Tel.: 01/313043468

**Supplementary Materials:** The following are available online at www.mdpi.com/xxx/s1,

**1 Transcripts
Transcript of the Austrian focus group (March 2018)**

S.B: Wenn es jetzt keine direkten Verständnis- oder Rückfragen zum Projekt mehr gibt, oder gibt es welche? #0:10:54-11#

(w): Ich hätte vielleicht noch eine Frage. Was sind diese Fluor? #0:11:02-03#

M.U: Ja? Wo die sind? #0:11:-#

(w): Was machen die?

M.U.: Gesundheitlich, oder?

(w): mhm (bejahend)

M.U.: Also sie sind sehr, sehr langlebig. Sie bauen sich fast nicht ab so wie früher die Organochlorpestizide, bei denen man darauf gekommen ist, wie bei DDT und diesen Stoffen, dass sie sich nicht abbauen, was ein Problem ist. Ähnlich sind die Perfluor-Substanzen. Sie haben auch eine Reihe von gesundheitlichen, also von toxikologischen Eigenschaften, die unerfreulich sind und wo man auch annimmt, dass eine gewisse Belastung in der Bevölkerung besteht. #0:11:13-48#

(w): Mhm (bejahend). Und wo sind die drinnen? #0:11:50-51#

M.U.: Das Problem bei diesen Stoffen ist, dass sie generell ist natürlich sehr gute Eigenschaften für die Industrie haben, aber nicht nur für die Industrie, auch für die Konsumenten. Das sind Wasserschutz-Abweisende Stoffe, die zum Beispiel in Outdoor-Jacken sind, also Spuren davon, das ist diese Perfluor-Chemie. Sie werden aber auch in der Technik eingesetzt, weil sie eben so extrem widerstandfähig sind, also Wasser und Schmutzabweisend. Gerade das macht sie aber eben auch wieder gefährlich. #0:11:54-12:23#

(w)1: Danke. #0:12:23-12:24#

(w)2: Und ihr schaut euch nur diese Stoffe an? #0:12:24-26#

M.U.: Nein, nein. Also in der gesamten Initiative gibt es neun prioritäre Stoffgruppen. Jetzt gab es die nächste Priorisierungsrunde wo fünfzig neue Stoffe nominiert wurden, die wurden dann nach bestimmten Gesichtspunkten wieder priorisiert. Und es wird eine zweite Runde an prioritären Stoffen geben. Also wir beschäftigen uns nicht nur mit diesen Stoffen. #0:12:28-45#

(w)2: Das heißt? #0:12:45-46#

M.U.: Wir in Österreich haben gesagt, dass wir hier einen Schwerpunkt setzen, weil wir diese besonders relevant finden. #0:12:46-13:02#

w(3): Ok, also neun Stoffe werden schon gemonitort? Habe ich das richtig verstanden? #0:13:3-13:10#

M.U.: Ja und da gehören die auch dazu, genau. #0.13:10-14#

w(?): Und jetzt kommen da noch weitere dazu? #0:13:14-17#

M.U.: Ja, aber das geht nicht alles von Heute auf Morgen. Sondern, es muss erst #0:13:17-23#

w(?): (wirft ein): Ja, ja. #0:13:23#

M.U.: an diesen Protokollen gearbeitet werden. #0:13:24-26#

w(?): Ja, ja.

M.U.: Es ist noch keine einzige Probe im Rahmen von dem Europäischen Projekt untersucht worden. Es gibt jetzt einmal die vorbereitenden Arbeiten. Wo braucht man überhaupt Daten? Wir können nicht anfangen alle möglichen Dinge zu analysieren, über die wir vielleicht schon genug Daten haben. Also man muss wirklich sich überlegen was man untersucht. #0:13:26-46#

w(?): Und die Alternativen werden dann auch untersucht? #0:13:47-48#

M.U.: Die Alternativen werden zum Teil auch untersucht, wie bei den Weichmachern, zum Beispiel. #0:13:49-53#

w(?): Wie viele Probanden, also wie viele Proben werden da untersucht pro Land? #0:13:55-58#

M.U.: Also , um dieses europäische Bild zu erlangen, sind das insgesamt (überlegt) 1500 Proben #0:13:58-'14:10#

w(?): Pro Stoff? #0:14:11#

M.U.: Ja. #0:14:12#

w(?): In Österreich? #0:14:12-13#

M.U.: Nein, nicht in Österreich. Pro Land will man 300 Proben. Und es sollen alle vier Regionen abgedeckt werden, also West, Nord, Süd und Ost. #0:14:15-27#

w(?): Welche Parameter werden noch herangezogen um die Regionen vergleichbar zu machen? Man muss ja die Parameter pro Land dann auch vergleichbar mit den Proben machen, oder? #0:14:26-39#

M.U.: Ja #0:14:38#

w(?): Welche Umweltfaktoren werden zusätzlich noch gemessen um die Proben vergleichbar zu machen? Also gibt es da irgendwelche? #0:14:38-49#

M.U.: Im Rahmen von diesem Projekt werden keine Umweltproben gemessen, sondern wirklich nur die menschlichen. #0:14:50-#

w(?): (fällt ins Wort): Also auch nicht Nahrungsmittel... #0:14:31-56#

M.U.: Aber es gibt- #0:14:56-58#

w(?): Ernährungsgewohnheiten? #0:14:58#

M.U.: Das wird alles abgefragt. #0:14:59-15:00#

w(?): Also wird das Alles wird abgefragt? #0:15:00-02#

M.U.: Ja, das wird abgefragt. #0:15:02-03#

w(?): Und werden zusätzlich auch Wasserproben genommen? Aus dem Umkreis? #0:15:04-08#

M.U.: Im Rahmen von dem Projekt nicht. Aber es gibt schon sehr viele Daten und diese werden alle auf einer Internetplattform abrufbar sein. Also sowohl die Umweltproben, als auch die- #0:15:08-21#

w(?): (dazwischen): Ja #0:15:21#

M.U.: Ergebnisse der Human... (wird von w übersprochen) #0:15.21#

w(?):Das ist wichtig, weil ich sonst ja nicht evaluieren kann. #0:15:22-26#

M.U.: Ja. #0:15:30#

m(1): Ich wollte fragen ob es im Rahmen dieser Außenstudie um Naturproben geht und auch von Menschen? #0:15:30-38#

M.U.: Genau #0:15:38#

m(1): Auch in diesem Viereck? (unverständlich) #0:15:39-41#

M.U.: Genau #0:15:42#

w(4): Wird es nur Blutproben geben oder gibt es noch andere Untersuchungen? #0:15:45-51#

M.U.: Es wird Blutproben geben und auch Harnproben. Das ist von der Stoffgruppe abhängig, aus der die Stoffe gemessen werden. Im Rahmen von diesem Projekt haben wir wahrscheinlich keine anderen. #0:15:52-16:01#

w(4): Also Lunge oder so nicht? #0:16:02-03#

M.U: Lungen kann man schwer analysieren. Dazu bräuchte man Biopsie-Proben. Diese Art von Monitoring wäre invasiv. Am einfachsten ist noch Fettgewebe, aber das ist hier auch nicht dabei. #0:16:03-23#

w(3/4?): Kann man die Daten nicht teilweise aus den Sterberaten und so weiter heraus holen und vergleichbar machen? Oder nicht? #0:16:23-31#

M.U.: Welche meinen Sie jetzt? Welche Daten? #0:16:30-33#

w(3/4?): Also ich kann ja Sterberaten und dergleichen aus Bevölkerungsgruppen vergleichen. #0:16:32-35#

M.U.: (wirft ein): Ja, ja. #0:16:36#

w(3/4?): Und Krankheitswahrscheinlichkeiten ja auch. #0:16:38-42#

M.U: Genau. #0:16:43#

m(2): Eine ganz einfache Frage: Gibt es inzwischen eine Publikation? Wer, was, wie? #016:44-47#

M.U.: Zum HBM for EU? #0:16:48-52#

m(2): Von Ihnen zum Beispiel. (unverständliche Wörter) #0:16:52-53#

M.U.: Ja, von mir gibt es auch Publikationen aber noch nicht zu diesem Projekt. #0:16:55-17:02#

m(2): Interessant zu wissen. #0:17:02-03#

w(3): Also das ganze Projekt hat überhaupt noch nicht gestartet? #0:17:04-06#

M.U: Doch, es hat 2017 gestartet. #0:17:07-08#

w(?): Aha. #0:17:09#

S:B: Es gibt jetzt noch keine Publikationen. #0:17:10-11#

w(?): Ok. #0:17:12#

S.B: Auf der Homepage gibt es schon sehr viele Informationen, zu dem was bisher passiert ist. #0:17:13-14#

M.U: Es gibt natürlich auch schon Ergebnisse, in dem Sinn. Diese kann man sogar achon abrufen. Aber ich glaube es gibt noch Nichts was definitiv veröffentlicht wurde. Es gibt eine Steakholder-broschüre. Und es gibt natürlich verschiedenstes Material. #0:17:13-33#

w(?): Die Gruppen, die untersucht werden, stehen auf einer Homepage? Kann man sich die dort anschauen?#0:17:44-45#

w(?): Mhm (bejahend) #0:17:45#

S.B.: Gut, wenn es jetzt unmittelbar keine Rückfragen zum Projekt mehr gibt, dann würde ich gerne beginnen. Ich würde gerne mit einer Runde starten um zu erfahren ob Sie schon mal von Human Bio Monitoring gehört haben? Wie ist Ihr Bezug dazu? Und was halten Sie davon? Da würde ich gern eine kurze Runde machen wo jeder- Vielleicht haben Sie auch vor dieser Studie noch nie davon gehört. Es ist einfach für uns interessant zu wissen, wie wir in der Diskussion weg starten. Darf ich Sie gleich bitten zu beginnen? #0:17:50-18:24#

w(neu5): Ja, ich habe vor dieser Studie eigentlich noch nicht davon gehört. Also, dass das Human Bio Monitoring heißt. Davon wusste ich nichts. Aber ich wusste, dass Proben genommen werden und Schadstoffe geprüft werden. Ich habe mich jetzt noch nicht wirklich damit beschäftigt. Ich habe nur Plastik Planet gesehen. (lacht) Das ist das Einzige. #0:18:24-50#

S.B.: Danke#0:18:50#

w(neu?6): Ich habe auch vor dieser Studie noch nie von Human Bio Monitoring gehört. Also hat mich auch deshalb der Name sehr interessiert und worum es dabei geht. Ich habe mir gedacht, dass es sicher mit Lebensmitteln zu tun hat und Ernährung. Und deshalb bin ich auch heute gekommen, damit ich mehr darüber erfahren kann. #0:18:57-19:16#

m(2):Ich habe auch noch nie etwas davon gehört. Meine Erwartung von diesem Projekt ist eigentlich heraus zu finden wie das in Österreich ist, mit allen bewegungsgründen und Hintergründen. Weil ich finde das sehr wichtig. #0:19:18-4-#

m(3): Ja, Ich habe noch nie davon gehört und höre mir das jetzt einmal an. Meine Erwartungen wären eher, dass ich die Kontrollmechanismen interessant fände. #0:19:43-59#

w(7): Ich weiß von meiner Mutter, dass ihr damals abgeraten wurden zu Stillen. Sie ist jetzt knappe 70. Damals hat es geheißen, dass Muttermilch schädlich ist und dass da irgendwas drinnen ist, was man nicht sieht. Der zweite Bezug zu dem Projekt sind eigentlich diese ganzen Schaumbäder mit Glitter und so, welche ich jetzt auch im Hinterkopf habe. Das sowas eigentlich auch in das Blut geht. Das alles was so toll ausschaut, eigentlich super schädlich ist für uns. Und, ja, dann habe ich die S. bekommen und habe mir gedacht: Da muss ich mehr wissen. Das interessiert mich mehr. #0:20:00-36#

w(8): Ja, ich habe auch das Email bekommen und ich habe eigentlich schon gehofft, dass es solche Sachen gibt. Allerdings ist es nie zu spät anzufangen. Und ich denke das prinzipiell. Ich habe auch den Fragebogen gemacht, bei dem mir sehr viel bekannt vorgekommen ist, aber es ist einfach in der Bevölkerung (Mann redet unverständlich und leise dazwischen) noch nicht in das Bewusstsein gelangt, was wir eigentlich mit uns und unserer Welt tun. Und deswegen glaube ich ist das ein wichtiger Schritt. Vor allem wenn ganz Europa mit macht. Ich kenne diese Ringuntersuchungen auch von Lebensmitteln. Deshalb habe mir eigentlich gedacht, dass es das schon gibt. (lacht) #0:20:40-21:25#

S.B: Danke ##0:21:25#

w(8): Hallo. Ich finde das Thema total spannend. Ich habe früher in der Industrie gearbeitet, bei einem Verpackungshersteller, habe dort ein Sammelsystem injiziert. Ich bin sehr im Thema Plastik verankert. Ich komme aus dem Bereich der Öffentlichkeitsarbeit. Ich habe auch eine Ausbildung zum Wellnesstrainer gemacht, also bin ich sehr gesundheitsbewusst. Ich bin jetzt seit zehn Jahren Mama. Ich bin auch Seglerin und viel an der Adria unterwegs. Da sehe ich, wie die Adria am Beginn der Saison aussieht und dann am Ende der Saison. Und da sieht man wirklich nachvollziehbar wie der Mensch Einfluss auf die Umwelt nimmt, aber auch wie die Umwelt auf uns wiederrum zurück wirkt. Und ja, ich bin an allen Gesundheitsthemen sehr interessiert und finde es spannend, dass es das gibt und auch sehr wichtig. Die Bevölkerung gehört viel mehr aufgeklärt. #0:21:27-22:30#

m(4): Also ich habe von Human Bio Monitoring schon gehört. Und zwar von zwei Seiten: eine davon ist das ChairC (?). mein persönlicher Bezug dazu ist: Ich komme aus einer Chemiker-Familie, bin aber selbst kein Chemiker. Ich bin sozusagen…(Getuschel im Hintergrund) das schwarze Schaf. (überlegt) Mir ist noch im Ohr, dass mein Vater schon vor 40 Jahren gesagt hat: wir wissen kaum wie sich diese Substanzen, mit denen wir umgehen, auf unsere Gesundheit auswirken und irgendwann einmal wird es zu dieser Diskussion kommen. Und dann gab es die Diskussion über die Reach-Richtlinie, da ist mir das dann wieder eingefallen. Das ist mein persönlicher Bezug dazu. #0:22:34-23:26#

S.B.: Danke #023:29#

w(9- köönte 4 sein): Von Human Bio Monitoring habe ich noch nie etwas gehört. Ich versuche nur Umweltbewusst zu agieren und das interessiert mich schon sehr. Ich lese auch sehr viel dazu. Ich hoffe, dass ich da noch viel Neues erfahre .#0:23:33-49#

m(5): Ich habe grundsätzlich vorher davon noch nichts gehört. Das was mich bei solchen Sachen besonders interessiert ist einfach wie man es hin bekommt das Länder, oder Länder übergreifend ,oder auch im EU-Raum, ein wirklich standardisiertes Monitoring-Programm schaffen um vergleichbare Daten zu kriegen. Wie arrangiert man das? Und was steckt da dahinter? Und, letztendlich, was kriegt man dabei überhaupt heraus? Ist es wirklich der kleinste gemeinsame Nenner auf den man sich dann reduziert, oder sind das wirklich gute Daten die dabei heraus kommen und letztendlich auch irgendeinen Nutzen für die Bevölkerung haben, die ja eigentlich dahinter steht. #0:23:50-24:34#

w(undef): Ok

w(10): Ich habe prinzipiell schon von den Themen was gewusst. Mit Bio Monitoring habe ich mich aber bis heute noch nicht beschäftigt. Mir geht das so wie Ihnen: Ich habe gedacht weil auch viel in der Zeitung über Schadstoffe und so weiter steht, es wird ja öfter Etwas verboten oder darüber diskutiert ob Etwas verboten werden soll, dass das eigentlich schon gemacht wird. (lacht) Jetzt schaue ich mal was ich noch darüber lernen kann. #0:24:40-25:08#

S.B.: Danke #0:25:10#

m(6): Ich habe auch noch nicht davon gehört. Mir geht es ähnlich wie der da hinten und denke mir es wäre interessant zu wissen was wir mit den Daten anfangen können, wie wir sie auswerten können und wie wir sie kriegen. Das wäre das was mich am meisten interessiert. Das sind eigentlich folgende Fragen: Wie weit die Stoffe in die gesamte natürliche Konstruktion eingreifen, was die Konsequenzen sind wenn wir da Parameter trennen, ob man überhaupt Parameter trennt. Das wäre interessant heraus zu finden. #0:25:12-35#

m(7-mögl1?): Für mich ist das überhaupt Neuland. (räuspert sich) Ich muss aber dazu sagen, dass ich an sich an Umweltschutzthemen sehr interessiert bin. Meine Gedanken dazu sind, das was mein Vorredner auch gerade gesagt hat: Was geschieht mit dem Produkt oder was geschieht mit dem Endprodukt? Kann man die Politik dazu bewegen, unter Umständen die entsprechenden Schritte zu setzen? Da brauche ich nur einen Blick über den großen Teich zu machen, dort wird stark zurückgeschraubt bei Umweltthemen. Das hat zwar jetzt mit dem hier im Konkreten nichts zu tun aber immerhin die Gefahr, dass die Politik nicht versteht wohinauf die gesamte Entwicklung gerade durch den Einsatz von Chemie läuft, sehe ich kommen. Und daher habe ich mir gedacht, ich nehme hier einmal Teil. Und kann so vielleicht in einem Teilaspekt ein Bisschen was mitbewegen. #0.25:37-26:29#

S.B: Dankeschön. #0:26:33#

m(8): Also ich bin sozusagen der Gegenpol von ihm; Ich bin Chemiker (allgemeines Gelächter) aus einer Nicht-Chemiker-Familie (Gelächter). Allerdings so ganz stimmt das nicht, da nahezu alle meine Verwandten, respektive meine Eltern und Großeltern Bauern gewesen sin. Das heißt, dass ich jetzt seit 45 Jahren beobachte wie sich der Einsatz der Industrie auf die Landwirtschaft auswirkt (pausiert). Heute heißt es Monitoring, früher hat man Auswertung oder Beobachtung gesagt. Von Bio Monitoring selber habe ich schon mal gehört. Onkel Facebook, oder Tante Facebook, hat mir dazu etwas vorgeschlagen und da habe ich mir gedacht einmal im Leben kannst du auch sowas anklicken. (Gelächter) So ein Zeug. Und nachdem mich das Thema interessiert. Ich bin auch Imker bin, daher wären für mich auch die (unverständlich) nicht gerade uninteressant. Das wäre dann auch eine Frage von mir, da so etwas ja auch mal in einer Monitoring Geschichte vorkommen könnte. Das ist also mein Zugang, auch wenn ich heute, das muss ich zugestehen, nicht mehr in der Chemie, als Chemiker, arbeite. Nur vom (unverständlich) (Gelächter) #0:26:35-28:10#

w(?): (unterbrechend) Gibt es da Proben? Nimmst du Proben mit? (Lachen) #0:28:11#

m(8): An dem koche ich auch noch. Nein im Moment gibt es keine Proben. Der Nächste wird erst in ungefähr in einem halben Jahr fertig und vom letzten ist schon fast alles vergeben (traurige Geräusche im Hintergrund/Lachen). Ich bin kein Groß-Imker. Ich bin zwar groß, aber kein groß Imker #0:28:14-28#

S.B: Dankeschön. Danke für diese erste Runde. Die Frau U. hat uns erzählt, dass diese Human Bio Monitoring Initiative jetzt vier Jahre laufen wird, und schon ein Jahr läuft. Jetzt würde ich gerne ein Gedanken Experiment mit ihnen Unternehmen: Wir beamen uns nach 2022, also wir sind jetzt alle um 4 Jahre älter und diese Initiative ist abgeschlossen. Welche Ergebnisse sollte diese Initiative Ihrer Meinung nach geliefert haben? Was erwarten Sie sich, das wir 2022 haben? Oder was erwarten oder wünschen Sie sich? Bitte! #0:28: 32-29:19#

w(8): Was ich mir wünsche, aufgrund meiner persönlichen Erfahrungen und da ich aus dem Bereich der Wellnesstrainer komme, ist die Aussage: Es sind sieben Milliarden Menschen auf der Erde, die siebe Milliarden unterschiedliche Körper haben. Unsere Gesundheit ist immer eine Folge von der DNA und von unserer Ernährungsweise. Das was für Sie passt muss nicht für mich passen, und umgekehrt. Jäh wichtiger und sorgsamer eine Mutter mit ihrem Kind umgeht desto besser funktioniert die Rückkopplung zum Kind. Ich wünsche mir, dass man dem Kind einfach sagt : ,, Du ich bin erblich so und so vorbelastet.‘‘ Das einfach Information stattfindet und diese Information auf eine Art und Weise funktioniert, die nicht Angst streut sondern eher in das Positive verkehrt wird, so dass jeder mehr oder weniger auch persönlich seinen Gesundheitszustand beeinflussen kann oder könnte: Oder auch welche Dinge sozusagen ein No-Go für ihn sind. Ich wünsche mir auch, dass die Menschen wieder mehr Verantwortung in ihre Hände bekommen, ohne dass sie jetzt ins Grüne-Eck oder in irgendein Industrie-Eck gesteckt werden. Einfach das begriffen wird, das Gesundheit ein individueller Zustand ist. Das würde ich mir wünschen. Das man auch nicht immer nur die Industrie in das böse Eck stellt. weil viele. Unsere Lebenserwartung wäre nicht so hoch geschraubt ohne Industrie, zum Beispiel. #0:29:24- 30:52#

S.B: Und was hätte diese Initiative dazu getan, dass es 20122 so ist? #0:30:53-31:00#

w(8): Die Individuellen Einflussmöglichkeiten sollen in den Studienergebnissen auch mitkommuniziert werden. Das heißt, das man weiß was es heißt wenn man in der und der Ecke wohnt. Es gibt ecken da gibt es zum Beispiel natürliche Radioaktivität. Dasa die Menschen auch bewusst informiert werden die Bevölkerung einfach sensibilisiert wird, aber auf eine positive Art. #0:31:03-27#

m(älterer): Da möchte ich aber dazu sagen. (lacht) Grundsätzlich haben Sie recht mit der Sensibilisierung. Nur es hat ja doch in der Vergangenheit immer wieder den Aufruf gegeben, dass man sich zum Bespiel gegen schlechte Lebensmittel wehren soll. Nur wenn einer oder zwei sich wehren hilft das gar nichts. Wie kann man also wirklich, um bei dem Bespiel der schlechten Lebensmittel zu bleiben, die Lebensmittelindustrie dazu bringen, nachzudenken, und nicht die Gewinnmaximierung im Vordergrund zu haben, sondern darüber nachzudenken wie wir die Weltbevölkerung ernähren. Weil Sie die sieben Milliarden Menschen angesprochen haben komme ich zum nächsten Problem: Wie schaffen wir es überhaupt im Jahr 2022 so viel an Grundnahrungsmitteln zu haben, dass wir alle versorgen können? Aber das hat jetzt wieder mit dem Thema hier nicht unmittelbar was zu tun. Ich erwarte mir von dem Ergebnis dieser Studie, dass vielleicht Erkenntnisse gesammelt werden wie sich diese Perfluor-Substanzen auf die Individuen und auf die Lebensqualität der Menschen wirklich auswirken. So das man dann vielleicht schon eine Aussage darüber machen kann. #0:31:28-32:50#

m(6): Da möchte ich mich auch anschließen. Ich teile ich die Überzeugung meines Vorredners vollkommen. Wir sollten zuerst herausfinden was die Stoffe machen. Wissen wir bei den Meisten schon was sie tun oder nicht? #0:32:52-33:00#

M.U.: Nein, also das ist ein bedeutender Aspekt des Projektes. #0:33:01-02#

m(6): Ok. #0:33:02#

M.U.: Man versucht nun im Detail bei gewissen Stoffen darüber Klarheit zu bekommen was es sie für Auswirkungen haben. Und dann möchte man das natürlich auch in regulatorischen Maßnahmen umsetzten. #0:33:02-18#

m(6): Also wir jetzt noch keine Ahnung? Reden wir jetzt von Reaktionen über..? #0:33.19- 21#

M.U.: Wir haben nicht keine Ahnung, aber wir haben in manchen Bereichen noch nicht genug Ahnung. #0:33.22-26#

m(6): Ok #0:33:26#

M.U.: In manchen Bereichen müssen wir eben noch alle Daten, die vorliegen, ausarbeiten, auswerten und schauen ob es gibt es tatsächlich einen Anstieg gibt. Gibt es tatsächlich einen Anstieg der Cholesterinwerte generell durch eine bestimmte Belastung durch den Stoff? (bejahendes Mhm im Hintergrund) Wenn das der Fall ist, dann wird der tolerierbare tägliche Aufnahmewert herabgesetzt und dann wird es sicher Maßnahmen geben. #0:33:26-55#

m(6): Mhm (erleuchtet)

m(8): Was ich mir wünschen würde ist, dass ein Ergebnis dieser internationalen Zusammenarbeit wirklich zu einem Regelwerk wird und dass nicht wieder so Geschichten passieren wie in Fukushima: Da ein kommt ein bisschen Radioaktivität heraus, das ist nicht so schlimm. Es ist nichts passiert nur leider sind ein paar Fische verstrahlt, das macht ja nichts, dann heben wir halt schnell die Toleranzgrenze an und schon ist das Ding wieder verkauf bar. Auf der anderen gibt es das Problem, das man für die Untersuchung der Schadstoffe oder deren Auswirkungen, Zeit braucht. Man gibt heutzutage sein Säugling oder ein Frühchen in das Spital, dort wird es mit Medikamenten versorgt. Bloß gibt es keine Medikamente für diese Gruppe. (zustimmendes ja im hintergrund-weibliche Person). Man weiß es nicht offiziell aber es könnte ja Auswirkungen davon geben Ein Aspirin ist für einen männlichen, 20-25 Jährigen ist halt ein Aspirin C. Es gibt Studien, die zeigen, dass das Ganze bei jungen Frauen, die die Pille nehmen, zu Herzinfarkten führen kann. Ich weiß nicht ob es schon offiziell gehandhabt wird oder das noch immer nur herum kriecht. Ich hab eine Studie gesehen, da ich eine Zeit lang bei einer Pharmafirma war, wo man auch solche Studien in die Hand bekommen hat. Das wäre irgendwie schön wenn so ein Programm als Nebenwirkung hat das Dinge offizieller werde. #0:33:58-34:43#

M.U.: Mhm (bejahend). Transparenter, meinen Sie jetzt? Also dass es quasi wenn es ein Ergebnis gibt das auch..#0:35:50#

m(8): Räuspern

w(8): (unterbrechend): Zugänglicher einfach. #0:35:52#

m(8): Zugänglicher einfach. Nicht transparent. #0:35:55#

w(8): (darüber) Zugänglich! #0:35:55#

m(8): Transparent ist das Fenster. Wenn das wirklich geputzt ist, dann rennen sie dagegen und sehen es nicht. Und deswegen wird der Begriff Transparenz so gerne verwendet. #0:35:56-36:04#

M.U.: mhm

S.B.: Aber Sie meinen, dass diese Initiative einen Beitrag dazu leisten kann, dass diese Studien zugänglicher werde? #0:36:06-08#

m(8): Richtig, das mehr Menschen anfangen zu lesen. Was ich zum Beispiel in hinter Kreisen sehe, also in Kreisen, die sich mit Bienen und in weiter folge mit Wildbienen und weiß Gott was alles beschäftigen, ist das es auch ein riesen, wie soll man das sagen, nicht ein Unwissen gibt, sondern ein Falsch-Wissen. Es werden Spunde publiziert! Ich weiß nicht wie sie eine Studie lesen. Ich lese eine Studie immer von hinten. #0:36:10-50#

w(8): Statistiken (unterbrechend) #0:36:50#

m(8): Literatur. nein! nicht Statistik, Statistik ist wurscht. Die schaue ich mir dann nachher an. Zuerst: was habe ich in den Literaturverzeichnissen drinnen stehen? Wenn Vorne groß drauf steht, dass Spar jetzt Produkte mit Mikro Plastik, raus schmeißt, aber nicht belegt was hinten nachkommt dann ist das ein Zeitungsartikel. Diese geistern durch die Foren, durch die sozialen Medien und werden von einem zum anderen weiter gereicht. Und, um zurück zur Imkerei zu kommen, auch in manchen Bienenzeitschriften herum gereicht. Der eine sagt das sich dieser Stoff in so und so vielen Tagen abbaut. Das stimmt unter Naturbedingungen, dort tut er das zwar, aber in der freien Natur mit Trockenheit ist das anders. 100 km weiter in einem Feuchtigkeit mit einem sauren Boden schaut es schon wieder ganz anders aus. Das auch Sachen wie diese vielleicht ein bisschen mehr in die Bevölkerung ausstrahlen könnten. #0:36:55-37:54#

S.B.: Was könnte jetzt diese Initiative dazu beitragen? #0:37:56-58#

m(8): Naja, wenn ich jetzt her gehe und die Daten in Wien bestimme und die Daten in Amsterdam, dann haben wir da ein bisschen eine Differenz. Ich war auch einmal in (Inraum-kremien-wort?), also ich weiß wie schwer es ist alleine einen Ringversuch zusammen zu bringen. Das Zusammenarbeiten gut hin zu kriegen ist schwer. Ich weiß nicht wie vielen Leuten noch der Glykol-Wein etwas sagt? (zustimmendes Mhm im Hintergrund von 2 weiblichen Personen). Also wenn damals ein Labor Glykol im Wein gefunden hat, hat das noch lange nicht geheißen, dass ein anderes Labor auch Glykol darin gefunden hat. Oder das eine hat so viel gefunden und das andere hat gesagt das ist noch unter der Toleranzgrenze. So etwas ist natürlich. Also da auch Wege zu finden, dass das über also im Ding(?) Bereich geht das ja schon oder ging, jetzt wird es eh wieder abgebaut und verweichlicht. Dass, das wieder mehr zum Tragen kommt. Das man Mechanismen entwickelt damit solche Regularien das nächste Mal leichter funktionieren. Also dass diese Mechanismen nicht unbedingt an dem einen Stoff festgehackt werden sondern wenn man sich das nächste Mal so etwas anschauen will man einfach hergehen kann und sagen : ,,Leute, da haben wir jetzt schon so einen Stapel Papier, da haben wir schon ein Verfahren, das muss jetzt nicht mit 20000 oder 20000/1 zertifiziert sein, sondern es funktioniert und das nächste Mal machen wir das genauso.‘‘ (Pause) Man wird ja träumen dürfen (Hintergrund Gelächter). #0:38.00-39:30#

S.B: ich glaube Sie wollten etwas sagen? #0:39:32#

w(?): Ok ganz, ganz simpel. Ich wüsste gerne, für mich selber und für keine Ahnung wen, vielleicht meine Kinder, wenn ich mal irgendwann einmal welche haben werde, was auf mich eine schädliche Wirkung hat und was nicht. Und ich bin kein Chemiker oder kein was auch immer und ich habe keine Ahnung, wie ich es verhindern kann dass ich diese Stoffe zu mir nehme, deshalb wüsste ich gerne was ich tun kann damit ich gesund bleibe oder auch wie ich gesund werde, jäh nach dem. Und auch das der Teil, über den ich keinen Überblick habe, für mich gemacht wird. Ich kann ja jetzt kein Doktorat in Chemie abschließen nur damit ich gesund leben kann. Das wäre irgendwie viel. Das heißt, ich hätte gerne, dass ich nicht in irgendwelchen komplizierten Inhaltsdingern nachsehen musssondern mich darauf verlassen kann, dass wenn ich etwas kaufe, das auch gesund ist. #0:39:35-40:34#

m(?): So etwas wie ein Hinweis. #0:40:36#

w(?): ja, ja sowas wär gut (lachen im Hintergrund) mein Gott. #0:40:38-40#

m(?): Naja...

(Lachen, allgemein)

m(?): Es ist zwar nicht gut, aber es funktioniert so halbwegs. #0:40:44#

w(?-redet darüber und dazwischen-im Hintergrund wird gelacht): Ich meine ich muss mich ja darauf verlassen können, dass andere Leute für mich die Arbeit machen, die ich selber nicht machen kann, weil ich mach schon meinen Teil auf einem anderen Gebiet leiste. Und da es quasi ihr Job wäre. Ich schätze ich würde ich mich gern darauf verlassen können, dass sie das für mich tun. #0:40:46-41:03#

M.U.: Wir bemühen uns! #0:41:05# (Lachen der Frau, die gerade zuvor gesprochen hat.)

S.B:: Und wie glauben Sie könnten sie dieses Wissen erlangt haben im Jahr 2022, wenn die Initiative abgeschlossen ist? Man weiß ja nicht, wie es jetzt dann weiter geht. Was ist da passiert, dass sie dieses Wissen nun haben? #0:41:07-18#

w(?): Bitte? #0:41:20#

S.B.: Was ist passiert, dass sie dieses Wissen 2022 auch haben, das sie gerne haben möchten? #0:41:23-24#

w(?): Naja, Sie sind einmal zu dem Wissen gekommen was überhaupt schädlich ist, denke ich einmal. Und wo das überall enthalten ist. (zustimmendes Mhm von S.B.) Dann müsste man damit anfangen mit Industrie und Politik zusammen zuarbeiten so, dass die Sachen, die schädlich sind, rausgenommen werden. Das fände ich sehr sinnvoll. So dass ich mich darauf verlassen kann, das diese Stoffe nicht mehr darin sind. #0:41:29-47#

Getuschel von einer männlichen und einer weiblichen Person gleichzeitig.

m: Das ist noch kein Ersatz. Da es vorher um Lesen und Informationsverteilung ging, möchte ich noch sagen, dass Youtube-Videos super sind um Ihre Publikation schön und frei herauszubringen. (zustimmende Ja's im Hintergrund) Youtube-Videos sind toll. Das war es schon. #0:41:52-42:03#

S.B.: Bitte. #0:42:04#

w(?): Ich möchte zuerst einmal etwas zu dem Thema sagen das sie vorheraufgebracht haben, und zwar wie man die Konsumenten zu etwas bringt, oder überhaupt die Bevölkerung zu etwas bringt. Wenn man sich anschaut, dass es vor 20 Jahren fast keine Biolebensmittel gegeben hat, muss man sagen in dem Bereich ist heute schon sehr viel passiert. Da wollte ich sagen, dass der Konsument schon Kraft hat um etwas zu verändern. (Pause) Zu den Ringversuchen wollte ich auch etwas sagen. Also es gibt die Ringversuche bei Lebensmitteln, da wird auch in ganz Europa geschaut wie viele Pestizide und so weiter in den Lebensmitteln sind. Natürlich funktionieren die Analysegeräte nicht gleich, aber ein Trend ist doch zu sehen, nicht? Also auch wenn es jetzt nicht exakte Daten sind so sehe ich doch etwas. Ich meine, mittlerweile wissen wir das spanisches Gemüse einfach stärker belastet ist als jenes bei uns. Und genauso sehe ich das auch hier, wenn man die Daten aufnimmt. Ich meine man weiß ja was die Stoffe in etwa verursachen, auch wenn man es wahrscheinlich noch nicht ganz genau weiß, was sie im menschlichen Körper machen. Ich nehme da immer das Beispiel eines großen Uhrwerkes, das unsere Bio-Chemie darstellt. Da dreht man an irgendeinem Rädchen und gleich rasselt alles ganz deppert. Ich glaube wir wissen da noch sehr wenig davon, aber deswegen gibt es ja so viele gescheite Leute. Daher finde ich, dass das wichtigste Datenerhebung ist und auch, dass diese mit dem was man schon weiß verglichen werden. Die Ergebnisse dieser Datenerhebung dann zu publizieren und öffentlich zu machen, dann kann die Bevölkerung auch den Druck machen, wie es ja auch schon in der Vergangenheit passiert ist, das diese Stoffe verboten werden. Es müssen Alternativstoffe gesucht werden. Ich meine das was wir mit unserer Welt aufführen ist ja wirklich ganz, ganz traurig. (Einwurf im Hintergrund: Grauslich!) #0:42:06-44:08#

w(?): Vielleicht können sie da (lacht) nichts machen, aber ich denke mir da immer, jetzt ist es so, dass herauskommt das die und die und sie Substanz sehr schädlich ist. Und dann gibt es ein paar Leute, die halt sehr viel verdienen, und sich deshalb können ob sie sich jetzt ein Ersatzprodukt, das aber mehr kostet, kaufen weil ihre Gesundheit es ihnen wert ist. Und dann gibt es Leute, die sich das einfach nicht leisten können. Also das ist ja bei Bio auch so. Viele Leute können es sich nicht leisten Biolebensmittel zu kaufen. Die kaufen dann die Anderen, wo die Stoffe eventuell noch drinnen sind. Ich finde da gehört auch eine Sozial-Geschichte dazu und würde mir wünschen, dass da irgendwie auch eine Kooperation herrscht. Vielleicht mit Löhnen oder was weiß ich was. So dass es für viele Menschen und nicht nur für irgendeine privilegierte Gruppe möglich ist sich Produkte zu kaufen, in denen keine schädlichen Stoffe drinnen sind. #0:44:10-45:13#

S.B.: Ja. Und wir sind jetzt in 2022, das Projekt ist abgeschlossen. Was hat das Projekt dazu beitragen können, glauben Sie?

w(?): Ja ich weiß eben nicht ob es möglich ist sich mit dem Sozial-Ministerium zusammen zuschließen. Umverteilung, oder was weiß ich was. (Gelächter) Oder das man Ersatzstoffe finde,t die vielleicht nicht schädlich sind, aber auch nicht teurer werden, oder so in der Art. #0:45.22-48#

m(?): Dazu will ich bemerken: Kann man überhaupt in dem kurzen Zeitraum schon feststellen welche Auswirkungen das haben könnte oder brauchen wir da Langzeitstudien? Normalerweise weiß man doch das wenn Medikamente neu auf den Markt kommen, zumindest zehn oder längere Jahre an ihnen getestet wurde, beginnend beim Tierversuch und dann mit Probandenversuchen und, und, und. (M.U,: Einwurf: Ja) Fünf Jahre sind für mich eine kurze Zeit. #0:45:52-46:18#

M.U.: Ja das stimmt schon, aber es gibt natürlich schon viel dazu. #0:46:20-21#

m(?): Das ist der Anfang. #0:46:22'

M.U.: Also wir haben zu den Stoffen, vor allem zu diesen prioritären Stoffen, die zuerst auf der Agenda stehen, auch schon Daten. Und die werden jetzt ausgewertet. Von den Phythalaten, die werden vielleicht mehreren Leuten ein Begriff sein, das sind diese Kunststoff-Weichmacher, wurden schon einige verboten, weil man definitiv weiß, dass sie eine schädliche Auswirkung haben. Im Rahmen des Projektes wird man nun versuchen sich die Ersatzstoffe anzuschauen. So wird gefragt ob die wirklich besser sind? Also ob es ähnliche schädliche Wirkungen gibt. Müssen wir auch bei ihnen auf etwas aufpassen? Wie schaut das damit aus? Also es gibt für Bisphenol A, das wurde hier auch viel diskutiert und untersucht, Ersatzstoffe? Bei denen hat man auch schon gewisse Hinweise und da wird sich dann vielleicht herausstellen das ein total Verbot sinnvoll wäre. Aber wie steht das mit diesen Ersatzstoffen, also zum Beispiel Bisphenol S und Bisphynol F. Da wird ein kleines bisschen an dem Molekül gedreht und wir haben eine Alternative. Bei der gilt es jetzt herauszufinden ob das wirklich eine gute Alternative ist oder man vielleicht radikaler denken muss? #0:46:23-47:46#

m(?): Ja also ich habe fünf Punkte. (Gelächter) Der erste Punkt ist: Ich hätte im Jahr 2022 gern eine allgemein verständliche Darstellung der Ergebnisse und dass sie diese auch entsprechend bekannt machen, damit diese nicht nur in scientific-communities und in den policy-communities bleiben. Der zweite Punkt ist: Es soll zuerst Ergebnisse darüber geben was die Stoffe tun. Das Dritte ist eine allgemein verständliche Handreichung für Konsumentinnen und Konsumenten, die darlegt wie man dann diese möglichen Belastungen vermeiden kann. Ein vierter Punkt ist das Aufzeigen von Feldern wo es einen politischen Bedarf gibt. Ich sage jetzt absichtlich nicht ,,politische Empfehlung‘‘, weil man sich das immer so ein bisschen aus den Fingern saugt. Also einfach nur zeigen, da ist ein Bereich, in dem brauchen wir Regulierung oder da braucht es vielleicht Deregulierung. Oder da muss man weiter untersuchen. Und das Fünfte ist, das es ein Human Bio Monitoring Netzwerk über ganz Europa geben sollte, und zwar dauerhaft, so dass man diese Untersuchungen langfristig fortfahren kann und auch Untersuchungen wiederholen kann. Das man sich nicht auf eine einmal gemachte Untersuchung verlässt, weil es können ja auch Irrtümer passieren. Das wären meine fünf Punkte.#0:47:47-49:33#

m(?): Man sollte vielleicht auch auf Basis dieser Ergebnisse wissen, wo es sich lohnt wirklich längere Zeit auch noch weiter nach zu schauen. Das heißt, dass man wieder neue Stoffproben spezifiziert, andere einfach weiter untersucht und letztendlich auch noch weitere Maßnahmen daraus ziehen kann. Das nicht einfach Schluss ist, nur damit es die EU gemacht hat und dabei kommt es nicht zu guten Vergleichen. Man könnte da ein bisschen besser vergleichen und das es einfach letztendlich langfristig irgendeinen Effekt hat. Nicht nur die 4 Jahre fertig, Schluss. #0:49:40-50:19#

w(Segel): Also was ich mir auch wünschen würde, wenn ich genau nachdenke (pause). Ich meine wir, in Europa, rotten die Umwelt ziemlich aus. Wir haben alle das wahnsinnige Bedürfnis ewig alt zu werden, also ein hohes Lebensalter zu erreichen, werden aber gleichzeitig immer jünger krank. Dabei muss irgendwann einmal auch Schluss sein mit den menschlichen Bedürfnissen. Man sagt:,, Ich will noch älter, noch agiler, noch unantastbarer und so weiter durch Stoffe werden.‘‘ Dabei sollte gesagt werden: ,,Aus! Diese Stoffe werden verboten und es gibt eben nun mal Regen in dieser Natur und deshalb musst du eben riskieren, dass du nass wirst. Punkt!‘‘. Es gibt auch Leder. Es gibt auch Dinge die lang halten als Produkt. Ich habe eine Lederjacke, die über 30 Jahre alt ist, die ist noch tadellos. Wenn ich die mit ganz normaler Schuhcreme eincreme hält sie. Ich meine es gibt so viele Stoffe, warum müssen wir ewig lang in Mikrofasern leben und unantastbar sein gegenüber der Umwelt? ich frage mich ob wir sowas wie Mikrofaser brauchen? Brauchen wir das eigentlich alles? Brauchen wir diesen hohen Lebensstandard? Ich meine wir leben wirklich im Paradies, hier in Europa. Warum nicht einfach mal radikal sagen: ,, Basta! Aus! Keine Ersatzstoffe! Weg damit, so wie mit Asbest.‘‘ #0:50:23-51:53#

m(?): Da passt dazu dass man ja auch eine gewisse Bequemlichkeit hat.

w(?)(Segel): Wir wollen alles. (M redet leise-sie laut darüber) WIR WOLLEN ALLES! #0:52:3#

m(?): Da ertappe ich mich selber. Ich gebe es zu, früher habe ich meine Schuhe immer brav mit der Schuhcreme geputzt und - #0:52:4-6#

w(Segel)- unterbricht und redet darüber: Ja und man musste spucken damit sich der Glanz entwickelt hat, ja. #=52:06-14#

m(?): Ich werde das aufgeben. Die Produkte aus dieser Veranstaltung (allgemeines lachen) (unverständlich: Verbote oder ähnliches) #053:16#

w(Segel): Es gibt Ledermäntel. Ja, Ledermäntel, Lederjacken. #0:52:20-21#

m(?) (gleichzeitig leiser): Was wollen Sie mit dieser Industrie? Dann kriegen Sie noch mit den 4-Pfoten kontakt. (lachen)

w(Segel): Nein, natürlich nicht. Aber es gibt so viele Stoffe. Ich meine, meine Großmutter die würde sich irrsinnig abhauen würde sie jetzt wieder auf die Welt kommen, nachdem sie 20 Jahre lang tot ist, die würde sagen: ,,Seid ihr alle wahnsinnig?‘‘ Weil es war normal, dass wenn ein Gewitter auf einem Berg in Vorarlberg kam, man den Stecker vom Fernseher rausgezogen hat, weil es gefährlich war das der Blitz einschlagt. Wir leben in diesem Sicherheitspunker, wo man meint, dass einen sowieso nichts passieren kann, dann passiert irgendwas und man wundert sich. (dahinter redet ein Mann leise, währenddessen) Wir haben den Zugang zur Natur verloren. #052:30-53:07#

S.B.: Und ihre Erwartungen wären dann? #0:53:13#

w(Segel):Das aufgezeigt wird, dass man nicht alles um Geld haben kann, und dass wir einfach nicht bereit sein sollten unsere Umwelt dafür zu opfern, dass wir zwei Stunden im Regen spazieren gehen können ohne nass zu werden. Das gibt es nicht. Das gibt es wenn man am Segelboot lebt, da wird man auch nass. Du wirst patsch nass. Entweder spuckt dich das Meer an oder du wirst von Oben patsch nass. Und da hilft dir keine Segeljacke, da hilft dir das einfach nicht, das liegt in unserer Natur als Mensch, dass wir nass werden können. Wir brauchen keine Mikrofaseranoraks in denen man im Schneegestöber dann irgendeine Bergtour macht und uns dann wundern, dass die Lawine kommt. Wir haben bei diesem Wetter da draußen nichts verloren, Punkt. (verneinendes mhmh im Hintergrund) Entschuldigung, wir brauchen da keine wasserabweisenden Stoffe. #0:53.14-54:07#

w(?): Es hat ja auch früher Ölzeug gegeben #0:54:08#09

w(Segel): Es hat ein Ölzeug gegeben, da hat man geschwitzt man hat gestunken. Ok. #0:54:07-12#

m(?): Jetzt mache ich mir doch glatt ein bisschen Gedankendarüber warum ich heute hierhergekommen bin, bei dem Wetter. (allgemeines Lachen) #0:54:18-19#

w(Segel): Und vor allem: Wie, na? Ich meine, früher hat man den Kindern dass sie nicht aus Zucker sind und einfach hinausgehen sollen, oder? #0:54:20-26#

w(Segel): Bequemlichkeit #0:54:32#

m(?): Ein Teil warum es die Stoffe, derentwegen es jetzt Monitoring gibt, ist nicht die Bequemlichkeit sondern unser Sicherheitsdenken. Das wird man vermutlich, ich beziehe mich jetzt wieder auf 2022, dann auch nicht abschaffen können. Allerdings Sicherheitsdenken auf eine andere Art. Versetzen wir uns jetzt 4 Jahre in die Zukunft. Darf ich 200 Jahre in die Vergangenheit gehen? #0:54:27-56#

S.B: Kurz aber nur (allgemeines lachen) #0:54:52-52#

m(?): Ja, ja ich beeile mich. (lachen im Hintergrund) wir drehen das ganz schnell. Da ist einer am Marktstand, der ein Arzneimittel verkauft hat und bis die Leute an diesem gestorben sind, war er schon so weit weg, dass ihm nichts mehr passiert ist. (zustimmendes ja im Hintergrund) Also hat man dann irgendwann beschlossen, dass man Medikamente braucht die sicher sind. Und da hat so eine Art Sicherheitsdenken begonnen. Das Reinheitsgebot beim deutschen Bier hat wenig mit der Reinheit zu tun, sondern es ging darum, dass das Getreide fürs das Brotbacken und nicht für das Bierbrauen genommen worden ist. Jetzt komme ich schon wieder schnell in das Jetzt zurück, jetzt bin ich schon wieder hier. Wir leben in einer Welt wo uns vieles verkauft wird was gar nicht so ist. #0:54:53-55:39#,

w(Segel): Wir leben in einer verkehrten Welt, mittlerweile. #0:55.40-42#

m(?): Und daher haben wir es auch mit diesen Stoffen zu tun. #0:55:42-44#

w(Segel): Wir leben in einer absolut verkehrten Welt, finde ich. #0:55:44-47#

m(?): Und einen Punkt würde ich gerne noch dazu nehmen. Und zwar dass diese Daten, die man erhoben hat, also die Stoffe, die wirklich von jedem gesehen werden. (unverständlich) Also dass sie wirklich bekannt gemacht werden, die Stoffe nach denen gesucht wurde, so dass der nächste kommen kann und sagen: naja diese könnten wir auch suchen. #0:55:47-56:09#

leise stimmen im Hintergrund.

m(Y): Ok, also ich hätte noch zwei Sachen und zwar ganz abgesehen von dieser Diskussion. Ich hätte eine, für mich, furchtbar wichtige Frage. Sie haben gemeint, dass sie den menschlichen Faktor berücksichtig haben, Sie meinten, sie fragen die Probanden nach ihrem Essensgewohnheiten. Wäre es taktisch nicht möglich, dass sie Tagebuch führen sollten? Oder gibt es irgendwelche Möglichkeiten dass der menschliche Faktor raus kommt? Das klingt jetzt blöd aber es ist schon eine Variable die sehr.. (verneinendes mm im hintergrund-weiblich) #0:56:12-37#

w(Segel): Das geht nicht #0:56:39#

M.U,: Das gibt es natürlich schon, aber das sind dann andere Studien. Das sind dann spezielle Studien, die man designen muss. W dann gesagt wird die Leute sollen dann so und so viele stunden nichts essen und dann schon, und dann den Morgenharn abgeben (Fragender wirft ein: genau) Das sind sehr spezifische Forschungsfragen, das ist sehr hilfreich bei gewissen Themen #0:56:40-58#

m(Y): Ok, laufen die auch parallel, oder nicht? Also die eine ist so eine mit Stichproben? #0:57:01-03#

M.U.: Genau, also hier geht es darum wie das geographisch ausschaut. Können wir für Europa sagen wie ist die Belastung in Europa gesamt und wie ist sie in den unterschiedlichen Regionen? Das andere sind Zeitverläufe, diese werden auch im Rahmen des Projektes untersucht. Gibt es Anstiege oder gibt es auch Abnahmen? Wie wirkt sich das aus wenn ein Stoff verboten wird? Oder sind die Verbote ausreichend oder muss man sie ausweiten? Also das wird auch untersucht. Und man versucht natürlich auch dann die gesundheitlichen Auswirkungen zu erfassen. Und die gesundheitlichen Daten mit den Human Bio Monitoring Daten zu verschneiden. Das ist auch ein Aspekt. Es ist wie gesagt, eine sehr große Geschichte, mit 28 Ländern und über 100 Institutionen. Es ist natürlich eine Herausforderung für das deutsche Umweltbundesamt, die das koordinieren. Die haben sich schon einiges vorgenommen. Es ist spannend. #0:57:02-58:04#

m(Y): Und noch eine Kleinigkeit. Aus gegebenen Anlass, bezüglich der Teilnahme der Probanden. Ich möchte mich für den Datenschutz aussprechen, der muss hochgehalten werden in jeglicher Art und Weise. Wir wissen nichts bezogen darauf wie das endet, aber wir hoffen einmal das trotzdem der Datenschutz eingehalten wird .#0:58:05-21#

M.U: Das ist ein ganz wichtiger Aspekt. Es gilt jetzt auch eine neue Datenschutzrichtlinie, von der EU, die auch sehr streng ist und wo sehr viel quasi zurückgespielt wird auf denjenigen der seine Daten. Der Patient ist Daten-Eigentümer, nicht mehr das Spital oder sonst wer, sondern wirklich er. Also er kann, und soll dann auch, entscheiden können was er zur Verfügung stellt. Und in diesem Fall wird es natürlich eine Einverständniserklärung geben, in der steht, dass man seine Daten in anonymisierter Form zur Verfügung stellt. Und natürlich werden die Daten geschützt. Aber ich meine Hacken ist natürlich generell ein Problem. #0:58:22-59:03#

m(Y): Nein, ich meinte mehr von der offiziellen Seite. #0:59:02-04#

M.U. Ja #0:59:06#

Wirres Gerede im Hintergrund. Gelachter. Ein Mann wiederholt offiziell. Unverständliches.

M.U.: Naja, ich sage nur dass es prinzipiell ein- #0:59:8-10#

S.B.: Sie haben vorher noch aufgezeigt? #0:59:10-11#

w(jung?): Ja. Naja. Nur ganz kurz zu dem was ich dazu meine. Ich fände es sehr schön wenn sich aus den Ergebnissen eine Diskussion entwickeln würde darüber wie wir diese Stoffe ersetzen können. Brauchen wir eine Innovation? Also müssen wir neue Stoffe entwickeln, die wir dann erst wieder testen müssen ob die jetzt wieder ok sind, oder vielleicht können wir einmal ein bisschen schauen was es sonst so gibt? Ich habe den Verdacht das ganz viele Sachen ersetzbar sind, und fast schon unnötiger weise ersetzt wurden eigentlich. Ist es in allen Bereichen überhaupt sinnvoll, dass sie ersetzbar sind? Wenn man sich das jetzt historisch anschaut. Das man vielleicht nicht immer in Innovationen denken muss, sondern vielleicht auch einmal.. #0:59:12-1:00:05#

w(neu-älert): Ja, wenn man nun sagt es ist 2022; dann wünsche ich mir, dass man diese Stoffe publiziert, und zwar für jedermann. Das man weiß was ist positiv und was ist negativ. Ist viel positiv? Ist viel negativ? Ich finde eine gewisse Eigenverantwortung gibt es auch, wo ich selber entscheide was ich will oder auch nicht. Aber zuerst muss man mal wissen was für Möglichkeiten es überhaupt gibt. Und die Sachen, die wirklich umweltschädlich sind, sollten verboten werden. Oder sind dann verboten. #1.00:06-1:00:42#

m(?): Ich wollte nur ganz kurz etwas zu Ihnen sagen, bezogen auf die unnötigen Dinge. Die Pille ist ja allseits bekannt und hat gewisse Nebenwirkungen, die man dadurch versucht hat zu verhindern dass man die Konzentration, die im Kreislauf herum irrt reduziert hat. Das heißt also dass das Hormon öfters verwendet und nicht sofort ausgeschieden wird. Das ist die Kehrseite von der Medaille. Also für die Frau ist es jetzt besser, es ist jetzt nicht mehr so schlimm mit den Krampfandern und so weiter. Die Kehrseite der Medaille ist das die Bakterien in den Klärbecken haben mit den schwer abbaubaren Hormonen ein Problem. Was passiert? Diese gehen jetzt ins Wasser hinaus und dort gibt es Fische, auch männliche, die haben mit den Resten von der Pille ein Problem. Also ist jetzt die Pille positiv, oder nicht? #1:01:43-01:54#

w(Segel): wir haben auch grundsätzlich mit den Anti-Epileptikern, die überall im Grundwasser drinnen sind, in vielen Staaten dieser Erde ein Problem. #1:02:01-02#

m(?): Also ich wollte das nur sagen, weil Sie das vorher angesprochen haben

w(J): Ja, das ist klar#1:02:04-#

m(?): Man sieht oft die Dinge nicht! #1:02:06-07#

m(Y): Ich denke nicht, dass es unnötige Entdeckungen gibt. Ich denke nur man sollte sich wirklich bei jedem Produkt überlegen: Wofür ist dieses Produkt da? Was soll es leisten und was für Risiken geht man ein? Das sollte eine bewusste Entscheidung sein. Ich meine für Outdoor-Sachen mag es vielleicht Sinn machen das ich sie nütze, wenn ich auf einen Berg steigen will, dann will ich nicht nass werden aber für andere, also für in die Schule gehen, in die Uni gehen, arbeiten gehen, oder was auch immer da brauche ich meinem Kind ja keine Jacke anziehen die minus dreißig Grad aushält. Hauptsache es reicht damit das Kind nicht krank wird. #1:02:10-45#

m(?): Ein Punkt noch, ein sechster. Es wäre schön wenn bei den Ergebnissen auch auf nicht intendierte Effekte, die sich ergeben haben, geachtet wird. #1:02:46-56#

M.U.: Entschuldigen Sie, das habe ich jetzt akustisch nicht verstanden. Nicht was für?#1:02:56-03:02#

m(?): (unterbrechend): Auf nicht intendierte Effekte, also die Antibabypille, die Sie erwähnt haben, die hat zum Beispiel den nicht intendierten Effekt, dass sich die Hormone aus die männlichen fische auswirken, also wo es dann sozusagen transsexuelle Fische gibt vielleicht, oder sowas. (lacht) #1:03:03-22#

m(?): Das hat aber sehr viele Jahre gedauert, also bis 2022 geht sich das nicht aus. #1:03:26-28#

m(?) Nein, nein. #1:03:30#

w(A): Und hat das auch Auswirkungen auf Männer, wenn man das Wasser nimmt? Oder nur auf die Fische? #0:03:34-40#

M.U.: Nein das sind die Umweltorganismen. Also durch die Arzneimittel, die die Menschen ausscheiden, werden hauptsächlich die Umweltorganismen betroffen. Das ist auch bei Schmerzmitteln so. #1:03:41-51#

w(A): Menschen trinken ja das Wasser oder? #1:03:51-52#

M.U.: Der Mensch trinkt das Wasser, aber die Konzentrationen sind so gering dass es- #1:03:53-57#

w(A).(unterbrechend): Das wollte ich wissen. Das ein keine Auswirkungen hat. #1:03:57-59#

m(?): Also so wird sozusagen die Homöopathie ad-absurdum geführt. #1:04:00-03#

M.U: Also.

m(älter): Das weiß man ja dann auch wieder erst in 100 Jahren, in 200 Jahren. Jetzt ist das angehäuft. #1:04:08-12#

w(älter): Man wird doch homöopathisch beeinflusst und es hat Auswirkungen. #1:04:12-15#

m(Y): Ja, sicher #1:04:14#

m(?): Es gibt ja dann auch die anderen Sachen. Ich meine Contergan, das sagt vielen Leuten noch etwas. (allgemeine zustimmende Geräusche) Contergan ist noch immer ein notwendiges Medikament. #1:04:14-30#

S.B.: Bitte #1:04:32#

w(?): Vielleicht noch etwas ganz banales zu diesen Perfluor-Substanzen. Ich habe letztens Jahr so eine tolle Jacke gekauft. Dabei wurde ich nur über die Vorteile aufgeklärt. Und vielleicht, wenn ich mich jetzt vorversetzte ins Jahr 2022, wäre es so dass dann dieser motivierte Verkäufer mir die Vorteile aufzählt aber auch dass dieses tolle neue Material mit diesem und jenen imprägniert ist. Das könnte zu Ausschlägen führen, oder was auch sonst. Das mir auch die Nachteile aufgezeigt werden. Und dass ich dann, als mündiger Konsument, entscheide ob ich die Vorteile will und dafür die Nachteile auch in Kauf nehme, oder ob er sich seine Jacke behalten soll und ich kauf eine andere und ich friere oder ich werde nass, ganz gleich. #1:04:33-05:13#

w(?): Praktisch wie beim Medikament. Da stehen ja immer so viele Nebenwirkungen drauf, dass man es ja eigentlich kein einziges Mal nehmen dürfte. #01:05:14#

w(? unterbrechend): Ich fürchte das Problem mit der Mündigkeit ist das der Mensch dann immer ad-absurdum geführt wird, weil wir können das nicht alles wissen. Ich glaube auch, dass das von weiter Oben gesteuert werden müsste. Ich kann mich jetzt nicht entscheiden, ob ich jetzt lieber eine Baumwolljacke, die mit irgendeinem Kunstharz bedruckt ist, oder so eine Outdoor-Jacke. Ich glaube, dass der Einzelne a-la-long nicht alles entscheiden kann, weil es eben alles ein bisschen weiter nach hinten geht oder nach vorgeht. Ich glaube, dass man das einfach in einer höheren Ebene entscheiden muss. Dabei geht es halt um Datensammlung und um zu schauen was die Stoffe machen. Ich meine, wir wissen ja schon was die Stoffe machen. Ich glaube, dass es wirklich ein Aufschrei sein wird wenn man erfahrt was wir alles in uns schon drinnen haben weil bis jetzt glauben wir ja, es geht so alles an uns vorbei, wir leben in der Blase. #1:05:14-06:29#

w(segel): Wir leben in super Luxus, irgendwie 60facher Überschuss. #1:06:29-36#

w(älter?unterbrechend): Aber es muss von Oben gesteuert werden und da gibt es ja schon ganz viele Beispiele in der Vergangenheit, die jetzt nicht mehr da sind, in Europa zumindest. Ich meine auf der Welt, glaube ich, wird DDT noch immer verwendet. #1:6:36-50#

w(J): Ich hätte noch ganz kurz was. Ich denke es ist den Leuten schon zumutbar, dass man sie informiert. Ich treffe gerne eine informierte Entscheidung, was Risiken betrifft, und ich traue mir es auch zu das zu entscheiden. Ich glaube das kann man auf ein Maß runterbrechen so dass es einem Durchschnittsmenschen möglich ist, eine Kaufentscheidung zu treffen#1:6:50-07:14#

w(?): Glaube ich nicht. #1:07:14#

w(I): Naja es wäre wünschenswert. Also das würde ich mir wünschen. #1:07:15-19#

m(Y): Also ich unterstütze die Kollegin da sehr. Ich hätte noch einen Wunsch was 2021 betrifft?

w(?): 22, kann auch schon 21 sein. #1:07:28-29#

Lachen im Hintergrund.

m(Y): Natürlich leidet da jetzt mein Techniker-Herz, aber ich glaube auch dass bei der Produktion Schadstoffe ausgestoßen werden. Daher sollte auch hier angeschaut wird welche Probleme sind (unverständlich). Das die auch damit anfangen erstmal die Industrie darauf zu bilden. Und auch Förderungen anzubieten, dass die Industrie etwas dagegen unternimmt. Das heißt auch technische Innovationen zu fördern. Natürlich werden wir nicht von 0 auf 100 alle Stoffe wegkriegen, das wird nicht möglich sein. Das heißt, dass man dann auch darauf schaut mit technischen Innovationen Eindämmungen zu machen. Ich habe nie gesagt (unverständlich) (lachen im Hintergrund). Ich habe mich zurück gehalten #1.07:34-08:20#

S.B.: Gut, also 2022. Wir haben jetzt einige Dinge gehört, von denen Sie sich erwarten, dass sie dann sein sollen. Ich habe für mich noch zwei Unterpunkte, die von Ihnen schon angesprochen worden sind, ich möchte sie trotzdem noch einmal explizit ansprechen. Vielleicht kommen da noch Assoziationen. Welche politischen Maßnahmen sind 2022 gesetzt, welche erwarten Sie sich? Und das zweite ist; wie ist 2022 das Bewusstsein der Bevölkerung zu diesem Thema und wie ist man da hingekommen?-Bitte #1:08:25-09:11#

w(segel): Als Werbekauffrau, da bin ich in der Grundausbildung, befürchte ich, dass der Wissensstand der Bevölkerung zunehmend schlechter wird und immer mehr abnehmen wird. Warum? Weil die Summe der Botschaften immer höher wird. Man sieht das auch an diversen Werbebudgets, wie viel in gewisse Werbung investiert werden muss um auch einen gewissen Output am Markt zu kriegen. Ich glaube wir brauchen 2022 ein Fach in der schule, das heißt ,,Wie lebt man?'' so blöd das auch klingt. Weil teilweise sind die Eltern nicht mehr in der Lage ihre Kinder vernünftig, oder auch nur mit den Basisinformationen, zu versorgen. Weil die Eltern einfach vielem hinterherlaufen und sich nur fragen was möchte ich haben und wie krieg ich es. Es gibt einfach ein gewisses Basiswissen nicht mehr, das immer mehr verloren geht. Und deswegen brauchen wir ein Schulfach, für 14 Jährige, das einen Lebensleitfaden beinhaltet und Fragen beantwortet. Wie schaut gesunde Ernährung aus, wie schaut eine gesunde Bewegung aus, welche sind die Risikofaktoren, wohin gehst du wenn du das und das brauchst? Und so weiter. Ich habe von einer Lehrerin, im 21 Bezirk, gehört dass sie von den Eltern verlangt hat, dass für die gesunde Jause Obst mitgebracht wird. Und jemand hat ihrer Tochter irgendwelche Erdbeer-Zuckerl mit gegeben, mit keinen Erdbeeren. Und das war bitte ein Kind ohne Migrationshintergrund. Ich muss sagen, ich geniere mich teilweise für den Wissensstand der Österreicher, auch im Bereich der Ernährung. Auch wenn man ihnen etwas sagt nehmen es nicht zur Kenntnis. Sie wollen es nicht, weil eben ein normaler Apfel, der nicht behandelt ist und vielleicht ein bisschen runzeliger ist, nicht so schön ist wie der Pink Lady vom Billa. (Hintergrund versuch zu unterbrechen: haben sie) sie wollen es nicht mehr. #1:09:12-11:18#

S.B: Haben sie schon eine Idee? Also eine Idee, die Sie jetzt gebracht haben ist das Schulfach #1:11:18-20#

w(segel): (unterbrechend): Der lebensunterreicht. Also du sollst kein konto-überziehen. Du sollst deine Geschäftsbedingungen lesen, wenn du online unterwegs bist, weil da kann irgendwas passieren. Wir brauchen ein grundlegendes Fach, das sich Lebensunterricht nennt. #1:11:20-39#

S.B:: Mhm

W(J): Das sollte doch die Schule sein, allgemein #1:11:40-42#

w(segel): unterbrechend: Das sollte an der Schule sein. Da sollte dabei sein: Wie ernährst du dich? Angefangen von was sind die Grundzüge einer gesunden Ernährung, wie sind die Grundzüge, wie kommst du auf eigene Beine? Wo kannst du dir Hilfe holen? weil manche Jugendlichen stehen da und wissen nichts und sind arm. #1:11:42-12:00#

m/w gleichzeitig: Na wissen tun sie es schon. #1:12:02-05#

w(weiter): Wenn man nämlich einen Test, darüber macht gesunde Ernährung ausschauen soll, macht dann steht da Gemüse und so weiter. Und dann ist die zweite Frage, die lautet: was ist dein Lieblingsessen? Und dort steht Pizza und Hamburger. Ich meine sie wissen aber.- #1:12:06-15#

w(segel-unterbrechend): Ja aber das hat ja keinen Sinn. Ich meine ich bin Wellness-Trainerin. (Lachen im Hintergrund) Ich habe Körbe von Obst zuhause stehen und das wird nicht gegessen, auch bei mir in der Familie nicht, ich könnte mich aufregen. Aber wenn man hergeht und das als kleine Obstsalat-Portion portioniert isst jeder auf einmal Obstsalat. Jeder. #1:12:15-35#

w(?): Das weiß man, das wissen Sie auch. #1:12:37#

m(unterbrechend?): Das ist die Bequemlichkeit des einzelnen. #1:12:37-40#

w(segel): Wir leben auf dem Niveau und dabei haben wir die Basis verloren kommt mir vor, oder? #1:12:40-45#

m(?): Ich habe das gesunde Essen in der Volksschule meines Sohnes kennengelernt. Da hat man gesagt, dass auf Schnitzel und Co verzichtet wird und es mehr Pute gibt. (Lachen im Hintergrund) Hintergrund, ist der das an der schule zirka 70 Prozent der Kinder kein Schwein essen, aus der Familie heraus. Ob sie es eh essen täten weiß man nicht aber es wäre zu aufwendig wenn die Kinder entscheiden können ob sie das essen das oder das. Und für die gesunde Vormittagsjause gibt es dann einen Putenschinken, beziehungsweise einen Putenaufstrich. Jetzt sind hier lauter Lebensmittelchemiker her innen. #1:12:47-1:13:10#

w8segle-unterbrochen: wir brauchen gar kein #1:13:10#

m(?): Was ist in einem putenaufstrich drinnen? Ungefähr 60 Prozent Schwein. Ich meine die Ironie überholt die Politik selber. #1:13:27-32#

w(Segel): Ja. #1:13:28#

m(?): So und jetzt soll die Politik, oder der Stadtschuldrat, ein Fach für Lebenshilfe machen? Das wäre auch schon ein Wunsch. Das würde ich auch schon 2022 oder 21 nehmen, dass die Politik da ein bisschen mehr dazu gelernt hat. Weil das ist keine Seltenheit (Mann währenddessen: die Politik müsste früher reagieren) das können sie fast in jeder schule haben. In Deutschland dürfen sie in den Kindergarten, dort heißt er ja Kita, keine selbstgebackenen Sachen mitnehmen (segel(w)währenddessen: Auch bei uns nicht, auch bei uns nicht!) (andere Frau ebenso: Bei uns auch nicht mehr!): das ist eh schon flächendeckend, bei uns glaub ich geht es in manchen noch. #1:13:34-14:06#

w(segel): Nein, sie dürfen in den Kindergarten keinen Kuchen mitbringen. #1:14:08-10#

m(?): Weil da sind ja die bösen Bakterien drinnen. #1:14:12-13#

m(?): Ja #1:14:13#

w(segel) Das geht aber bei den Ärzten los. Es geht schon los wenn man ein Baby hat. Wenn ein Kind einen kleinen Infekt hat wird der Arzt mir ein Medikament empfehlen. Nur damit man den Arzt nicht klagen kann muss der Arzt, nämlich ein Medikament empfehlen anstatt zu sagen man solle eine Woche zuhause bleiben und sich um das Kind kümmern. Es geht in dieser Gesellschaft in erster Linie um Risiko Minimierung. Dabei dauert es mit Tabletten eine Woche und ohne Tabletten dauert es sieben Tage. Wir sind absolut abnormal. #1:14:13-53#

S.B: Ich würde jetzt gern noch einmal zum Thema zurück. Es ist natürlich sehr weitgreifend das Thema. #1:14:53-58#

W(segel): Eine Liste an Gefahren #1:14:58-59#

S.B: Da kommt noch sozusagen eine Liste an Gefahrenstoffen. Ich schaue jetzt noch einmal in diese Runde. Auf dieser Seite sind jetzt zu diesen Zukunftserwartungen an das Projekt doch weniger Argumente gekommen. Haben Sie da noch irgendwie Vorstellungen oder Wünsche an das was 2022 wäre? #1.15:00-21#

m(A): In der Zukunft, in quasi 3 Jahren, kann man ohne Politik nichts schaffen, eigentlich. Övp oder Fpö müssen dieses Programm, dieses Projekt, auch in ihr Parteiprogramm aufnehmen. Es ist sehr wichtig und einige Gesetze sollten kommen. Sowohl für die Industrie,, egal ob Lebensmittel- oder Pharmaindustrie oder so, also auch für die Konsumenten. Weil die Konsumenten, die Menschen, wissen schon ob diese Stoffe für die menschliche Gesundheit schädlich sind. Aber wir wissen nicht genau welche Stoffe von welchen Produkten kommen. So wie bei den Allergenen, aufgrund von dem Allergen-Gesetz können wir jetzt wissen welche Allergene von welchem Lebensmittel kommen. Aber über alle anderen Produkte wissen wir gar nichts. Es sollte eine Klassifizierung geben damit wir durch diese Klassifizierung wissen welche Stoffe von welchen Produkten kommen können. Ich glaube das ist das wichtigste Thema. #1:15:23-16:58#

S.B.: zustimmendes Mhm #1:17:00#

m(A): Wir müssen natürlich, durch Schulungen, durch soziale Medien, das der gesamten Bevölkerung irgendwie vermitteln. Diese Information weiterleiten. #1:17:00-15#

S.B: Bitte #1:17:22#

w(ält): Ich glaube nur ,dass das sehr wohl über die Politik geregelt gehört. Aber nicht über die Nationale, sondern über die EU, weil es weiter gestreut ist. Weil jedes Land kann sagen: das nehmen wir wieder zurück. (zustimmendes mhm im Hintergrund) Wenn das über die EU läuft dann ist das nicht mehr so. Nur wird es sich wahrscheinlich bis 22 nicht ausgehen, dass da alle mitmachen. Aber das wäre der Wunsch. #1:17: 23-48#

m(?): Wenn man da aber so sieht, wenn man mal im nationalen Bereich ein Ergebnis feststellt wird das man festmachen kann #1:17:50-56#

w(ält): Ja, aber das kann man nicht festmachen. Weil nach dem heben sie es wieder auf. #1:17.56-58#

m(?): Nein, nein aber die haben es einmal für Österreich, für das Teilgebiet. ##1:17:58-18:04‘

w(ält): Ja aber dann nehmen sie es wieder zurück weil irgendeine neue Wahl kommt. #1:18:04-05#

Lachen.

S.B: Noch einmal kurz. #1:18:07#

m(?): Dann muss ich aber wirklich eine Ruhe geben. (Hintergrund lachen) Ich hätte als Idealist schon folgendes gedacht: Ich bin ein (unverständlich) Europäer und denke das, das dann in Brüssel vielleicht gehört findet. Nur das Problem in Brüssel ist ein ganz ein anderes. Dort sitzen jetzt Leute die dort hingeschickt werden weil man sie in den Nationalstaaten nicht brauchen kann. Und es kann sein dass die dann dort nicht erkennen was hier vielleicht wertvolles geschaffen wurde, weil sie dort eben zu dumm sind, das ist das Problem. Das liegt aber auf einer anderen Ebene und hat mit unserem Projekt hier nichts zu tun. Aber es ist so, ich weiß es aus Erfahrung. #1:18:07-40#

M.U: Na also, nur ganz kurz dazu, natürlich gibt es viele solche Leute auch, aber in den Institutionen, also in der ECHA; EFSA und der Europäischen Kommission, sind die Anforderungen schon sehr hoch und auch das Fachwissen teilweise sehr hoch. Und die Dinge die da quasi beschlossen werden die sind schon fundiert. Worauf ich schon aber hinweisen muss, ist das es natürlich auch viel Lobbyismus gibt und das natürlich die Interessen der Industrie auch ein Gewicht haben und einbezogen werden müssen. #1:18:40-19:19#

m(?): Ich darf nur relativieren, meine Erfahrungen stammen aus einem ganz anderen Gebiet, einen ganz anderen Forum. Daher kann ich nicht beurteilen wie es in den anderen Gremien ist. die Gefahr besteht. #1:19:20-30#

m(?): Also ich wieder hole mich, wichtig wäre mir dieses Human Bio Monitoring Netzwerk und dass es dauerhaft besteht, sozusagen als politische Maßnahme. Wenn erwiesen ist, dass ein bestimmter Stoff schädigt, dann sollte es Verbote geben, wenn möglich. Wenn diese Stoffe nicht so einfach ersetzt werden können, dass kann es ja auch geben, dann erwarte ich mir schon eine breite Information in Funk, Fernsehen, sozialen Medien und Zeitungen. Allgemein verständlich wie man damit umgeht, worauf man achten muss und wie man Schädigungen möglichst vermeidet. Sie haben zuerst die Efsa erwähnt. Ich stehe ja der Efsa ein bisschen skeptisch gegenüber. Ich denke mir hier ist es wichtig dann auch darzustellen: Wie hat sich die Industrie an diesen politischen Maßnahmen beteiligt? Wie floss das ein? Weil normaler weise gibt es irgendwelche Steak-holder-Konferenzen, man weiß dass die dort sitzen, aber wie diese Entscheidungen getroffen werden, was da einfließt, das wissen die, die daran teilgenommen haben oft selbst nicht, außer diejenigen die es durchgesetzt haben. Also da ein bisschen mehr Legitimität in diesen Entscheidungsprozess hinein zu bekommen, gerade bei einen so sensiblen Thema wäre was Wichtiges. Vielleicht schafft das Österreich. Aber wenn Deutschland das koordiniert, dann könnte es vielleicht klappen, trotz der starken deutschen chemischen Industrie. (lachen im Hintergrund) Ok. gut. das wäre mein Beitrag. #1:19:31-21:33#

w(J): Ich denke es ist schon wichtig, dass die Leute informiert werden und das die Leute das wissen. Andererseits denke ich, dass es absolut nicht reicht. Es wird zwar immer gesagt Konsumenten haben so einen super Einfluss. Aber ich habe mich nie dafür entschieden, dass irgendwelche- keine Ahnung wie das nochmal heiß, in meiner Jacke drin sind. Da bin ich als Konsument jetzt nicht so dass ich sage: Ja ich bin Konsument, ich weiß das jetzt und sobald ich weiß dass es schlecht ist entscheide ich mich dagegen und kauf was anderes. Ich sehe im Moment nicht dass das so funktioniert. #1:21:35-22:09#

S.B: Das hier Information fehlt? #1:22:09#

w(J): Nein. Natürlich weiß ich gern was in meiner Jacke drinnen ist, ich denke aber das reicht nicht. Es ist nicht eine bewusste Entscheidung. Wie soll ich sagen? Ich habe mich nicht dafür entschieden, dass es drinnen ist und die Information, das es drinnen ist wird jetzt nicht dazu reichen, dass ich mich dagegen entscheide. Das liegt ja nicht an mir, das sehe ich in der reellen Welt. Da läuft das irgendwie anders. Die Entscheidung liegt ja bei anderen Leuten. Ja, schon das wollte ich noch mal gesagt haben, nur die Leute zu informieren ist unzureichend. #1:22:10-50#

w(ält): Aber ich muss mich ja auch darüber informieren was schlecht ist. #1:22:50-53#

w(J): Natürlich #1:22.55#

w(ält): Wenn ich nur weiß was drin ist, sagt mir das ja noch nichts #1.22:55-58#

w(J): Ja, ja, natürlich. Beides ist wichtig. Ich finde, die Leute sollten wissen was das für Substanzen sind und dass sie schädlich sind und so weiter. Ich wollte nur ganz kurz festhalten ich denke nicht dass es reicht, weil ich denke nicht das Konsumenten daran viel ändern können. Natürlich entscheidet sich niemand im Supermarkt so dass er sich denkt: So jetzt kaufe ich ungesunde Lebensmittel. Sie kaufen die Lebensmittel obwohl sie ungesund sind. #1:22:59-23:22#

w(segel): Genau #1:23:22-#

w(ält) weil es ihnen schmeckt #1:23:24-25#

w(J): Weil sie ihnen schmecken ja. Also das Ding ist kein Konsument kauft etwas weil es ihm schadet, sie kaufen es trotzdem. Und das ist das was interessant ist. Wo relativieren die Leute da? Wo sagen sie; dass ist es mir aber wert? #1:23:25-45#

w(ält): (unverständlich)

m(Y): Also das ist jetzt nichts bezüglich zu dem. Mir ist noch durch sie etwas eingefallen weil Sie besonders von Lobbys geredet haben. Da wollte ich noch folgendes sagen. Auch Industrie-Unternehmer sind Menschen. Einer sitzt hier (lachen) und ist per Definition ein Unternehmer. Da muss ich sagen sie könnten ja auch Projekte entgegenziehen, mit denen Sie besonders Unternehmer ansprechen, die diese Produkte herstellen. Sie können ja auch Aufklärungskampagnen in der Industrie führen. Weil ich glaube, dass die Meisten schon auch bereit zu Änderungen sind, wenn Sie ihnen sagen, dass würde so viel mehr kosten oder weniger, je nachdem, wie sie es schaffen. Und was dann passieren würde. Ich glaube, dass man auch den direkten Kontakt mit der Industrie selbst suchen sollte. #1:23:50-24:40#

M.U: Wie gesagt, wir haben dieses Steakholder-Forum, das wir als österreichisches Umwelt Bundesamt organisieren. Da ist drinnen: die europäische chemische Industrie, die cfic(??), dann auch die Metallindustrie, Vertretungen von der EU. Wen haben wir noch drinnen? Also wir haben insgesamt vier Industrievertreter, aber auch Arbeitnehmer, Konsumenten, Patienten und dann halt auch die NGOs, also die Umwelt- und Gesundheit-NGOs. Und da wird auch diskutiert und gefragt: Was habt ihr für Erwartungen? Was wollt ihr von dem Projekt? Und natürlich: die Industrie, das sagt sich leicht, aber es ist nicht eine Industrie, sondern es sind hier auch sehr viele verschiedene Ebenen zu berücksichtigen und Menschen und Interessen. Und natürlich ist es auch da wahrscheinlich so dass die wenigsten von vornhinein sagen wir machen das weil es schädlich ist sondern es wird in den meisten Fällen so sein das es andere Gründe für ihr Handeln gibt, aber eben diese Schäden auch da sind. (lachen im Hintergrund) Nicht gut? (lachen) also in Kauf genommen werden. #1:24:42-26-13#

m(äler): Ein Beispiel: wenn ich im Supermarkt zwei Käse Sorten anschaue, stehe ich da vor dem Regal und dreh sie um. Dann steht hinten alles Mögliche drauf. Als Konsument weiß ich vielleicht was E5 heißt, alles andere wird ja nie beworben, im negativen Sinn, zur Information der Konsumenten. Und dass ist das, was ich gemeint habe, wenn wir die Information bekommen, im Sinne einer Werbung, aber in der anderen Art der Werbung, und zwar nicht kauft das, sondern diese Stoffe sind drinnen und das kann schlecht sein. So das man dann aufmerksamer wird. Und ich selber werde also oft gefragt warum schauen Sie sich das da hinten an? Das passiert mir immer wieder beim Einkaufen.#1:26:13-27:02#

S.B: Bitte #1:27:5#

W(segel): Ich habe eigentlich aufgehört in meinem Ur-Job in der PR zu arbeiten, nachdem ich mehr oder weniger gesehen habe was meine Kollegen da in Brüssel aufführen. Und ich muss sagen, ganz Europa ist eine extreme Lobbying-getriebene Geschichte. Was mich ein bisschen stört ist das immer Ängste geschürt werden. Es ist für uns, in unseren Breiten, eigentlich ganz normal, dass man einen Cholesterinspiegel von 300 hat. Oder es war zumindest normal, bis man dann durch den Quotienten HDL-LDL, diesen anders festgesetzt hat und auf einmal hieß es Cholesterin-Quotient muss mit 200 bestimmt werden und alles was drüber ist, ist zu hohes Cholesterin. Mehr oder weniger ist diese Grenzwert Verschiebung von Becel gemacht worden. Von der Firma Puna, über Lobbyisten. Ich finde einfach das der Konsument sein eigenes Köpfchen wieder einsetzten muss. Ich sehe nicht ein, daas wir auf der einen Seite die Landwirtschaft mit EU-Förderungen unterstützen und Butterberge produzieren, und auf der anderen Seite dann die Industrie, und ich spreche bitte nicht gegen die Industrie, Palmöl hernimmt und in die Margarine rein gibt und wir lassen die Butter einfach liegen und sagen Becel ist wichtiger weil das hilft uns den Cholesterinspiegel gering zu halten. Also man muss die Dinge wieder in den richtigen Zusammenhang rücken und einfach ein gewisses Maß festlegen. Was sagt der HDL und der LDL Quotient aus? Es ist vollkommenegal ob du 200 oder 300 hast. Und deswegen glaube ich, und ich beharre in der Runde darauf, dass wir eine ganz normale Marke brauchen, wo das normale Leben auch drinnen steht. Also eine Liste an Stoffen, aufgeschrieben ist, in der sozusagen steht welche Auswirkungen die haben. Dazu brauchen wir eine öffentlich zugängliche Datenbank, wo man sich einfach mehr informieren kann. Was passiert? Was ist welcher Stoff? Was macht das (unverständlich)mit gewissen Chemikalien? Empowerment! #1:27:6-29.38#

M.U: Also auf der europäischen gibt es da sicher viel Nachholbedarf, auch von unserer Seite. Wir versuchen immer wieder unsere Studien an die Öffentlichkeit zu bringen und auch Folder zu machen und auch Informationsmaterial. Aber natürlich muss das dann auch abgefragt werden oder die Kanäle -#1:29:40-30:00#

W(segel)-unterbrechend: Es muss auch richtig angewendet werden, ohne Panik zu produzieren, ja. #1:30:00-03#

M.U.: Die Kanäle gehören sicher da noch intensiviert oder verbessert. Was haben sie noch mal gesagt? #1:30:03-05#

w(segel): Ja, diese Lebensmappe, wo man den Normalzustand mehr oder weniger.. #1:30:05-12#

M.U: Ja da bin ich ein bisschen skeptisch. Da lege ich jetzt meinen Hut als Toxikologin ab und nehme meinen Mutter-Hut. Also wenn man irgendwie in derartiger weiße Aufklärung machen will, dann muss man viel früher anfangen. Bei den Jugendlichen stoßt man dann eher auf nicht so wirklich offene Ohren, könnte ich mir vorstellen, also das hielte ich für verspätet #1:30:14-48#

W(segel): Früher hat es doch dieses Buch gegeben, das man bekommen hat wenn man 18 wurde. Das wurde doch vom Bürgermeister hergegeben. Sowas stelle ich mir vor, quasi als Lebensmappe wo alles drinnen steht. Was ist wenn du finanzielle Probleme hast? Was ist wenn deine Eltern Probleme haben? Was ist wenn du auf einmal vor geschlossenen Türen stehst? Wo sind die Notruf Nummern? Und, und, und. Ja so irgendwas das sie - bitte? #1:30:47-31:16#

W(J): Ich frage mich aber wer dann die Norm entscheidet? Man kann ja nicht jedem Menschen ein Leben aufdrücken mit einer Lebensnorm. #1:31:16-36#

w(segel):( unterbrechend) Die Grundsachen, das ist schon normal. Das grund-normale. Also was machst du wenn du kein Geld hast? An wen? Wo sind die offiziellen Ämter? Wir leben in einem Staat wo uns Netto ungefähr 20 Prozent, das muss man sich auf der Zunge zergehen lassen, 20 Prozent unserer tatsächlichen Kosten überbleiben. Der Rest sind Steuern und Gebühren und geht an den Staat. Deswegen sind wir auch so super organisiert in dem Land und wissen so viel, nur wir nützen die Infrastruktur zu wenig effizient um ein positives leben zu haben. Ja wir sudern, dabei haben wir es. #1:31:35-32:27#

w(J): Können sie nochmal vorlesen was eigentlich die Sachen waren, über die wir reden sollten? #1:32:28-29#

S.B.: Ja, (Gelächter im Hintergrund). Ich wollte jetzt gerade noch einmal zurück gehen und noch eine Frage stellen bevor wir eine kurze Pause machen und dann auch lüften. Sie noch einmal abholen und fragen ob es von Ihrer Seite noch Vorstellungen, Wünsche, Erwartungen dazu gibt was in 2022 sein sollte wenn die Initiative abgeschlossen worden ist? Was durch diese Initiative erreicht worden ist? Wir haben jetzt diese Parameter: politische Maßnahmen, Bewusstsein in der Bevölkerung aber auch Dinge die darüber hinausgehen. Also ich würde jetzt noch einmal so in die Runde schauen ob es dazu jetzt noch etwas gib. #1:32:35-33:08#

W(J): Was dann? #1:33:08-11#

S.B: Also wir haben 2022, die Human Bio Monitoring Initiative ist abgeschlossen. Was sind ihre Erwartungen und Wünsche, was dann erreicht wurde. #1:33.12-18#

W(J): Ich würde gerne Sachen kaufen, die nicht mehr gefährlich für mich sind #1:33:20-26#

allgemeines Gelächter.

S.B: Und wie schaffen wir das?

allgemeines Gelächter.

M.U: Man muss natürlich auch sehen, dass der Zusammenhang da ein bisschen auseinander geht. Die Jacke, die ich jetzt kaufe und die ich anziehe, die ist gut für mich weil ich jetzt am Berg nicht friere. Tatsache ist, dass der Stoff durch die Produktion, und selbst, schädlich ist aber es eben erst dann die Umweltbelastung ist, die mich dann wieder schädigen kann. Und dass ist gar nicht so einfach so zu kommunizieren. Also das muss auch richtig kommuniziert werden. Und dann müssen auch die Menschen sagen, dass sie das eigentlich nicht wollen. Dann tut sich die Politik auch leichter das zu verbieten weil per se zu sagen das wird jetzt verboten ist natürlich -- #1:33:32-34.14#

m(?): (unverständlich) #1:34.14-19#

S.B: Bitte. #1:34.19#

m(?): Also mein Punkt war zu der Kommunikation. Wie wird das kommuniziert am Ende? Es gibt ja diesen allgemeinen neuen Diskurs,wo viele Dinge verboten werden auf der EU-Ebene. Der Prozess, wie es zu diesen EU-bestimmungen kommt, der sehr undurchsichtig ist. Eben hier in Österreich wird gerade über etwas diskutiert, was vielleicht später verboten wird, und Leute in Österreich regen sich dann später darüber auf, dass sie nicht mitbestimmt haben .Also dass auch diese Balance dieses Projektes sichtbarer gemacht wird. #1:34:20-55#

w(??)Ja, zu diesem Sichtbarmachen. Ich bin ja schon ein bisschen älter auch und ich habe seit einem Monat Facebook, weil ich es für die Arbeit brauche. Und über diese social Media Sachen bekommt man wirklich sehr viele Informationen. Also dieser Plastik Anteil im Plastik ist ein ganz großes Thema im Facebook. ch glaube damit kann man auch wirklich sehr viele Leute erreichen. Ich glaube, dass ist ein wirklich tolles Tool, ich meine für die, die es interessiert, weil wen es nicht interessiert, der klickt es weg. Was mich jetzt ein bisschen irritiert ist die Frage ob dieses Projekt abgeschlossen ist? Ich meine, soll das wirklich in 4 Jahren fertig sein? #1: 34.56-35:46#

S.B.: So wie es jetzt konzipiert ist, ist es auf diese Zeitspanne ausgerichtet. Die Idee ist natürlich das es weiter geführt wird, aber wenn ich das richtig verstanden habe ist die Finanzierung für die Zeit danach nicht zugesagt. #1:35:46-58#

M.U: Ja #1:35:58#

w(?): Ja also dann, wenn ich mir etwas wünschen darf, dann wünsche ich mir das so ein Monitoring für zukünftige Substanzen, da wir ja sehr innovativ sind und sicher noch viel mehr finden was für unsere Umwelt schädlich ist, gibt. Das solche Institutionen bleiben dürfen und weiter geführt werden. #1:36:00-22#

w(?): ja ich bin der Meinung, dass ich mir nicht ständig erwarten kann dass die Politik mein Leben regelt, sondern dass es ganz wichtig ist das wir selbst neugierig bleiben. Wenn wir bei der Jacke bleiben; Ich habe nicht hinterfragt warum die jetzt so toll ist. Ich habe mir gedacht super die kaufe ich. Aber ich war eigentlich selbst zu dem Zeitpunkt zu faul oder zu uninteressiert, dass ich nachgefragt hätte warum die jetzt so toll ist. Es wäre aber ganz wichtig glaube ich, weil auf diese Art und Weise wiederum auch die Verkäufer zum Nachdenken angeregt werden. So das eigentlich von Unten nach oben wieder mehr Bewusstsein geschaffen. Das man nicht immer schaut das von Oben nach Unten was runtergestreut wird auf uns. #1:36:28-37:07#

m(?): Zu dem Projekt und zu dem was die Dame gesagt hat. Ich will jetzt nicht sagen in 4 Jahren treffen wir uns hier wieder und diskutieren das ganze Ergebnis durch. Man spricht in der modernen Wissenschaft ja immer von Feedback. Also was kommt wirklich? Das wäre natürlich für die Teilnehmer, unter Umstanden, auch interessant. Ich weiß nicht was in 4 Jahren hier ist, aber es wäre vielleicht auch eine Aufgabe für das Umweltbundesamt, dass man sagt diese 4, 5 Jahre haben wir diesen Versuch laufen und wir veröffentlichen die Ergebnisse auch wirklich. Der Wunsch ist ja schon öfter artikuliert worden. Wer interessiert daran ist, kann dann nachfragen was geschehen ist und kann dann unter Umständen auch selber wieder einen Input an euch geben. Macht weiter, vielleicht kann man dann auch den jeweiligen politischen verantwortlichen sagen, dass sie schauen sollen das wir Geld kriegen weil die Bevölkerung interessiert daran ist das dieses Projekt weiter geführt wird. #1:37:09-38:12#

w(J): Jetzt einmal was Konkreteres. Es wäre vielleicht eine ganz nette Idee wenn man sagt, dass die Leute die diese Produkte verkaufen, nämlich nicht die, die sie herstellen sondern die, die wirklich im Geschäft stehen, die als Student eine Studenten Job haben. Wenn man sagt, dass das Geschäft denen eine Schulung geben soll, so dass die auch über die negativen Seiten von den Produkten aufgeklärt werden. So, dass wenn ich jetzt als Kundin hingehe diese Informationen haben möchte, ich mich beraten lassen kann. Das ich nicht nur die Werbung kriege sondern, auch positive und negative Effekte, die diese Jacke auf mich haben wird. Was einerseits für den Kunden gut ist, weil er informiert wird, andererseits sind das Leute, die nicht ihr Leben lang in diesem Geschäft Outdoor-Jacken verkaufen, sondern vielleicht irgendwann ihren Job wechseln, das Wissen bleibt ihnen aber und dadurch hat man dann Leute in der Bevölkerung, die das alles einmal gehört haben und dadurch ist das Wissen auch vorhanden, das wäre vielleicht eine nette Sache.#1:38:13-39:26#

m(Y):Nur so ein kleiner Wunsch noch, besonders weil es unbezahlt ist, explizit an das Bundesamt. Wir wissen, dass Gesetzgebung sich ändern kann. Wir wissen (unverständlich). Ich wünsche mir von Ihnen dass Sie dafür einstehen das dieses Projekt auf freiwilliger Basis der Probanden bleibt und das sage ich absichtlich, damit es keine Zwangs Überwachung oder Massendatensammlungen gibt, nur weil das das Auswerten vereinfacht, denn das hat negative Aspekte, und das möchte ich auch in diesem Kontext sagen. Das wars#1:39:26-40:00#

w(segel): Was man natürlich auch als sanfte Form eines Verbotes einführen kann, ist dass man gewisse umweltschädliche Substanzen (unterbricht sich selbst) Ich meine, ich war bei Tetrapack beschäftigt, in der Umwelt Abteilung, und bin vor 20 Jahren dafür geprügelt worden das es uns nicht gelungen ist aus einer Einweg-Verpackung eine wiederbefüllbare Verpackung zu machen. Also das ist mein persönlicher Hintergrund von der Industrie. Ich möchte dass nur noch einmal sagen und eigentlich diese Härte und Schärfe würde ich mir erwarten wenn es umso schwerwiegende, Schadstoffe anbelangt. Da würde ich mir eigentlich erwarten, dass diese Schärfe wieder aufgenommen wird wenn es um solche Umweltschädigenden geht, die auf lange Frist vorkommen und sich umweltschädigende Chemikalien in Produkten befinden. Vielleicht, dass man irgendeine Form des Beipackzettels unterschreiben muss, als Konsument. Das es da eine Informationspflicht gibt, die sagt dies und das und wenn du lieber Konsument diese Jacke kaufst, dann musst du dir im Bewusstsein darüber sein, dass das und das riskierst und die Entsorgungsmaßnahmen, oder die bei der Pflege, sind einzuhalten. Also ich sehe nicht ein warum man da weiter irgendwie so liebäugelt mit irgendwelchen Stoffen, die nachweisbar gesundheitsschädigend sind, und umweltschädigend. oder? #1:40:03-41:45#

m(?): Wissen sie warum Salz ein Ablaufdatum hat? #1:41:48-51#

w(segel): Weil es ein Lebensmittel ist. #1:41:51-55#

m(?): Nein #1:41:55#

w(segel): Warum? #1:41:57#

m(?): Weil die Konsumenten, teilweise über die Industrie, ein Haltbarkeitsdatum gefordert haben. Weil der Konsument darauf trainiert ist, dass er schaut ob es ein Ablaufdatum oder Mindesthaltbarkeitsdatum gibt. Ein Schulkollege von mir hat bei den Salinen gearbeitet. Die Salinen haben sich mit Händen und Füssen gewehrt ein Haltbarkeitsdatum einführen. Weil was passiert? Das Salz ist abgelaufen #1:41:58-42.23#

w(segel-unterbrechend): Das ist ja krank, oder? #1:42:25#

m(?): Ja. #1:24:26#

w(segel): Mein Mann hat mir unlängst Gewürze weggeschmissen und Tees und Safran. (Mann unterbrechend: WARUM?) Wir haben so viel Geld ausgegeben für natürlichen Safran und der hat das weggeschmissen (Mann unterbrechend: Das ist doch krank. Warum?) Weil es nicht irgendwie (stimme wird übertönt) #1:42:27-38#

Sehr viel wirres Gerede im Hintergrund.

m(?): Wir geben einen Stoff hinein, das es mehr rieselt. Was haben unsere Vorfahren gemacht?.

w(?): Reis rein getan #1:42:48#

m(?): Ja oder den Salzstreuer mal am Tisch (schlägt auf den Tisch) gestellt, dann hat es auch funktioniert. Also da kommt, in dem Fall sag ich jetzt absichtlich der Konsument ist schon so poliert, (w(segel)-unterbrechend: JA! er hat Angst, er hat Angst!) dass das geht. Deswegen gibt es ja das nette Bildchen auf dem steht das Mindesthaltbarkeitsdatum nicht gleich heißt das es ab dann tödlich ist. #1:42:49-43:07#

S.B: Gut, wenn es jetzt keine konkreten Erwartungen und Wünsche mehr an 2022 gibt, dann würde ich sagen wir machen eine kurze Pause, lüften und landen dann wieder 2018. (allgemeines Gelächter ) #1:43:08-23#

S.B.: Gut also ich habe angekündigt, dass wir zurück aus der Zukunft kehren und wieder im Jahr 2018 sind. Vorher noch kurz was Organisatorisches, also es wird jetzt eine Abschlussrunde geben und dann werde ich diesen Zettel austeilen, auf dem es darum geht dass sie ihre Aufwandsentschädigung kriegen und wir dafür einen Beleg haben. Darauf ist nochmal aufgeführt ist dass diese Diskussion aufgezeichnet wurde und dass die Daten in anonymisierter Form verwendet werden. #1:43:30-44:02#

m(?): Noch eine kurze Frage. Ist das ein EU-Projekt, das sie machen? #1:44:03-5#

viele auf einmal : Ja! #1:44:06#

m(?): Ich hatte einmal Probleme, weil auf einer Teilnehmerinnenliste nicht alle unterschrieben hatten, es wäre vielleicht nicht schlecht wenn alle die hier sind unterschreiben. #1:44:07-19#

Stimmengewirr im Hintergrund.

m(?): Oder genügt Ihnen das? Also weil ich habe einmal was abgerechnet, da gab es langes Hin und Her. #1:44:20-28#

M.U.: Also ich glaube wir werden das hier kopieren, einmal für die Kassa und einmal für uns. Und ich glaube das reicht dann, oder? #1:44:32-36#

m(?): Ja, ja. na gut wenn das genügt, ja, ja dann #1:44:37-39#

w(?): nur zum abrechnen #1:44:40-41#

M.U.: ( unverständlich-hat aber vermutlich mit den Zetteln zu tun mit denen sie hantiert)

w(?): Danke! #1:44:48#

m(?): Ich wollte sie nicht maßregeln sondern nur mögliche..

w(?): danke für das mitdenken #1:44:52-53#

Allgemeines lachen.

S.B.: Gut also wir sind im Jahr 2018. In dieser abschlussrunde geht es um unseren Alltag. Also wenn Sie jetzt an ihren Alltag denken: glauben Sie, dass die Ergebnisse von Human Bio Monitoring. und insbesondere dieser Initiative, für Sie relevant sind? Und wenn ja, an welche Themen oder Bereiche oder Fragestellungen aus Ihrem Alltag denken Sie da dabei? (pause) Gut, wie haben vorher hier angefangen, vielleicht wenn es für sie passt fangen wir jetzt hier an?#1:45:01-44#

m(?): Gut, danke. Weil ich nicht genau weiß um welche Stoffe es sich jetzt handelt tue ich mir schwer mit der Zuordnung, und damit darüber nachzudenken wo mich das im Leben treffen kann. #1:45:45-54#

S.B.: Aber sie können auch Wünsche im Bezug darauf äußern, welche Stoffe angeschaut werden sollen. Also aus der anderen Sicht denken. #1:45:54-46:00#

m(?): Wenn ich es aus der anderen Sicht andenke, dann wäre es in dem Fall die Landwirtschaft mit all ihren Dingen, weil es da auch sehr viele Folgeprodukte gibt. Das man mehr Erfahrung als Außenstehender bekommt, mehr Informationen darüber mit den Stoffen passiert? Weil, wenn ich das zwar Oben reinleere aber Hinten kommt etwas anderes heraus, und die Stoffe Unten machen erst die Probleme, die Oben sind sauber, dann haben wir wenig davon. Das wäre schön #1:46:02-36#

S.B: Und wenn Sie jetzt von der Landwirtschaft sprechen, an welche Stoffe oder Produkte oder an was denken Sie da konkret? #1:46:36-46#

m(?): Na, da kann man bei den Beizmitteln anfangen über sämtliche, sogenannten, Pflanzenschutzmittel bis hin zu den (unverständlich) Naja, ein Apfel wird zirka 52 mal gespritzt bevor sie in kaufen (lachen im Hintergrund) (unverständlich)

S.B.: Mhm (bejahend) Dankeschön. #1:47:19#

m(älter?) Ja ich habe schon vorhin gesagt, für mich ist es schon alleine aus dieser Veranstaltung heraus gegangen, dass ich mir jetzt wieder überlege keine Pflegemittel mit Treibstoffgasen mehr zu kaufen sondern brav zur alten und bewährten Schuhcreme zurück zugreifen werde. Obwohl ich zugebe, dass ich mich in meiner Bequemlichkeit etwas eingeschränkt fühle. Es ist ja einfacher: ich putze den Schuh einmal mit einer Kott Bürste ab, halte ihn auf 30, drücke drei Mal darauf und die Geschichte ist fertig. Und so muss ich Pasta nehmen und muss da schrubben. Aber es bringt etwas. Mein Wunsch wäre, da bin ich auch auf ihrer Linie, dass der Bevölkerung klargemacht wird, dass sie aufpassen sollten was in den Produkten drinnen ist und das man sein Verhalten ändern kann. Ich bin nicht unbedingt dafür, dass man einen ganzen Katalog macht, da begeben wir uns nämlich unter Umständen in ein juridisches Dunkel Feld, wo es gegeben falls zu (unverständlich) führen könnte, was wir nicht wollen, also was ich nicht möchte, weil man sonst möglicherweise auf Schadensersatz klagen kann. Jeh mehr reguliert wird umso eher gibt es diese Gefahr. #1:47:21-48:26#

w(Segel): Wenn ich nur die Basisstoffe nenne, was die alles anstellen können.. #1:48:26-30#

m(?): Nein, nein. Weil sie gesagt haben einen Leitfaden für die Familie #1:48:30-33#

w(Segel): Nein es geht um die Stoffe, die einfach die Umwelt nachhaltig schädigen, und dadurch auch den Menschen. Die Basisstoffe, so wie früher. #1:48:33-45#

m(?-währenddessen und länger): Tzzzz! Die Information muss gegeben werden. Wenn das jetzt als Ergebnis dieses Monitorings herauskommt, dass man die Bevölkerung darüber mehr informiert. Ich wiederhole mich jetzt, es könnte mit sozusagen negativer Werbung gemacht werden. Bitte passt auf in diesem Stoff ist das. Oder diese Stoffe gibt es und die sind überall dort und dort drinnen, so wie die Weichmacher, die sind gefährlich für euch Menschen, passt auf. Das wäre die Erwartung. Und das ist die Realität jetzt (zustimmendes mhm im Hintergrund #1:48:35-49.11#

lachen

m(Y): Naja, man muss aus verschiedenen Perspektiven denken. Dieses Problem ist ein wunderschönes Exempel. Ich wollte fragen ob diese Giftstoffe in der Produktion hergestellt werden, die da rauskommen, oder sind die im Produkt selbst? Oder Beides?#1:49:15-30#

M.U.: Bei der Erzeugung, wird das verwendet #1:49:30-33#

m(Y): Bei der Erzeugung wird das verwendet. Und geht dann der Stoff durch einen chemischen Prozess wieder hinaus? Wissen sie das? oder wissen sie das nicht? #1.49:33-39#

M.U.: Also die Perfluor-Substanzen bleiben im Prinzip schon darin. #1:49:39-47#

m(Y): Also sie bleiben drinnen, ok. Nachdem leider in gewissen Arbeitsbereichen nicht auf (unverständlich). Wir brauchen Alternativen für solche, manche Berufe sind nicht dafür ausgelegt. Sie haben Recht, für einen Spaziergang mit einem Kind ist das nicht so gut. Aber für einen Maurer ist kein wasserfestes Gewand sehr unerfreulich. Und ich persönlich, würde mich auch nicht freuen, weil in meinem Berufszweig wasserdichtes Gewand genauso relevant ist. Es wäre auf jeden Fall so dass ein Alternativstoff her muss. Es muss auf jeden Fall irgendwas her. Ansonsten glaube ich, das haben sie eh schon Großteiles angesprochen, das man es eingrenzen kann. #1:49.48#

w(jung?) Ja, ich denke mir im Prinzip wenn ich mir Sachen kaufe und bewusst zulege, dass ich weiß was da drinnen ist. So dass, ich mich bewusst dafür oder dagegen entscheide. So dass ich sagen kann, ok das ist es mir auch wert das ich hier dieses und jenes Risiko habe und dafür habe ich dann diesen Vorteil von dem Produkt. Und wenn ich das nicht will gibt es vielleicht diese oder diese Alternative, die vielleicht selbst nicht so gut sind. Also dass das eine bewusste Entscheidung ist. #1:49:-51:08 #

m(?): Ich bin immer ungern der Schwarzmaler aber ich habe heute in der Runde gesehen, dass dieses gesamte Thema sehr komplex ist und auch auf so vielen verschiedenen Ebenen auch emotional gelebt wird. Ich weiß nicht ob überhaupt irgendwas dabei heraus kommen würde. Was ich gerne hätte am Ende wäre die Klarstellung ob die Belastungen auch gleich Gesundheitsgefährdungen sind, also ob diese Stoffe gleich irgendeinen schlechten Output für den Menschen haben? Und dann würde ich gerne wissen wo er mir im täglichen Leben begegnet und was ich dann theoretisch vermeiden kann. #1:51:13-52:08#

S.B.: Und gibt es irgendwelche Bereiche in ihrem Alltag, bei denen Sie sagen, dass Sie es für diese gerne besonders genau wissen wollen oder da ist es für sie- #1:52:010-17#

m(?)- unterbrechend (dahinter w 3 leise weiter): Nein das würde ich ganz vom Stoff abhängig machen. Davon wie gefährdend er wirklich ist. Es ist mir letztendlich egal woher er kommt, wenn er wirklich gesundheitsschädigend sein sollte. #1:52:17-34#

S.B: mhm (bejahend).

w(ältere?): Wenn es möglich ist, würde ich auch gerne wissen, wie Dinge erzeugt werden, was diese für Schäden haben, was es für mich positives und negatives bringt. So, das ich selber entscheiden kann ob ich etwas will oder nicht. Besonders bei landwirtschaftlichen Lebensmitteln, also nicht bei Genussmitteln sondern bei Lebensmitteln. Wo kommen sie her? Sind sie gespritzt oder dergleichen? Ist das schädlich für mich oder ist das schon wieder weg? Das würde mich interessieren. Das wäre das, was für mich interessant ist. #1:52:40-53:17#

m(?) : Also für mich sind jetzt spontan gesagt vier Bereiche wichtig. Das ist Ernährung, das ist Kleidung, also Stoffe in der Kleidung, Stoffe in Reinigungsmitteln und Stoffe in Baustoffen. Das Kernthema ist natürlich Gesundheit. Also: wie vermeide ich Schädigung? Wie kann ich Schädigungen vermeiden? #1:53:18-53:51#

S.B.: Danke. #1:53:58#

w(segel) Ich sehe in den fächerübergreifenden Tätigkeiten, die ich ausübe, eigentlich einen Auftrag an mich, und zwar weiterhin Menschen dahingehend zu beraten, dass sie trotz Umwelt Einwirkungen gesund bleiben können und sie zu ermutigen, dass jeder für sich die persönliche Ernährung findet, die für ihn gesundheitsfördernd ist. Und vor allem auch wie man sich zu einem umweltfreundlichen Verhalten entscheidet. Ich möchte den Konsumenten weiterhin dazu ermutigen dass nicht alles was irgendwie neu ist oder gehyped wird, erstrebenswert ist und das wir eigentlich für Vieles aus der Vergangenheit gute alternativen haben auf die man zurückgreifen kann. Das ist mein Job, das habe ich aus der Diskussion mitgenommen und auch noch vorsichtiger zu sein im Bezug auf umweltschädliche Substanzen. Ja. Also nicht das jemand vier Segeljacken oder Overalls oder was auch immer braucht. Sondern das man der Person sagt, dass sie doch eine hat, auf die sie achten soll und die sie pflegen soll, dann kommt sie auch lange damit aus es findet weniger Umweltbelastung statt. Also das ist jetzt mein Job. Das nehme ich mit. #1:53:58-55:21#

S.B.: Danke #1:55:21#

w(?):Nachdem ich einmal Ernährungswissenschaften und Umwelt studiert habe, und schon in dieser Zeit angefangen mich damit zu beschäftigen, glaube ich schon, dass ich so ein bisschen etwas weiß aber eigentlich auch gar nichts. Ich habe damals schon die Ansicht geboren, dass alles quasi multi-kausal und multi-faktorell ist. Deswegen finde ich diese Untersuchungen wahnsinnig wichtig und zwar in allen Bereichen Ich könnte jetzt nicht sagen was mich jetzt mehr interessiert, weil es geht um unsere Umwelt, es geht um Menschen, den Menschen, bei den Frauen, geht zum Beispiel auch um Fertilisation, es geht bis zu den kleinsten Mikroorganismen. Egal was man sich anschaut, also Silber auch, also ein ganz normaler Stoff. Ich finde es einfach wichtig und ich wünsche mir, dass das weiter geht und noch viel intensiver wird. Und dass man eben etwas dagegen macht, das im Menschen diese ganzen Hormonkaskaden sind, bei denen man schon weiß dass darauf Einfluss genommen wird. Das all das viel ernster genommen wird. Im Endeffekt müssen wir immer wieder die Industrie dazu bringen so etwas nicht zu produzieren (lachen im Hintergrund) #1:55:24-56:50#

S.B.: Danke. #1:56:51#

w(??): Mir ist heute Nachmittag bewusst geworden, dass ich mich eigentlich unbewusst schon mit sehr vielen Dingen hier beschäftige, sei es von der Ernährung her oder auch vom Beruf her. Auch beim Hausbau haben wir schon auf natürliche Materialien geschaut und das Haus nicht mit so viel Styropor zu gedämmt und was, weiß ich was, alles. Damals haben dazu viele gesagt, dass wir einen Vogel haben. Aber ich habe das Gefühl, dass es sich in diesem Haus anders lebt. Ein Thema, das mir erst diesen Nachmittag bewusst geworden ist, ist das auf meinem Nacht Kästchen ,,Die Akte Glyphosat‘‘ liegt. Ich beschäftige mich mit diesem Thema weil ich nicht weiß: ja oder nein? Und ich hoffe dass, ich da vielleicht in den nächsten Jahren Unterstützung kriege, so dass ich dann wirklich ja oder nein sagen kann. Es gibt auch so viele Pro und Kontra. Die einen sagen Ja, die anderen sagen Nein. Ich weiß nicht wem ich vertrauen kann, daher muss ich für mich selbst eine Antwort finden. Daran arbeite ich gerade bei diesem Thema. #1:56:53-57:53#

m(?): Mir geht es vor allem wieder um die Kommunikation, glaube ich. Und zwar in der Richtung wie wird möglich unaufgeregt und sachlich kommuniziert werden kann am Ende. Wie kann ein Thema, wie zum Beispiel Glyphosat, eine gewisse Unaufgeregtheit haben und wie kann man als Institution, als EU zum Beispiel, in diesen medialen Diskurs eingreifen und beruhigend wirken und aus dem Ganzen ein bisschen die Emotion heraus nehmen. Was ich bei den Dingen bemerke, ist dass ich auch finde das es so ein Grenzdiskurs ist. Also, dass die Grenze zwischen Mensch und Umwelt die jetzt immer wieder auftaucht und dann erst relevant wird wenn es die Gesundheit vom Menschen geht und es wäre gut wenn diese Ebene vielleicht einmal explizit kommuniziert wird. Das man versucht darauf aufmerksam zu machen, dass das ein Ding ist und man das nicht trennen kann, genauso wie ich die EU als Projekt sehe bei dem man versucht Grenzen abzubauen.#1:57:55-59:16#

m(akzt): Diese Ergebnisse sind auch für mich relevant, vor allem in den Bereichen Reinigungsmittel, Lebensmittel, Medikamente oder kosmetische Mittel im Alltag. Ich denke diese Initiative muss eine Brücke zwischen der Regierung und der Industrie sein. Das spielt eine sehr große Rolle, glaube ich #1:59:22-53#

w(??): Wie für jeden sind natürlich auch für mich die Ergebnisse sehr relevant. Ich würde auch sehr gerne wissen wenn ich etwas einkaufe, was in dem Produkt steckt und welche Auswirkungen es auf meinen Körper hat. Vor allem bei Lebensmitteln, Getränken, Pflegeprodukten und Reinigungsmitteln stecken wirklich sehr viele Stoffe drinnen und von vielen wissen wir die Auswirkungen leider nicht. Und deshalb will ich auch gerne die Informationen bekommen. #1:59:55-2:00:30#

w(jünger?): Ja, es ist jetzt eigentlich eh schon fast alles genannt worden, aber ich denke für mich ist es auch relevant. Es ist eben sehr kompliziert. Es hängt von vielen Faktoren ab, die man vielleicht als einzige Person nicht ganz überblicken kann. Ich würde mir wünschen, dass ich am Ende des Projekts dann sagen kann: ,,Ok, eben ich entscheide mich jetzt dafür, mit den und den Konsequenzen oder dagegen. Und zwar nicht nur Konsequenzen für mich, sondern eben auch die für die Umwelt und eben auch für andere. #2:00:33-01:14#

w(?): Also diese sozialen Effekte? #2:01:18#

w(jünger): Genau, genau. #2:01:22#

S.B.: Ja, fein. Vielen, vielen Dank! #2:01:29-30#

m(??-älter?): Ich möchte noch etwas anhängen. Es war vor etwa 3 oder 4 Wochen im Fernsehen mal so ein Aufschrei, kann man fast sagen, ich weiß nicht von wo her er kam, das habe ich mir nicht gemerkt, aber vielleicht war es sogar vom Umweltbundesamt. Frisch gekaufte Kleidung möge man, vor dem ersten Anziehen, waschen. So etwas ist doch schon ein Hinweis, an einen normal denkenden Menschen. Der muss sich ja fragen was da drinnen sein muss, wenn es sogar medial verbreitet wird. Und das wäre auch so mit dem Ergebnis dieser Studie, das soll medial verbreitet werden und die Leute darauf aufmerksam gemacht werden so dass der eine oder andere vielleicht um denkt. #2:01:34-2:02:07#

S.B.: Danke für ihre wertvollen Beiträge. Danke für ihre Zeit. Ich würde jetzt wie angekündigt dieses Formular ausfüllen---- #2:02:17-#

**Transcript of the Portuguese focus group (May 2019)
FOCUS GROUP – HUMAN BIOMONITORING**

Date: May 29th, 2019

Location: Institute of Environmental Health, Faculty of Medicine, University of Lisbon, Lisbon, Portugal

Recording time: 1:43:51

M – Moderator; CM – Co-moderator

**M**: Bom, muito obrigado, mais uma vez, por terem vindo até cá *(...hã...)* há aqui algumas caras que eu já conheço, outras que são novas *(...hã...)* se calhar começávamos por fazermos uma breve apresentação de... Eu sou o A., (...), trabalho aqui nesta casa, na Faculdade de Medicina, (...) e *(...hã...)* e nós temos estado envolvidos num projecto já há coisa de 2 anos que é o HBM4EU *(...hã...)* que no fundo é um projecto de biomonitorização, e *(...hã...)* dentro desse projecto pediram-nos para fazer *(...hã...)* o projecto tem muitas dimensões, eu já foi explicar um pouco mais do projecto *(...hã...)* mas pediram para falarmos um pouco sobre essas coisas da biomonitorização que, para vocês, alguns, se calhar, estão familiarizados com o termo, outros não tanto, quer dizer, é um pouco desbravar um pouco o caminho *(...hã...)* o que é que é esta coisa de biomonitorização, para que é que isto serve, e por aí fora. *(...hã...)* Eu se calhar começava pela, por uma coisa simples que era *(...hã...)* apresentar-nos uns aos outros, não é? *(...hã...)* Vocês já, já preencheram aí o consentimento informado, não já? *(...hã...)* Portanto, como, como devem ter visto, como já explicámos, esta conversa vai ser uma conversa muito informal *(...hã...)* Eu e o, e o G. temos uma tarefa muito simples que é, no fundo, ouvir-vos a conversar *(...hã...)* e de vez em quando *(...hã...)* vamos dando aqui umas, umas dicas para irmos para um lado ou para o outro, mas basicamente é conversar, e é conversar sobre aquilo que pensam sobre o assunto e nós vamos levantando aqui umas ideias para vocês depois debaterem essas ideias da maneira como entenderem. Não há regras nenhumas, desde que não andemos todos à batatada, tudo bem *(...hã...)* e, portanto, podem discordar à vontade, aliás, é mais divertido até quando discordam *(...hã...)* do que quando concordam, portanto, é uma conversa mesmo muito, muito simples. Portanto, vamos dar uma volta para ficarmo-nos a conhecer. B.?

**B.**: Portanto, sou o B., trabalho aqui na (...) já conheço a maior parte das pessoas aqui participantes, trabalho aqui no (...) e, portanto, tenho colaborado com as pessoas (...). Tenho muito gosto nisso. É isto.

**C.**: C., presto aqui apoio (...) ao ISAMB, entre outras, outros clientes, naturalmente, mas é com muito gosto que venho sempre aqui *(...hã...)* trabalhar com esta equipa fantástica do ISAMB *(...hã...)* essencialmente é isso, portanto, (...).

**D.**: Sou a D., *(...hã...)* já estou aposentada, *(...hã...)* trabalhei durante 39 anos na Polícia Judiciária. Em relação à matéria sou uma leiga. *[riso da própria]* É o conhecimento do dia-a-dia *(...hã...)* mas disposta a colaborar.

**E.**: O meu nome é E. e sou psicóloga clínica e trabalho em investigação clínica (...).

**F.**: Eu sou a F., sou fisiologista do exercício, trabalho (...) e não tenho formação neste campo, mas venho com mente aberta e disposta a dizer o que (...) penso.

**CM**: O meu nome é G., sou biólogo, sou investigador (...) e a minha função aqui neste, neste *focus group* é co-moderar *(...hã...)* a sessão, juntamente com, com o A. Eu gostaria só pedir um favor *(...hã...)* que é *(...hã...)* fazer um esforço para cada, para falarmos cada um na sua vez, por uma razão muito simples, é que depois na desgravação é complicado quando as vozes se sobrepõem, portanto, às vezes temos tendência para sobrepor as vozes, pedia só este favor de, de falarmos cada um na sua vez. Muito obrigado.

**H.**: Sou o H., sou de engenharia aeroespacial, e venho aqui em nome dos (...), sou um dos colaboradores.

**I.**: Olá, eu sou o I., sou escravo na (...). *[risos dos vários participantes]* (...) e *(...hã...)* quando me fizeram *(...hã...)* este desafio, quando me fizeram esta proposta, eu aceitei-a de braços de abertos, não que esteja muito dentro do tema, mas porque gosto de dar o meu contributo naquilo que for possível.

**J.**: Já vi que isto é bom, porque em relação ao tema somos todos leigos, não é? *[riso do próprio]* O que é bom. *(...hã...)* Chamo-me J. A minha formação de base é a sociologia, mas trabalho na (...) *(...hã...)* *[riso de um outro participante]* Olá (...). *(...hã...)* Por acaso acabo por estar aqui também presente em algumas sessões e é sempre bastante interessante.

**K.**: Eu sou a K., sou psicóloga. Neste momento, infelizmente, não estou a, a trabalhar *(...hã...)* não sei nada sobre o tema, mas gosto de desafios, já participei em alguns, *(...hã...)* pronto, disposta a participar.

**M**: Muito bem.

**L.**: Sou a L., venho também dali dos (...), e (...) e sou doméstica, portanto, mas tenho um quintal e gosto muito de andar lá e arrancar as ervinhas e essas coisas para não por os venenos.

**M**: Para não por os venenos.

**L.**: Para não por os venenos.

**M**: OK. Vamos pegar exactamente nisso dos venenos. Todos nós estamos expostos a muitos químicos, não é, estamos expostos a substâncias químicas no nosso dia-a-dia, não é, algumas até da roupa que vestimos, outra *(...hã...)*, sei lá, as senhoras usam mais cosméticos do que os homens e os cosméticos também podem ter lá umas coisitas, não é, que depois passam para a pele *(...hã...)* depois temos a comida, temos a poluição do ar, não é *(...hã...)*. Quando nós pensamos nestas, na quantidade toda de poluentes e de *(...hã...)* produtos como metais pesados, como, coisas que, no fundo, entram em contacto connosco, que nós vamos absorvendo ao longo da vida *(...hã...)* em que é que vocês pensam, o que vos surge logo à cabeça? Se calhar, vamos fazer aqui um jogo. Respostas rápidas. O que é que vos surge logo à cabeça, por exemplo, a ti, F.

(06:58)**F.**: Sobre?

**M**: Que poluentes, químicos é que te preocupam mais.

**F.**: *(...hã...)* Poluição atmosférica no geral. Poluentes que saem dos tubos de escape dos carros.

**M**: OK. D..

(07:11)**D.**: Alimentação.

**M**: Alimentação. E na alimentação, que tipos de coisas é que lhe salta logo à cabeça?

**D.**: *(...hã...)* Uma coisa que eu evito o mais possível, por exemplo, é produtos com conservantes. Evito o mais possível.

**M**: OK. B.?

(07:27) **B.**: Estava a pensar nos refrigerantes.

**M**: E o que é que te preocupa nos refrigerantes?

**B.**: Quase tudo. *(...hã...)* Os refrigerantes podem ser os aromatizantes, podem ser os corantes, podem ser os conservantes *(...hã...)* Tive uma experiência um bocado traumática que, para uma coisa tipo *twilight zone*, mas isto aconteceu mesmo, foi deixar um refrigerante demasiado tempo aberto no frigorífico e quando o deitei fora, o que eu deitei fora não foi um líquido, foi uma espécie de uma pasta. Eu fiquei muito mal, quer dizer, nauseabunda, e eu fiquei a pensar, do que isto é feito, quer dizer, para acontecer isto.

**M**: OK. OK. Conservantes. Mais.

(08:07)**H.**: *(...hã...)* Principalmente na poluição dos rios, descargas *(...hã...)* da parte de, das indústrias para os grandes afluentes que depois são tratados para nosso consumo.

**M**: OK. E lembra-se de alguma coisa, algum, algum tipo de substância mais em concreto que...

**H.**: Agora nos últimos casos têm sido as descargas de celulose no rio Tejo, por exemplo.

**M**: OK. OK.

**H.**: E na zona onde eu nasci existe muito a problemática de, de químicos, acetonas e tintas, *(...hã...)* que são descarregadas directamente nos rios.

**M**: OK. I.?

(08:46) **I.**: Fenofetalatos ou como é que eles se chamam *(...hã...)* São aquelas coisas que os plásticos que nós usamos todos dias transmitem aos líquidos que bebemos, à comida que comemos, às comidas que cozinhamos nos microondas, dentro da embalagem de plásticos, a todos esses contactos que temos, como foi dito há pouco e muito bem, também com a roupa que vestimos, aquilo em que mexemos, e o que isso está a causar inclusivamente na nossa descendência. Naqueles casos que temos ouvido de crianças que, que vêm com problemas vários e depois, quando se faz precisamente a biomonitorização, vem-se a descobrir que podemos relacionar alguns desses problemas com estes, com estes *(...hã...)* poluentes.

**M**: OK. OK. Nós?

(09:39)**K.**: Amianto.

**M**: Amianto. OK. A primeira coisa que lhe surge à cabeça é amianto.

**K.**: Sim.

**M**: OK.

(09:49)**J.**: *(...hã...)* Curiosamente tive uma conversa, *(...hã...)* por questões profissionais, tive contacto com duas pessoas da indústria agroalimentar *(...hã...)* Uma da área das conservas de peixe e outra da área dos frangos e fiquei absolutamente horrorizado com aquilo que me disseram *(...hã...)* E, portanto, penso em alimentação, é a nível de alimentação, sim.

**M**: OK. C.?

(10:17)**C.**: *(...hã...)* Eu penso que a gente vive numa sociedade de consumo e se a gente vai pensar em tudo o que faz mal, a gente de manhã não nos pomos a pé. Eu acho que há muita informação, há muitos estudos, felizmente, que nos vão alertando para todos os problemas que há, mas esses problemas estão aqui, a gente tem que os viver com eles. Por vezes, esse excesso de informação talvez seja prejudicial, se calhar mais prejudicial que os químicos, acho eu, para muita gente. De qualquer maneira, de qualquer maneira, *(...hã...)* é preocupante a gente saber que esses químicos fazem-nos mal, mas a gente realmente até na roupa e tudo, tudo, *(...hã...)* os plásticos, o, tudo, em tudo, a comida, os fertilizantes, etc., deixa-nos um pouco preocupados, se calhar o excesso de informação também é prejudicial.

**M**: OK. Temos aí um agente que não é químico, mas também é, também é prejudicial.

**C.**: É. Não porque deixa-nos preocupados e a gente preocupados dura menos. Acho eu.

(11:30)**L.**: Eu também vou para os fertilizantes, porque nós acabamos por comer muitos químicos que são utilizados na agricultura, portanto, eu nasci numa aldeia, as coisas, sei como eram tratadas, semeadas, plantadas, e agora não é assim, agora é químicos, se nós pensarmos que a batata, que é uma coisa que nós comemos praticamente todos os dias, e a quantidade de químicos que ela leva desde que é posta na terra, que é sulfatada depois por causa do escaravelho, mais os adubos, mais não sei quê, mais depois quando é arrancada o pó para a borboleta, o pó para não grelar, o pó para não sei o quê, e, e portanto, a quantidade, a mim preocupa-me muito, essencialmente no comer, porque eu aqui há dois anos, numa alheira que comi, que habitualmente não como, naquilo dia apeteceu-me e comi, e estive muito mal, e apanhei uma, uma alergia que andei quase um ano para conseguir, que nunca percebi, nunca se descobriu o que é que foi o que me faz mal, portanto, foi químicos, qualquer coisa que tinha lá dentro e que eu comi, não estava azeda, não estava estragada, mas o certo é que assim que a comi, ficou-me a doer o estômago logo, e a partir daí, ao fim de meio ano, ainda andava nas consultas no hospital, foi muito complicado.

**M**: OK. OK. Recorde-me só o seu nome?

**L.**: L.

**M**: A L. dá-nos sempre boas, boas ideias para a próxima pergunta que é também falar de ficarem mal-dispostos, apanharem uma alergia, não é?

**L.**: Foi logo. Foi logo. Depois comecei a inchar, a ficar vermelha, o corpo, podia ser hoje numa perna, amanhã num braço, depois na barriga, foi no peito, foi no, no rabo, vá, nas nádegas, portanto, cada dia eu estava vermelha e inchada num sítio diferente e foi muito complicado fazer o tratamento para conseguir ultrapassar aquilo.

**M**: OK. Esta foi a última pergunta que eu fiz a, a indicar quem é que respondia, agora é completamente livre e a pergunta que eu lanço é, vocês referiram aí *(...hã...)* aspectos de vários fertilizantes *(...hã...)*, poluentes que vêm pela via da alimentação, pela via do ar, pela via da água, mas na prática, estão preocupados com o quê? De que formas é que esta, é que estes poluentes, estas, estas substâncias podem prejudicar-vos em quê?

(14:03)**E.**: Eu acho que é o impacto que pode ter também na nossa saúde. Por exemplo, uma das minhas maiores preocupações é o consumo da água. Eu moro perto de várias fábricas que fazem descargas para, para o rio e para, para o mar. E depois, claro, a água é tratada para nosso consumo. *(...hã...)* São fábricas de vários tipos de roupa, que tingem roupa, que fazem outro, outro tipo de fertilizantes, e até que ponto é que, tudo bem, a água está aprovada para consumo e é como diz o C., também não podemos estar completamente alarmistas, mas até que ponto é que o consumo (...) deste tipo de poluentes, entre aspas, no nosso dia-a-dia constantemente, o que é que isso pode ter na nossa saúde. Em termos de alimentação, eu tenho o privilégio de poder plantar algumas coisas para consumo próprio, mas nem toda a gente tem esse privilégio, ou seja, pessoas que compram comida nos supermercados sujeitas a esse tipo de fertilizantes, pessoas que bebem água, pode estar própria para consumo, mas não significa que não tenha quantidades mínimas de, de qualquer químico. A longo prazo, que influências é que isso pode ter a nível, por exemplo, para as mulheres a nível de reprodução, a nível de, de outro tipo de problemas de saúde.

**K.**: Eu, eu, também, eu tenho ali um bocadinho a, a ideia daqui do C. de que se formos atentar a tudo e tudo ser uma preocupação, é mau, também, não é? Portanto, o excesso de alarmismo é, é complicado e, e se formos a pensar em tudo o que comemos, o que vestimos, pronto, não saímos de casa, não é, é melhor não sair. Mas preocupa-me em relação ao futuro, as consequências, não tanto agora, (...) mas para as gerações futuras, o que é que tudo isto, em que é que tudo isto se vai depois transformar, não é, que mundo é que vamos ter, não é quando eu já for muito velhinha, já não existir, não é, mas, porque, creio eu que eventualmente, eventualmente não, provavelmente *(...hã...)* teria sido acomodada, não é, e as coisas...

**I.**: Posso entrar em contradição com eles os dois? *[riso de vários participantes]* Eu respeito muito a vossa opinião e achei fabulosa essa chamada de atenção. Mas eu chamo a atenção, por outro lado, a outro aspecto, que é o aspecto dos sinais. Nós não nos podemos deixar influenciar pelo alarmismo, pelas *fake news*, como agora se diz habitualmente, mas há uma quantidade de evidências que nos estão a alertar, e essas evidências têm a ver com o aumento dos problemas oncológicos. *(...hã...)* Se olharem bem à vossa volta, vão notar que, nos nossos animais domésticos e nas nossas relações, nos últimos anos, tivemos sempre um caso mais próximo de alguém que, infelizmente, *(...hã...)* passou por um problema desses de saúde e, infelizmente, a maior parte das pessoas acabam por não sobreviver...

**M**: Desses, oncológicos.

**I.**: Oncológico, exactamente. E, e há outra coisa que também gostava de, de propor aqui para pensarem sobre isso. Há sinais que são demasiado evidentes para serem ignorados. Eu quando, nesta altura do ano, devido à posição do Sol, quando chego a casa por volta desta hora, e devido à orientação da minha rua, olho no horizonte, eu vejo o ar que estou a respirar, [IMPERCEPTÍVEL] de manhã. *(...hã...)* Há uma quantidade de poluentes que estão no ar e que ainda estão mal estudados. Muitas vezes atribui-se a contaminação do ar ao trânsito automóvel e neste caso, em particular, às emissões feitas pelo tubo de escape, mas atenção que há uma quantidade de coisas que estão a ser descobertas agora. Já há legislação para isso, já há filtragem nos automóveis, automóveis a diesel tem tendência para desaparecer, evidentemente, mas cuidado. Os simples travões e o uso do travão tem um poder de contaminação quase equivalente ao do tubo de escape, porque a fricção dos metais nas pastilhas do travão, e nós muitas vezes não conduzimos da forma mais correcta porque o semáforo está vermelho mas continuamos a acelerar e depois travamos lá em cima, e já começam a aparecer evidências de que isso liberta, para a atmosfera, micropartículas extremamente nocivas. Bom, voltando ao sinais, há, concordo, sim senhor, têm ambos razão, devemos moderar um bocadinho a nossa preocupação, mas cuidado que há sinais evidentes até, como o exemplo que eu dei a olho nu, como se costuma dizer em bom português, que em si são preocupantes. É uma coisa que, que me deixa perplexo: quem tem animais domésticos e vive numa grande cidade, nunca se ouviram como agora, e felizmente para os veterinários porque é bom negócio, a quantidade de animais domésticos que também estão a sofrer de problemas oncológicos e, e, e problemas de outra índole que muitas vezes é associada à contaminação e à poluição, como nós temos nos seres humanos.

**C.**: Eu penso que o equilíbrio passa pela confiança que a gente tem nas autoridades ou nas entidades que devem supervisionar essas emissões, isso tudo. Eu penso que o futuro, quando se tornar mesmo perigoso, pára, estas emissões e isso tudo pára, porque senão morremos todos. E quando, quando chegar a essa altura, isso pára. Até lá isto vai-nos matando devagarinho, a gente se calhar tem menos um ano de vida do que devia ter e, portanto, vai andando, portanto, as coisas vão andando, mas eu regulo-me sempre pela maneira como eu tenho confiança ou não na entidade que regula isso, como é o caso da água. A água, a gente até pode estar quinze dias sem comer, a água, se tivermos três dias sem beber, morremos. Portanto, a água é um bem essencial, sem água, quando, quando se fala nas explorações de Marte, a preocupação é descobrir água, portanto, a gente sem água não vive e a água pode estar realmente poluída, e pode ser um mal. Eu estive no Brasil há muitos anos, e no Brasil não se bebe água pela torneira, é tudo água engarrafada. A gente aqui, os meus primos vinham cá, a gente "queres um copo de água?" íamos à torneira abrir e eles ficavam assim um bocado esquisitos. Até quando é que nós temos confiança de que podemos beber a água da torneira...

**J.**: A água engarrafada também não nos dá garantias.

**C.**: A água engarrafada também não nos dá garantias de que a água seja realmente boa. A gente não sabe. Ou seja, passa tudo pela confiança que a gente tem nas entidades que supervisionam isto. Agora, podemos confiar ou não. Isso aí já é outra história.

**M**: Podemos confiar nas, nas entidades, não é? Mas...

**C.**: Por enquanto, eu confio.

**M**: O que vocês estão a dizer é que não confiam especialmente *(...hã...)* ou desconfiam dos efeitos que as substâncias podem ter no corpo, não é? Já se falou do cancro, falamos de alergias, que mais é que nos pode acontecer, quais as preocupações associadas em termos de saúde, efeitos na saúde, das substâncias?

**J.**: Ontem penso que passou uma notícia que era extremamente interessante, tinha a ver com, com a quantidade de antibióticos que circulam *(...hã...)* circulam na água, nos rios, etc. E a resistência, a resistência às bactérias vai ser um dos graves problemas de, dos próximos anos. Acho que muita gente já percebeu isso. E nós estamos cada vez mais com, com menos resistências, ou seja, não é uma questão de tomarmos ou não tomarmos antibióticos, ou termos tomado *(...hã...)* muitos ou poucos antibióticos, a questão é que eles estão por aí disseminados e que nós estamos a ter contacto com eles e, portanto, *(...hã...)* ouvi alguns casos recentes *(...hã...)* de hospitais em que as novas, os tais super-antibióticos que vêm combater bactérias multirresistentes, que eles próprios já não estão a funcionar *(...hã...)* e, portanto, esse é um problema que poderá ser muito grave no futuro.

**M**: Ia dizer alguma coisa.

(22:35)**H.**: *(...hã...)* Na minha opinião às vezes também há aqui um, um bocadinho de falta de equilíbrio na percepção de, no caso das doenças, por exemplo, porque, se calhar, há muitos anos atrás, ou se morria pela ponta de uma lança ou se morria de causa natural e esta causa natural englobava tudo, englobava cancros, englobava doenças de todos os tipos que, neste momento, são muito mais escrutinadas e sabe-se exactamente do que é que uma pessoa morreu. Portanto, às vezes é também preciso ter aqui algum, algum equilíbrio de, há assim tantos mais casos ou os casos são mais visíveis? *(...hã...)* Relativamente à água que, que eu falei. *(...hã...)* A minha preocupação maior não se prende tanto no consumo directo, que acredito que seja bem legislado e bem controlado, mas muito nos consumos indirectos, isto é, a água que é usada para regar as plantações que nós depois comemos não é assim tão escrutinada, muitas vezes é retirada directamente do rio ou de outro curso de água *(...hã...)* e também em Portugal há uma percentagem maior do que seria de, de esperar de pessoas que ainda usam exclusivamente água de furos artesanais.

**M**: OK. OK.

(23:56)**B.**: Os alimentos estão hoje, eu acho que isso está, quer dizer, está demonstrado, estão muito mais processados, a alimentação está muito mais processada *(...hã...)* e há uma ligação, por exemplo, como o, como o I. disse noutro contexto, quer dizer, os estudos ainda não são propriamente muito elucidativos a esse respeito, mas há muita coisa, demasiada coisa por estudar, o efeito deste conservante, daquele aromatizante, daquele corante, etc., mas *(...hã...)* isso está associado, nomeadamente, a uma coisa... Eu aqui há uns dias ouvi uma entrevista ao professor Sobrinho Simões, e ele dizia (...) "vai-se morrer menos de cancro, porque ele vai ser mais curável, mas vai haver mais pessoas com cancro", ou seja *(...hã...)* e vai, por exemplo, e vai haver, e nós vamos assistir, digamos, ao, ao cancro, enquanto não doença terminal, mas doença crónica. E, naturalmente, esse muitas formas de cancro estarão provavelmente associadas *(...hã...)* também, poderão estar associadas *(...hã...)* ao tipo de alimentação que estamos a ter. Eu vejo, eu vejo por mim, eu acho que sou um péssimo exemplo, desde manhã, é, é, muitas das coisas que eu estou a comer que são, enfim, (...) porcaria, é muito considerável. Tudo, por exemplo, que vem das máquinas que nós temos, a faculdade tem várias máquinas, e as máquinas não têm peças de fruta, têm barras de chocolate, têm... etc.

**J.**: Isso é o que aparece por todo o lado. Eu gostava de pegar aqui na questão da confiança só para contrapor um pouco. É que se nós formos pensar no exemplo do tabaco, por exemplo, *(...hã...)* o tabaco, o consumo do tabaco nos anos 30, 40, 50, era fomentado, e ninguém dizia que era prejudicial à saúde. Hoje já se sabe que a indústria sabia disso e durante anos e anos isso foi, isso foi abafado, só quando houve evidência, de facto, que era prejudicial é que se assumiu esse carácter, portanto, se calhar passamos aqui, se calhar hoje com a questão da alimentação. A questão dos animais é uma questão interessante, porque os veterinários aquilo que defendem é que, é o aumento da esperança de vida dos animais, também, não é, e a verdade é que existem cada vez mais animais com, com, com cancros, *(...hã...)* e voltando àquilo que eu estava a dizer no início da, da agroindústria, eu fiquei absolutamente chocado por saber, por exemplo, que penas de frango são utilizadas para fazer rações para animal *(...hã...)* e, portanto, quando se pensa nisso, se calhar, conseguimos explicar um bocadinho o que é que se passa com, com os animais. Obviamente que não há nenhuma evidência sobre isso, não é, mas se calhar dá que pensar.

**B.**: Na criação em cadeia desses animais, que estão confinados a espaços exímios, mal se podem mexer, toda essa alimentação está pejada de antibióticos. Toda, toda a cadeia alimentar está...

**J.**: E ainda existe o peixe, essas coisas.

**L.**: Ainda no outro dia ouvi uma conversa, um senhor que cria cabritos para comer, e o cabrito estava doente, e deu-lhe uma injeção, e aquele cabrito não podia ser comido no prazo de 15 dias, e ele foi vendido na semana a seguir, e disseram-lhe "não podes fazer isso", " quero lá saber disso, não sou eu que o vou comer" e depois disseram-lhe assim "de certeza que a pessoa vai comer, vai ter diarreia, vai fazer mal, porque vai comer, vai comer aquele, o remédio que ele tinha lá”. Por exemplo, eu tenho casa ali perto de Alcobaça e há lá uma vacaria. Eu eu vejo que o senhor andava sempre lá à volta dos bois a espetar e outras coisas. Por exemplo, a mim, a mim proibiram-me não, proibiram, mas disseram-me para eu não comer feijão branco enlatado e ervilhas enlatadas. Congeladas posso comer, frescas, isso tudo, menos em lata. Ervilhas e feijão branco. Só o feijão branco, o outro não tem problemas. Portanto, não sei o que é que tem lá no feijão branco, deve ter lá outra, outro químico qualquer e como as ervilhas, para ficaram verdinhas ou qualquer coisa, não sei, que eu depois também não perguntei, mas foi duas coisas que me proibiram de comer.

**M**: Deixem-me ver se eu consigo resumir os aspectos de saúde que vos preocupam. Cancro, resistências aos antibióticos, não é, alergias, *(...hã...)* coisas mais do foro gastro, gastro, portanto, diarreias e por aí fora, houve alguém que falou de reprodução, não é, como é que as coisas correm para o futuro e tal. Mais alguma coisa? *(...hã...)* Mais alguma coisa que vocês identificam associado à exposição que vão tendo?

**D.**: A parte respiratória.

**M**: OK.

**D.**: Não só com os poluentes como até a própria Natureza. Eu constato que há determinadas árvores, nomeadamente os plátanos e as tílias, que provocam imensa alergia.

**B.**: Desculpem estar a falar tantas vezes, mas vou já calar-me. Só para dar uma achega para, para corroborar aquilo que disse o J. *(...hã...)* Há um número que, enfim, uma percentagem assustadora de norte americanos que tem, que está viciada em opiáceos porque justamente as autoridades, (...) enfim, o INFARMED lá do sítio, ou os INFARMEDs lá do sítio, disseram que era seguros e não causam dependência e por aí fora, quer dizer, é assustador. Nesse aspecto é assustador.

**C.**: Isso há muitos interesses [Claro, claro, claro.] Aliás, toda esta fabricação de comidas que têm que ser vendáveis, a rapidez com que têm de ser vendáveis, *(...hã...)* injectar hormonas no gado para o gado crescer, para se vender, tudo isto tem por trás sempre um aspecto económico, sempre, sempre, sempre...

**I.**: Saúde mental.

**M**: Saúde mental? Porque é que dizes?

**I.**: Não se vêem muitos estudos que analisem *(...hã...)* actualmente os problemas que podem ser causados até na saúde mental das pessoas pela absorção de certos componentes. Eu digo isto porquê, porque também apareceram outros estudos que apontam para uma certa dose de demência no Império Romano *(...hã...)* como causa próxima do consumo das bebidas em copos de chumbo. E o aumento do chumbo no organismo, e que foi detectada naqueles, naquelas ossadas daquelas pessoas, poderia explicar esse tipo de, de comportamentos.

**M**: Demência. OK. OK. Muito bem. *(...hã...)* Portanto, vocês falam, já falaram de muitos agentes químicos, e já falaram muito de problemas de saúde. Bela confusão, não é? E agora, a questão aqui é, se vocês pensarem em vias de absorção, o ar, a água, a alimentação, o contacto com a terra, enfim, de tudo, de onde é que vem o maior perigo *(...hã...)* o que é que vos preocupa mais, agora das formas de, de absorção.

**D.**: Para mim é a alimentação.

**M**: Alimentação.

**L.**: A alimentação e o ar, a poluição atmosférica.

**M**: E o ar? A alimentação e o ar. Poluição atmosférica. F., dizes que não?

(31:26)**F.**: Não, digo que é tudo por igual, porque nós absorvemos todas essas coisas e consumimos todas essas coisas todos os dias, de uma forma directa ou indirecta. Não conseguiria estruturas em prioridades.

**M**: OK. OK. Outros pareceres?

**I.**: Depende muito do organismo das pessoas também. *(...hã...)* Por exemplo, quando tenho uma ferida e estou a tomar banho, *(...hã...)* e tenho que me lavar com um desses modernos produtos de higiene que nós usamos, eu penso muitas vezes se não haverá perigo de absorver por aquela ferida aberta, que a pele não tem tantas protecções, directamente certas coisas que, de outra forma, estaria imune a elas.

**M**: Também depende da pessoa e da condição da pessoa.

**I.**: Sim.

**M**: OK.

**H.**: Falamos, já mencionados aqui *(...hã...)* e especialmente nesta altura que se aproxima, muito o contacto, por exemplo, nos banhos que, pronto, toda a gente passa, vai para as praias e a qualidade da água não é monitorizada, por exemplo, nas praias da linha de Cascais e as nossas mais próximas, não poderemos dizer que será muito melhor.

**M**: OK. Muito bem. *(...hã...)* Bem, eu, eu agora mudava um pouco a agulha da conversa e íamos para aquilo que é a biomonitorização humana, ou seja, para falar dela, de alguma maneira, não é, o que é que é a biomonitorização humana quando, quando, quando nos dão este chavão, não é, este palavrão, isto diz-nos o quê, *(...hã...)* o que é que vos passa pela cabeça, o que é que é isto de biomonitorização humana, o que é se faz nesta área?

**L.**: Não faço ideia.

**I.**: Retirar amostras de pessoas voluntárias e analisar essas amostras, de cabelo, de sangue, e de pele, etc., se há presença de, de determinadas coisas que se querem despistar.

**J.**: Nós já assinámos para fazer isso no final *[riso de vários participantes]*

**M**: OK.

**C.**: Eu penso que é os sinais de alerta de que algo está mal e que é preciso controlar e daí estes estudos, porque isto não se fazia dantes. Como o tabaco que se fumava, até se incentivava, uma pessoa era homem era a fumar, eu quando era miúdo, uma pessoa fumar é que era homem, não é, felizmente já deixei de fumar há trinta e tal anos, portanto, deixei o tabaco, mas, e esse alerta não é só para, acho eu, não é só para poupar dinheiro ao Estado no tratamento das pessoas que fumam. É para proteger as pessoas. Eu penso que estes estudos de biomonitorização é os sinais de alerta a tocarem, as sirenes a tocarem, de que algo está mal e que tem que se começar a travar, tem que se começar a ter cuidado, *(...hã...)* fala-se muito de as alterações climáticas, etc. O planeta Terra sempre teve alterações climáticas, não é de agora, penso que isto deve ser natural *(...hã...)* o que há é a rapidez com isso tudo. Eu penso que a evolução da, da ciência e investigação *(...hã...)* dá-nos muito mais informação do que a gente tinha dantes, eu digo a gente, o ser humano, portanto, tinha dantes, nos séculos passados, não havia tanta informação e, portanto, a gente não estava alertada. Aquilo que há bocado disso um pouco, andávamos um pouco na ignorância.

**M**: Portanto, o C. já está a introduzir aqui uma coisa que é, porque é que se faz, não é? Portanto... um sinal dos tempos.

**C.**: Porque é que se faz. Exactamente. Isto começa a soar alertas de que algo está mal e que temos de ter cuidado.

**M**: OK. OK. E ali o I. já descreveu um pouco uma coisa importante, que é esta coisa de recolher amostras, não é, apanhar as pessoas *(...hã...)* OK, mas qual é, qual é o objectivo, o que é que queremos saber ao fazer esta coisa que é biomonitorizar...

**B.**: Posso abrir a janela.

**M**: Podes.

**B.**: [IMPERCEPTÍVEL] *[riso de vários participantes]*

**M**: Agora baralhaste-me. *(...hã...)* *[riso do próprio]* Posso abrir, mas ficou sentado *[riso de vários participantes]* [IMPERCEPTÍVEL] OK. O que é que é então a biomonitorização humana?

**B.**: Monitorizar-se significa controlar-se, eu diria que vão ser feitos estudos sobre *(...hã...)* um químico que incide sobre uma determinada amostra da população e que pretendem ver que efeitos têm isto, A, B ou C, o produto A, B ou C, ou, por exemplo, sobre essa amostra da população em *x* tempo, de que maneira...

**M**: Acompanhar as pessoas.

**B.**: Sim, monitorizar, é um pouco isso, acompanhar, controlar, *(...hã...)* e medir também.

**M**: OK. Mais ideias?

**D.**: Eu acho que até a biomonitorização nós fazemos a nós próprios.

**M**: Ora, esse é um aspecto interessante. Cada um de nós faz monitorização, não é, esse é um aspecto interessante. *(...hã...)* Qual é a diferença entre isso e fazer biomonitorização humana, imaginem, aqui na faculdade *(...hã...)* há aqui uma série de pessoas a pensar no assunto *(...hã...)* Qual é a diferença? Estamos a falar do... Quais são as diferenças entre um e outro?

**D.**: Universo, universo, digamos que, a que vocês fazem é no âmbito, no âmbito universal, *(...hã...)* enquanto que cada um de nós faz de si próprio, ou de quem o rodeia, mas essencialmente de si próprio.

**B.**: Quando faço biomonitorização sobre mim próprio, quer dizer, a mim próprio, a meu respeito, faço batota, vocês têm [IMPERCEPTÍVEL] *[riso de vários participantes]*

**D.**: Ah, mas eu não faço batota.

**C.**: Eu penso que a vossa biomonitorização, tem sempre uma base científica, enquanto que a nossa é uma base empírica, ou seja, sinto-me bem, sinto-me mal, fez-me mal, fez-me bem.

**M**: Mas esse aspecto é muito interessante e não estava previsto aqui na conversa, mas isso é que torna estas conversas engraçadas. Como é que vocês fazem biomonitorização humana? O que é que isso de se fazer, no indivíduo, fazer a biomonitorização humana?

**C.**: Eu penso que é as consequências de alguma coisa que a gente fez e que nos sentimos mal, porque se nos sentimos bem, eu acho que não fazemos biomonitorização.

**M**: Quando se sentem mal, fazem.

**D.**: Não, mas eu ainda tenho uma outra situação, por exemplo, se eu tenho na minha família antecedentes *(...hã...)* muito próximos, *(...hã...)* de pessoas com cancro, eu tento ter uma alimentação saudável, ainda por cima cancro no aparelho *(...hã...)* digestivo, eu tento ter uma alimentação mais saudável possível porque, potencialmente, corro o risco de contrair a doença. Se tivesse problemas respiratórios, se calhar incidiria mais sobre a parte respiratória, ou seja, eu incido pela parte mais frágil da minha pessoa.

**M**: Concedem dar exemplos concretos do que é que é biomonitorizar-nos em nós próprios?

**B.**: Eu, por exemplo [IMPERCEPTÍVEL] *[riso de vários participantes]*

**I.**: Não, não. É, por exemplo, a questão da, da palpação mamária que as senhoras podem fazer para detectarem primariamente sintomas de caroços ou de coisas parecidas que possam, até é aconselhado pelos médicos. É uma técnica,

**M**: F.

**F.**: *(...hã...)* Pronto, nós ainda não chegámos bem conceptualmente o que é que é biomonitorização humana, mas se pensarmos no que é que nós fazemos na nossa vida diariamente, fazemos um bocadinho isso, sempre que temos alguma mancha na pele que não sabemos de onde é que vem, vamos ver, se temos algum desconforto abdominal, torácico, onde quer que seja, vamos ver se isso pode entrar ou não para o conceito de biomonitorização humana, não sei bem, porque não sei bem qual é o conceito.

**M**: OK. Mais ideias?

**H.**: Eu, por exemplo, *(...hã...)* se calhar, mais, quase um teste que uma pessoa faz que, por exemplo, eu, sempre que vou à *(...hã...)* localidade onde nasci que, há uns tempos, sempre, sempre que de lá vinha, vinha mal-disposto, com, com problemas de saúde, e então comecei a, primeiro, a cortar este alimento que deixei de comer, normalmente coisas produzidas localmente, depois este alimento, depois este alimento, até que cheguei a alguma conclusão do que é que realmente me estava a fazer mal.

**M**: Fazendo testes, fazendo testes. Muito bem. OK.

(39:47)**K.**: A informação, não é, que vamos colhendo, não é, que vamos ouvindo e lendo, e nã, nã, nã, *(...hã...)* também é importante, não é, porque nos dá alertas para coisas que anteriormente não esperávamos, não é, e dá-nos atenção, porque ouvimos determinada notícia, não é, ou opinião, não é, ficamos mais focados nessa, nessa questão, não é?

**M**: Portando, aprendemos e depois biomonitorizamo-nos, é isso?

**K.**: Sim.

**M**: OK. Mais algum, mais alguma, algum exemplo de biomonitorização?

**I.**: Bom, já agora não sei se o que vou dizer é uma grande asneira, mas quando a pessoa *(...hã...)* se preocupa em sugerir ao próprio médico. "Sr. Doutor, eu gostava que me fizesse análises a isto ou àquilo, por alguma coisa empírica ou sintoma que tenha", poderá estar a contribuir para isso, digo eu.

**M**: Pronto, isto é à escala do indivíduo, não é? Obviamente biomonitorização humana, aquilo que andamos a falar neste contexto, tem mais a ver com o fazer investigação, como alguém disse, penso que foi a D. *(...hã...)*, fazer isto com várias pessoas, é uma questão de escala, não é? *(...hã...)* No vosso imaginário, se vocês pensassem em tipo de cientistas, com aquele ar assim louco, não é, tipo o Ricardo *[riso de vários participantes]* *(...hã...)* Vocês pensam em quê, quem é que anda a estudar biomonitorização? Que tipo de cientistas? Que tipo de investigadores?

**H.**: Engenharia bioquímica.

**D.**: Os biólogos. Julgo que também médicos. Os psicólogos.

**M**: OK. Mais.

**H.**: Nutricionistas.

**L.**: A medicina molecular, também.

**M**: Medicina molecular, nutricionistas. Portanto, uma gama bastante [diversificada] alargada de investigadores, não é, de áreas. Muito bem. Eu agora, eu se calhar, passava para a apresentação.

(41:54)**CM**: Posso só...

**M**: Força.

**CM**: Quando pensam nesses investigadores, loucos, pensam *(...hã...)* em que ambientes de trabalho é que pensam, é que imaginam, fantasiam esses investigadores a trabalhar, no laboratório, *(...hã...)* [no ISAMB] sim, mas em que ambientes, em que ambientes de trabalho.

**J.**: Dependendo também do âmbito de, do estudo, não falámos aqui dos veterinários, também provavelmente também têm, também estão envolvidos, por causa da parte animal *(...hã...)* mas pode ser ao ar livre, em determinadas zonas, não é, *(...hã...)* pode ser no laboratório, obviamente, que também tem essa cultura.

**CM**: Mas quer dizer, pensam nos cientistas com o, com a proveta e com o medidor ou também pensam naqueles cientistas, cientistas sociais, *(...hã...)* como os psicólogos *[riso de vários participantes]*, os sociólogos...

**I.**: Eu, eu na minha imaginação, vejo uma equipa multidisciplinar e por isso vejo desde quem faça colheitas no exterior *(...hã...)* à pessoa que vai fazer as colheitas nos seres humanos, que traz para o laboratório, a pessoa que faz análise no laboratório, pode ser um bioquímico, portanto, é uma equipa completa e não uma única pessoa. Já agora, posso fazer aqui um pequeno à parte. Ainda sobre este tema, eu estava-me a esquecer há pouco de dizer uma coisa, algo que seria interessante fazer-se e que eu não sei se já está a ser feito, era acompanhar o processo num indivíduo ao longo do tempo, ou seja, *(...hã...)* um estudo que inicialmente começasse com bebés, ou com o leite materno ou uma coisa qualquer, e esses meninos que fossem acompanhados, por exemplo, nova colheita de material aos 10 anos, nova colheita aos 20, nova colheita aos 30, sobretudo pessoas num universo de pessoas que vivessem naquela zona, que não, não se dispersassem muito pelo mundo, para que os dados obtidos, sobretudo se quisessem investigar o efeito de alguma fábrica na zona ou coisa parecida.

**J.**: Eu acho que isso já é feito com algumas, pelo menos nalgumas, portanto, algumas indústrias. *(...hã...)* Postes de alta tensão, pessoas que vivem em zonas de postes de alta tensão acho que são normalmente controladas, ou outro tipo de indústrias que tenham algum controle mais importante ao longo da vida, acho eu.

**M**: OK. Mais alguma ideia? Para já? Não? Pronto. Eu, se calhar, não vos vou chatear muito, mas só para vos falar um pouco sobre o nosso projecto, para depois conversarmos um pouco mais sobre isto.

(45:06) [Início da apresentação do projecto]

(53:43) [Fim da apresentação do projecto]

**J.**: Posso fazer só duas perguntas, já agora? É que este, existe uma monitorização que está definida ao longo dos anos, não é, de 5 em 5, quer dizer, se calhar, de 5 em 5, não é [de 5 em 5], existe uma série, uma panóplia grande depois de produtos que têm mesmo, está instituído quais são, a nível europeu, Estados, ou não.

**CM**: A nível europeu *(...hã...)* [ou Estado a Estado] Cada Estado faz uma monitorização própria, eu penso que há directivas comunitárias que, para determinados compostos, há obrigatoriamente uma monitorização, por exemplo, ao nível das incineradoras, há uma directiva comunitária que obriga a que determinados compostos, como, sei lá, o mercúrio, enfim, uma série de compostos químicos, sejam de facto monitorizados com, com alguma regularidade, e que sejam reportados para a Agência Europeia do Ambiente.

**J.**: E a maior parte destes compostos são conhecidos os limites *(...hã...)* nocivos para, para a saúde humana ou não?

**CM**: Quer dizer, a maior parte, nós estamos a discutir a maior parte, a maior parte que nós conhecíamos é a menor parte, quer dizer, a maior não conhecemos.

**M**: Vão surgindo novos.

**CM**: Claro. Mas daqueles que nós vamos conhecendo, nós conhecemos os limites.

**I.**: A., relativamente às radiações electromagnéticas, há uma medida, que é o mV por metro.

**M**: OK. E há-de haver um nível...

**I.**: Há limites, há limites, há limites. Até porque *(...hã...)* os malefícios da exposição variam de acordo com, não só com a intensidade, mas com a própria frequência. Há frequências que causam, estão comprovados os efeitos, causa-efeito.

**M**: Mas, melhor, um aspecto que é importante, é, *(...hã...)* haverá a frequência, haverá *(...hã...)* o nível de exposição, mas também a duração da exposição. [A duração] A biomonitorização preocupa-se muito com isto que é: o que é que acontece quando eu sou exposto a uma coisa que até está dentro do normal, não faz mal, mas o que é que acontece quando eu sou exposto a isto a 20 anos *(...hã...)* e isso sabe-se pouco. Em resposta ao J., há muita coisa que vamos percebendo, não é, à medida em que... e em rigor, *(...hã...)* não há capacidade para estudar tudo ao mesmo tempo, portanto, isto tem que ter uma agenda, não é. OK.

**CM**: Estou-me a lembrar agora, por exemplo, em relação a esta substância em concreto, foi, foi, foi muito estudada, por exemplo, e ela está muito presente nos sedimentos do rio Trancão, ou seja, por essa via da exposição à água dos rios *(...hã...)* esta substância entra no nosso organismo, os sedimentos do rio Trancão estão extremamente contaminados com, com esta substância.

**I.**: Com esta e com outras, são os chamados poluentes emergentes na água que, inclusivamente, provocam alterações fisiológicas nos animais, nos peixes sobretudo - essa parte eu estou mais dentro do assunto - e em outra oportunidade podemos falar sobre esse assunto.

**CM**: Às vezes as vias não são directas, são, são, bastante indirectas.

**M**: Já temos o pano de fundo, certo? E agora a minha pergunta é: olhando para isto *(...hã...)* e com estes elementos para explicar melhor o que é que é a biomonitorização, já está um pouco mais visto, *(...hã...)* de que formas é que acham que a biomonitorização funciona para vocês, agora em termos mais práticos?

**B.**: Mas individualmente?

**M**: Se pensarmos em termos, OK, isto, do que ouvi isto, para mim, é relevante porque?

**D.**: Conforta-me. Saber que há alguém que está a estudar algo que me pode prejudicar.

**C.**: Exactamente, é isso mesmo... Saber que, eu não percebo nada disto, mas alguém percebe e está preocupado. Isso deixa-me mais descansado.

**M**: Por um lado, aumenta a tranquilidade, o conforto. OK. Mais?

**C.**: Ou seja, já há, já há alarmes para estes malefícios e alguém está preocupado com isso.

**D.**: E alguém vai obter determinado resultado que irá, *(...hã...)* a quem de direito, nomeadamente à parte política, para tomarem medidas sobre esses aspectos.

**B.**: Mas eu não sou tão optimista. *[riso de vários participantes]*

**D.**: Ai eu sou!

**C.**: Não, com a parte política eu não estou muito optimista *[riso de vários participantes]*

**B.**: O problema é esse. Na ciência...

**C.**: Agora, na parte dos cientistas, não.

**B.**: No meu caso, na ciência, isso é uma coisa obviamente, quer dizer, é inegável. Não se, pronto, de uma forma extraordinariamente, grosseiramente simplista, quando se [IMPERCEPTÍVEL] A questão não é essa, a questão do exemplo do tabaco, é um excelente exemplo. Eu cada vez que arquivo um, um, um artigo científico no repositório, às vezes vou ver os conflitos de, de interesse e fico assustadíssimo. Se a, se a farmacêutica A, B ou C ou X, Y ou Z patrocinou este estudo, quer dizer, para dizer que o fármaco não sei quê é perfeitamente inócuo, mas o fármaco, quer dizer, é fabricado por tal empresa... Mas depois, claro que diz, não, não, mas a farmacêutica em nada influenciou os resultados finais destes estudos. Claro...

**J.**: Objectiva ou subjectivamente, se calhar, até pode ser, não é.

**K.**: Pode ser também inquietante, não é?

**M**: Inquietante. Por um lado, conforta, por outro lado é inquietante. Inquietante de que maneira?

**K.**: Põe-nos alerta para coisas *(...hã...)* que, que estas coisas que eles estão aqui a dizer, não é. Se um estudo é patrocinado por uma determinada farmacêutica, etc., não é, como isso pode enviesar...

**H.**: Eu acho que, descobrir um bocadinho a causa-efeito, *(...hã...)* acho que, mais assustador do que, por exemplo, estar doente, é estar doente sem se saber bem porquê nem com o quê. *(...hã...)* E havendo, por exemplo isto, *(...hã...)* havendo uma monitorização de, de certos poluentes ou outros, e perceber o que é que isso produz depois na população, faz com que também uma pessoa quando se sente mal ou se sente doente, não é tal coisa de morte natural, morre de alguma coisa em específico, então, se calhar, sentimo-nos com algum poder de (...) poder evitar algumas coisas, por um lado, e não podendo evitar, saber que se calhar já se está numa direcção de investigação que nos permite salvar, entre aspas.

**M**: OK.

**D.**: Eu julgo que deve, após estes estudos, deve ser feita uma divulgação, para que, geral, para que as próprias pessoas tenham conhecimentos que os estudos estão a ser feitos e até que ponto não poderão pressionar a parte política que as coisas sejam aplicadas.

**M**: OK. OK.

**D.**: Acho que é fundamental a divulgação desses estudos.

**M**: Portanto, divulgação quer para o público quer para os decisores políticos, é isso?

**D.**: É.

**I.**: A mim também há outra coisa que me conforta. Ao descobrirem certas causas através desses estudos, abrimos as portas à medicina e à investigação começarem, independentemente das restrições que virão com a lei, etc., começarem também a abrirem novas frentes de estudo para tentar perceber se aquela doença que se atribuía só a isto ou se pode atribuir àquilo, se não podemos começar a trabalhar *(...hã...)* na prevenção dessas doenças evitando certos tipos de vida, certas práticas, certas exposições. Ainda a propósito disto. Nós estamos a focar estes estudos só na Europa, mas não nos podemos esquecer que o planeta é muito maior do que a Europa e que coisas que acontecem fora da Europa podem-nos influenciar individualmente de duas formas: ou quando vamos de férias a algum sítio, ou então quando, estava-me a lembrar agora na agricultura, o que é que me interessa a mim tratar a mosca-branca com químicos se os meus vizinhos não tratam as árvores deles. Eu mato a mosca-branca nas minhas árvores, a mosca, os, os parasitas das minhas árvores morreram neste momento, mas daí a uma semana os descendentes dos parasitas das árvores dos meus vizinhos vêm infestar a minha árvore também.

**J.**: Se nós pensarmos num exemplo simples, que é o da fruta, e quando vamos ao supermercado, e se formos ver a proveniência da fruta...

**I.**: Lá está.

(1:02:32)**E.**: Mas também há, há uma outra parte que também me preocupa. Faz-me todo o sentido a evolução da ciência e o grupo de estudos que tem sido feito, mas em termos de divulgação, eu percebo que essas questões tenham que ser passadas para o público em geral, mas isso causa bastante alarmismo, em toda a gente. Toda a gente liga as notícias e há o estudo que foi divulgado que isto faz mal a não-sei-quê. E porque antes o salmão era um peixe fantástico e que tinha imenso ómega-3 e que agora o salmão é péssimo e não podemos comer salmão.

**J.**: Se for selvagem, continuamos. *[riso de vários participantes]*

**B.**: Desculpem interromper. Isso é absolutamente certo, isto é um aspecto que é *trendy*, agora, quer dizer, está na moda fazer estudos sobre isto, isto, isto e isto, e depois os estudos contradizem-se.

**E.**: Exacto. É isso.

**B.**: O leite, o leite já foi o nosso melhor amigo, já foi o nosso pior inimigo, já foi tudo. Um indivíduo já não sabe para onde é que se há-de virar, se for baseado apenas em, naquilo que lê, em certos órgãos de informação, quer dizer, agora, bebo ou não bebo leite.

**E.**: E aquilo que o A. estava a dizer que *(...hã...)* muitos grupos de cientistas estão a fazer muitas vezes os mesmos estudos, basicamente a ciência está dar um próprio tiro no pé, porque um grupo de cientistas descobre uma coisa, outro grupo descobre outra, e às tantas divulgam-se os dois resultados e ficamos no quê, o salmão afinal é bom ou é mau? E não sabemos às tantas onde é que andamos.

**H.**: Às vezes falta um bocadinho de contextualização no que se divulga. Por exemplo, *(...hã...)* e isso é um bocadinho culpa nossa, um bocadinho de todos *(...hã...)* O título que sai às vezes pode ser o poluente *x*, ou a partícula *x*, aumentou dez vezes na atmosfera, mas estamos a falar de uma parte de um milhão para dez, se calhar isso na realidade não faz absolutamente nada, *(...hã...)* não muda nada em nosso, no nosso dia-a-dia e em nós. Só que, de repente, cria-se um alarmismo porque aumentou dez vezes, mas aumentou dez vezes se calhar irrelevantemente, não é?

**C.**: Mas isso é um pouco aquilo que eu disse no começo. Às vezes a ignorância até é um bocado bom, porque a gente não sabe se o salmão nos vai matar ou não, mas é bom ou não é bom, sabe-nos bem ou não, a gente tem prazer quando come o salmão ou não? Então podemos comê-lo.

**E.**: Perdoa-me o mal que faz pelo que bem que sabe *[riso de vários participantes]*

**C.**: Porque às tantas o estudo que diz que o salmão faz mal e o leite e coisas dessas, se calhar não faz nada mal, há um outro componente à parte, exterior, que é esse que é sim é que pode provocar alguma coisa mal em conjunto com, com as outras substâncias. Não sei. [Já aqui foi falado, já aqui foi falado...] Eu, pois, eu gosto, não é completamente ignorante, que isso é um bocado mal, não é, mas gosto muitas vezes de me abstrair desses, desses resultados, isso agora mal, é a intolerância à lactose, é não-sei-quê que está na moda, agora toda a gente, como dizia o Professor M., somos o único animal que em adulto bebe leite e então, também somos o único que come bacalhau à Brás *[riso de vários participantes]* como disse o Professor M. na entrevista do i, do jornal. (...) Faz muito mal, então somos o único animal que bebe leite em adulto, mas também somos o único que comemos bacalhau à Brás, e então?

**H.**: Eu digo isto da contextualização porque, agora, puxando aqui dois minuti, dois segundinhos à minha área *(...hã...)* Quando se fala, por exemplo, que este ano já morreram seiscentas vezes mais pessoas em desastres de aviação do que no ano passado, embora seja tecnicamente verdade, é porque no ano passado não morreu ninguém e este ano morreram seiscentas pessoas.

**I.**: E não só. No ano passado havia menos aviões no ar do que há este.

**H.**: Pois, é aquela coisa. Todos, todos nós ouvimos constantemente que estão cada vez mais espécies em vias de extinção e assim, mas também cada vez há mais espécies descobertas. *(...hã...)* Às vezes é preciso esse contexto.

**M**: É preciso contexto. OK. Muito bem. Então, mas em que é que ficamos? *(...hã...)* Porque, agora, o projecto vai terminar daqui a dois anos, não é, e uma das coisas como se disse é criarmos regras comuns a todos, tanto quanto possível, e uma regra é, como é que nós comunicamos, como é que devemos comunicar melhor os resultados na área da biomonitorização, é, é contactar o público, como vocês disseram, divulgar resultados, [Primeiro, contextualizar.] ...a dizer que isto é importante, por outro lado, vocês disseram, olhem, isto pode criar alarme, como é que...

**D.**: Eu prefiro saber do que estar na ignorância.

**L.**: Eu prefiro saber

**M**: Prefere saber?

**L.**: Sim.

**D.**: Eu também.

**L.**: Eu prefiro saber. Portanto, o salmão. Há o selvagem, pronto, é muito caro, nós não podemos comprar. Há outro que é mais barato que pode fazer mal, mas se nós comermos uma postita, menos de metade, entre o salmão e as salsichas, prefiro o salmão.

**D.**: Pelo menos sabemos que existe aquele problema. A opção é nossa.

**L.**: Exactamente.

**C.**: Eu gostava de saber que os cientistas têm a percepção disso e evitam que a gente coma coisas que não devemos.

**M**: Aliás, isto é interessante.

**L.**: Eles não evitam, podem é avisar.

**C.**: Eu prefiro confiar. Se a gente não confiar em ninguém a coisa é má.

**M**: A perspectiva do C. é diferente, não é, portanto, a comunicação deve ser mais, imaginem, os decisores políticos, os decisores... [C.: Os políticos... / D.: Não só.], OK, os decisores de alguma coisa e eles hão-de controlar, é isso? Não é? É, é essa a postura.

**I.**: A., nós temos um problema na nossa sociedade que é premente, e que se está a agravar. A comunicação social não nos está a prestar um bom serviço. [É um negócio.] Não está a prestar um bom serviço porquê? Porque está muito baseada, é muito economicista, está muito baseada no lucro [Exactamente.] e, e como está baseada no lucro, está a atropelar as suas próprias regras e aquilo que se aprende nas faculdades em comunicação. *(...hã...)* O bom exemplo que foi dado dos títulos, por exemplo, [Dos?] títulos das notícias, porque hoje *(...hã...)* somos bombardeados com informação, e muitas vezes não passamos do título. A contextualização que vem a seguir também não é bem feita porque as pessoas são pressionadas para escrever numa determinada forma porque essa forma é que atrai mais as atenções e que chama mais gente. Aqui devia de haver uma fonte fidedigna de transmissão do conhecimento nestas áreas que estivesse *(...hã...)* acessível às duas partes: ao consumidor final, que somos nós, e a quem quer dar a notícia. E o consumidor final via que a notícia tinha vindo do sítio tal e podia ler a notícia original. A maior parte das pessoas não faria isso, mas quem, quem quisesse, podia fazer isso, e quem quisesse ignorar as notícias da comunicação social, ia directamente à fonte. Eu penso que isto poderia ser uma solução, talvez não seja a melhor...

**C.**: Há um projecto em curso para isso, há um projecto que...

**E.**: A questão é que a maioria das pessoas tem acesso à informação através da comunicação social, por exemplo, enquanto estão a jantar e a ver as notícias.

**I.**: Por preguiça.

**E.**: Por preguiça ou por falta de meios para terem acesso a outro tipo de, de informações.

**I.**: Eu, por exemplo, ainda oiço muito as emissões em onda curta, quando posso, quando tenho tempo, porque vou buscar informação (...) à fonte e de uma forma completamente diferente o que ela nos é transmitida depois mais tarde no telejornal.

**E.**: A questão é que eu acho que a informação a ser transmitida, por exemplo, no telejornal, deveria ser validada previamente pela pessoa que a quer transmitir para saber se a mensagem está a ser passada...

**J.**: Supostamente isso deveria ser o trabalho do jornalista fazer essa validação, faz parte de...

**B.**: Essa validação é feita, os critérios não são é científicos, os critérios são os do bruaá, os do espalhafato.

**J.**: Mas a própria, mas a própria informação que chega à comunicação social se calhar também muitas vezes não é a mais correcta, quer dizer, falou-se aqui a questão da ciência, não é, dos artigos científicos, das influências que estão muitas vezes por trás, e também existe, é assim, nós somos bombardeados todos os dias que *(...hã...)* 50% dos portugueses têm a doença *x*, no dia seguinte 30% dos portugueses têm a doença *x*, e basicamente chegamos à conclusão que todos nós temos 20 doenças e não sabemos *(...hã...)* Não é? Pronto, estamos todos dentro daquela percentagem, não é? E a questão é que muitas vezes, OK, será que é assim tão correcto falar dessa forma, *(...hã...)* será que isso é mesmo assim, ou que existe também uma questão de poder, porque informação, passar determinada informação para os meios de comunicação social também representa um determinado poder para uma determinada classe, não é? *(...hã...)* Será que não existe também esses mecanismos por trás? Será que os artigos, depois, não é o caso, não é, mas será que a ciência é feita, isso é uma questão que me faz pensar muitas vezes, mas será que a ciência é feita de facto *(...hã...)* de uma forma... [C.: Isenta.] isenta?

**L.**: Sem conflito de interesses.

**J.**: Eu também tenho essa opinião.

**B.**: Não é, não é, não é, mas isso pode-se comprovar.

**J.**: *(...hã...)* Mas, e essa é uma das questões, é a regulação da ciência, a regulação dos meios de comunicação social, eu acho que é absolutamente fundamental, porque aquilo que se vê hoje é, os critérios são assustadores, e lá está, é que depois se também está a lançar notícias para o público que se calhar as pessoas não vão notar porque já estão tão saturadas de, de títulos sensacionalistas que acabam por não, por não ligar. Se bem que, por outro lado, nós temos outro fenómeno que é, tivemos fenómenos como a história das vacas loucas, etc., se não fosse a pressão pública, se calhar ainda hoje continuávamos a comer carne de vaca contaminada, não é? Não sei, há aqui um meio termo que é muito complicado.

**H.**: Eu, pessoalmente, por definição, acho que todos os dados devem ser públicos para toda a gente consultar, independentemente do tipo, neste caso isto, mas em todos *(...hã...)* Mas é preciso também ter um pouco cuidado é que, pá, cada um de nós é especialista em uma área, o que significa que somos leigos em cem. *(...hã...)* Todas as notícias, e os jornalistas são leigos em quase tudo o que escrevem. *(...hã...)* Isto não é uma crítica, eles estudaram para uma coisa, muitos poucos estudaram bioquímica e a seguir jornalismo. Então, o que eles estão a falar, não sabem do que é que estão a falar, então, é preciso realmente na comunicação, *(...hã...)* e como disse, *(...hã...)* haver a possibilidade de conseguir chegar aos dados originais e não apenas ao resumo do resumo que o jornalista fez.

**B.**: O resumo é uma distorção. A melhor forma de ver isso é ir, por exemplo, ao PÚBLICO, à editora de ciência, N., ou lá como a senhora se chama, e vamos ver, *(...hã...)* sei lá, *x* por cento dos portugueses sofrem de não-sei-quê, de acordo com o estudo da *Nature*, e depois tem o *link* para o estudo da *Nature*, e a gente vai ver o estudo da *Nature*, e não é aquilo que está a ser dito, nem os cientistas fazem esse tipo de informações assim extremamente bombásticas, não é?

**M**: Deixem-me centrar aqui um pouco a conversa agora à volta do estudo. O estudo tem cinco anos, este estudo custa uma pipa de dinheiro, não vos digo porque ele é ofensivo *(...hã...)* e é um estudo só para criar *(...hã...)* regras comuns, não é? Regras comuns na maneira como colhemos as amostras, na maneira como analisamos as amostras, na maneira como divulgamos os resultados e também na maneira como *(...hã...)* divulgamos e usamos *(...hã...)* se vamos para o público, se vamos para os políticos, se vamos para a indústria, portanto, os resultados, como é que damos os resultados, etc. Num mundo perfeito, em três anos, mais ou menos, não é, o que é que vocês esperavam ver conseguido com um projecto destes, em que têm uma série de carolas aí a, a gastar tempo e dinheiro, muito dinheiro, para criarem estas regras comuns de funcionamento. O que é que seria expectável encontrar?

**I.**: *Guidelines*.

**M**: *Guidelines*?

**B.**: Protocolos. Sim, *guidelines*.

**E.**: Protocolos bem definidos da recolha de dados que fosse comum a todos os países [Manual de boas práticas] de boas práticas.

**M**: Isto tem interesse para o público em geral? *(...hã...)* Pensando agora em termos do cidadão, não é, *(...hã...)* que ecos é que vocês gostavam de ter no final do projecto, o que é que gostavam de saber do projecto? Daqui a três anos....

**B.**: O que é que se fez com, com as conclusões. Isso foi passado para os Governos e para os Ministérios da Saúde dos vários países e daí para os médicos, para os hospitais, para os centros de saúde?

**L.**: Exactamente.

**H.**: Ou até um bocadinho mais à frente, que é *(...hã...)* seguindo nessa linha de pensamento é *(...hã...)* que tipo de obrigações é que, conclusões científicas, *(...hã...)* se transmitem *(...hã...)* aos governantes, isto é, se realmente todos os estudos na Europa apontarem numa dada direcção, *(...hã...)* o que é, qual é a obrigação de quem nos governa de seguir *(...hã...)* as sugestões, não passam disso, de, de, das conclusões desses estudos.

(1:16:25)**K.**: Eu sinceramente acho que é poucochinho, acho que é gastar muito dinheiro, não é, é, é envolver muita gente, não é, para depois... é poucochinho, não sei, espremido bem depois não... não me parece.

**M**: Mas a ideia que o colega deu, que é interessante, que é *(...hã...)* usar estas conclusões para definir como é que os governos devem reagir também à informação criada, não é, *(...hã...)* isso também é pouco para vocês, não chega, onde é que iriam mais?

**J.**: Eu acho que as regras europeias são interessantes, e acho que têm funcionado nos últimos anos, ou seja, se se conseguir criar um exemplo, daquele género ali, uma série de produtos e que seja obrigatório fazer esse controlo diário, criar alternativas para que isto não aconteça, eu acho que isso será, será bastante importante. Obviamente, lá está, mais uma vez, não estamos sozinhos, a Europa não está sozinha, não é, e há *n* exemplos de *(...hã...)* químicos que eram suprimidos dos perfumes da Europa, e depois continuam a ser utilizados na China, etc., pronto, e isso também dificulta um bocadinho...

**M**: Aí estamos a falar de regras comerciais, é isso?

**J.**: Sim. Eu acho que sim. Acima de tudo porque acaba por ter aí depois.

**E.**: Mas, eu acho que passar a mensagem para os decisores políticos acho que é, se isso, se isso for feito com sucesso, eu acho que é uma das partes mais importantes porque, foi como o H. estava a dizer, os políticos são quê, são especialistas em economia, alguns não são especialistas em nada, portanto, alguém tem que lhes dizer que isto realmente é uma preocupação e se estão a gerir países, Europa, *whatever*, têm que saber que aquilo é uma preocupação, portanto, têm que lhes dar essa informação também da forma mais correcta possível para que lhes seja possível a eles tomar uma decisão informada. Porque se eles também não tiverem conhecimento do que é que se passa, eles também não vão saber. Eles não são bioquímicos, não são, não são biólogos, portanto, eles também não vão saber. Se isso for feito com sucesso, esse projecto por si já valeu, porque grão a grão enche a galinha o papo, não é? Acho que não é perdido. *(...hã...)* Claro que depois o que é que os políticos, o que é que os decisores políticos fazem com essas informações aí é a pergunta do milhão, não é?

**I.**: Espera. Posso intervir? Eu estou a fazer confusão ou então há aqui qualquer coisa que não me bate certo. Concordo plenamente com a E., mas eu pensei que os resultados que se procurariam era mais para regular futuros estudos, ou seja, para incentivar boas práticas e, e, ou para tornar tudo muito mais homogéneo em futuros estudos, e os resultados desses estudos é que se aplicaria àquilo que a E. diz.

**H.**: Sim, sim.

**I.**: Não sei se estou a fazer confusão.

**C.**: Esperemos que seja isso.

**E.**: Mas mesmos até...

**M**: Portanto, as regras, as regras, as regras vão até ao final, inclusivamente, as regras sobre a maneira como se divulga e como se pressiona entidades que têm responsabilidade, não é?

**E.**: E mesmo até para regular as próprias investigações, ou seja, nós para fazermos, nós para fazermos também investigação temos de pedir autorização às nossas autoridades competentes, não é? Convém estarmos todos na mesma linha para obtermos todos os resultados da forma mais correcta possível.

**C.**: Eu espero, eu espero que isto seja realmente [IMPERCEPTÍVEL] disseram, *(...hã...)* de se tomar cuidado com os resultados destes estudos, ou seja, o que é que estes poluentes podem realmente nos fazer mal. Espero, porque isto pode ter um lado perverso também, não sei se é esse ou senão, que é os políticos poderem saber que "eh pá, podemos continuar a dar carne a esta malta durante mais... *[riso de vários participantes]* ...não há problema nenhum". Isso tem um lado perverso. Os políticos de hoje, eu não confio nos políticos de hoje. Não é os de cá, é os de cá e os da Europa, não confio. Há um lado perverso no meio disto tudo. Isso agora, realmente, com esta...

**M**: [IMPERCEPTÍVEL]

**C.**: Exactamente, de repente alertou-me para que, queira Deus que não seja ao contrário que é os políticos querem saber, eh pá, se isto pode continuar assim, vamos embora, toca a andar, os poluentes todos, as fábricas, etc., que ainda não há perigo, pá, porque durante 50 anos isto aguenta-se tudo, não corremos perigo. Não sei se será isso, eu espero que não. Eu quero crer que isto seja um objectivo de nos proteger a nós cidadãos individuais.

(1:21:03)**CM**: Pegando ainda na confiança que começou por o C. há pouco, na ciência, e depois pegando ali na desconfiança do B. da ciência, hoje diz uma coisa, e amanhã diz outra...

**B.**: Não é na ciência, não é na ciência.

**CM**: Nos cientistas.

**B.**: Não, é numa certa imparcialidade ou uma certa, por exemplo, o fazer ciência com ciência. A ciência, ou o conhecimento científico, pelo conhecimento científico.

**CM**: Mas vamos esquecer os estudos em que há conflitos de interesses. Grande parte, a maior parte dos estudos não têm conflitos de interesses. Aqueles estudos que são realmente úteis. *(...hã...)* Eu achei interessante porque aqui a questão da confiança da ciência é, ao contrário da confiança dos decisores políticos, é uma confiança quase total, como se a ciência fosse uma coisa exacta e não se pudesse contradizer quando a ciência hoje descobre uma coisa, amanhã pode descobrir exactamente o contrário. *(...hã...)* Mas o que eu gostava de, de, de perguntar é: quanto aos decisores políticos, eu acho que este exemplo aqui da Suécia é interessante porque, quer dizer, foram os cientistas que de facto foram medir, mas depois a restrição no uso foi uma medida política, não é, *(...hã...)* como é que vocês fantasiam a forma como chega a informação científica até aos decisores políticos. Se é *(...hã...)* os decisores políticos que recebem por e-mail 500 estudos que foram feitos sobre um determinado químico, *(...hã...)* como é que acham que essa informação chega até aos decisores políticos. Ou será que há aqui alguns intermediários. Não nos podemos esquecer anualmente são feitos milhares de estudos *(...hã...)* em toda, em todo o mundo, *(...hã...)* uns contradizem os outros, sim, é verdade, *(...hã...)* mas como é que acham que esta informação chega até ao decisor político. É o decisor político que vai ler o artigo científico A, B, C, D, e depois tenta tirar uma conclusão, ou se há aqui algum intermediário que vocês estão aí a ver que possa fazer esta, esta, esta filtragem, *(...hã...)* que possa informar, informar o decisor político.

**C.**: Depende. Eu penso que depende. Eu se fosse político e quisesse saber, eu preciso que aquela fábrica continue a trabalhar porque a economia do país precisa dela. Eu fazia a pergunta ao cientista: "Eh pá, quanto tempo é que eu posso continuar a dar cabo disto tudo." Isto se for a perspectiva do político. A perspectiva do cientista quando faz a investigação é numa base científica, portanto, ele diz: "eh pá, olhe, isto pode durar mais não sei quanto tempo ou isto faz mal a isto, a isto, a isto." Para o político, eu se eu fosse político, não me interessava o que é que aquilo faz mal, ou ia ler que aquilo é assim, assado, não, não, eh pá, quanto tempo é que eu posso manter a fábrica aberta.

**M**: Mais perspectivas.

**C.**: Penso que era assim. Se fosse político.

**D.**: É assim, se isto é um projecto da União Europeia, penso que este projecto, este trabalho irá directo para a União Europeia e lá haverá pessoas dentro da especialidade que irão analisar.

**CM**: Mas pensam em pessoas? Mas pensam em pessoas?

**D.**: E para mim, e para mim o correcto seria que depois de analisar o documento, e que desse origem a directrizes para os vários países, saíssem directrizes da parte da União Europeia para os diversos países, quer ser da própria União e de outros países que também estejam no estudo.

**M**: Portanto, há uma subida e depois há uma recomendação que é polvilhada pelos vários países. F..

(1:24:48) **F.**: *(...hã...)* Tenho aqui na pergunta e na ideia de os políticos receberem 500 e-mails de artigos, faz sentido pensarmos sobre isto. Isto é super-interessante porque os artigos não têm todos o mesmo peso e, portanto, tem que haver alguém que seja especialista em ler artigos, alguém da área que seja especialista em ler artigos, pelo menos a mim faz-me sentido que consiga fazer revisões, e depois das revisões fazer *guidelines*, e depois *guidelines* então comunicar aos órgãos políticos o que é que deve ser feito. Eu acho que não pode ser, não podemos dar, não se pode fazer uma chuva de informação como é normal uma pessoa que não consegue gerir esta informação porque nós também não conseguimos gerir outras, tem que ser, tem que ser resumido, e tem que ser focado, e tem que ser alguém especialista em fazer resumos que seja da área.

**J.**: E neste caso deverá haver comissões no Ministério da Saúde, suponho eu, que estejam dentro destes, destas, digo eu.

**C.**: Mas depois passa tudo pela vontade política. A gente vê o caso do amianto aqui em Portugal, para não falar de outros, que toda a gente sabe que aquilo faz mal, continua a haver escolas de miúdos que têm os telhados em amianto e não mudam os telhados. Portanto, não é por falta de vontade dos políticos, é porque há outras prioridades.

**D.**: Fiscalização. Aposta na fiscalização.

**M**: I., I..

**I.**: Sim. *(...hã...)* Além de tudo o que disseram, é lógico que funcione assim, mas eu penso que a informação adquire pesos diferentes conforme os grupos de pressão. *(...hã...)* Os políticos, hoje em dia, funcionam... hoje em dia, (...) desde o início do século XX que, nos Estados Unidos até começou antes, os *lobbies*, são grupos de pressão que fazem uma informação que é menos relevante do que outras chegar mais depressa à decisão. O nosso sistema político funciona da seguinte forma: nós podemos recolher não sei quantas mil assinaturas para que o assunto seja levado à Assembleia da República. É verdade, é correcto, e ainda esta semana ouvimos uma notícia sobre isso. O problema é que, por vezes, tem que esperar um bocadinho, esse bocadinho, desculpa, esse bocadinho podem ser vinte anos - estou a exagerar - porque entretanto houve outras formas de se fazer chegar lá acima assuntos menos relevantes do que aquele que realmente consta das assinaturas das pessoas, da petição que as pessoas fizeram, e portanto não é tanto a questão de, de, de os intermediários, e da forma da organização, se vem da União Europeia para baixo, ou de baixo para cima, mas é sobretudo a perversão do sistema.

**M**: Portanto, nós começamos a falar de biomonitorização, estamos a falar das não-virtudes humanas [risos de outros] *(...hã...)* mas deixem-me focar e pensem agora em termos de futurologia, mas futurologia a curto prazo, que é de 3 anos, não é, é mais ou menos o que nos falta até acabar. *(...hã...)* Pensando na boa vontade e pensando que há mecanismos até de garantir que os políticos fazem tudo o que deve ser feito, o que é que seria expectável que acontecesse do ponto de vista da biomonitorização humana, que políticas é que deveriam de ser feitas, isto no fundo é o *mot de pass* para a última pergunta, nós já estamos aqui há uma horita e vocês daqui a bocadinho começam a rogar-nos pragas *[riso de vários participantes]* mas *(...hã...)* se pensarmos em termos de daqui a três anos, que políticas é que devem ser construídas para que a biomonitorização humana seja melhor, seja bem feita. Agora, pensando em termos políticos, não é? Porque em última instância a investigação precisa de dinheiro *(...hã...)* e tem que haver uma parte política para que haja investigação e biomonitorização mais adequada. Que políticas seriam essas para melhorar a biomonitorização do ponto de vista da sociedade humana.

**E.**: Primeiro, estabelecer um plano ao nível do nosso sistema de saúde que permitisse às pessoas serem monitorizadas...

**M**: Do nosso, português.

**E.**: Nosso, a nível Europeu, se este estudo é europeu... Nosso, a nível Europeu. Não sei muito bem como é que funcionam os planos de saúde das outras pessoas a nível Europeu, mas pelo menos o nosso sei que funciona, que tem as suas mãos livres, vá.

**M**: Um plano, um plano de biomonitorização. Vamos estudar primeiro aquela substância, vamos estudar aquela, vamos estudar aquela.

**E.**: Definir um plano que, que permitisse, ou seja, que permitisse que o nosso sistema de saúde fosse capaz de suportar a biomonitorização e que isso implica o quê, recolha de sangue, urina, um certo tipo de amostras que, se nós formos ao nosso sistema de saúde, se calhar se formos todos agora, quero fazer uma carrada de análises, eles dizem não pertence a um grupo de risco e, portanto, não tem nada que estar a gastar recursos ao nosso sistema.

**M**: OK.

**J.**: Há algum observatório?

**M**: Algum?

**J.**: Observatório nesta área, tal como existem numa série de outras áreas.

**M**: Está a ser pensado uma coisa, não sei se podemos chamar aquilo um observatório, mas um *National Hub* nesta área.

**E.**: Uma coisa desse género seria...

**B.**: Tal como nós estamos aqui nesta, nesta sessão, neste *focus group* com as pessoas, dizendo o que é que as assustavam, que é que as preocupavam, se calhar é um pouco por aí. Porque se eu quiser estudar uma quantidade, se eu quiser biomonitorizar uma quantidade coisas que as pessoas usam, naturalmente, não, o objecto de estudo, o vosso objecto de estudo enquanto cientistas não pode, não pode basear-se na preocupação das pessoas. Mas é mais fácil, é isto que eu quero dizer, é mais fácil *(...hã...)* convencer as pessoas, digamos, a biomonitorizarem-se se for uma coisa que elas percebem que é directamente do seu interesse, e que nos esconde, de certa forma, muitas das suas ansiedades e aspirações.

**M**: Hum, hum. OK. Portanto, políticas que, no fundo, sensibilizem as pessoas para a influência da biomonitorização humana na saúde.

**I.**: Que as pessoas podem pressionar o poder político. Na ética e deontologia médicas, muitas vezes têm de se tomar decisões contraditórias, e essas decisões contraditórias muitas vezes têm [IMPERCEPTÍVEL] e é para contrariar, por exemplo, então, a senhora já tem 90 anos e vamos-lhe fazer um implante, quantos anos mais ela vai durar e quanto custa o implante? É para contrariar um bocadinho esta, esta, este funcionamento que esta iniciativa também pode ser importante. Primeiro, chegar ao público e o público pressionar os políticos. Em segundo lugar, dar aos políticos ferramentas para puderem trabalhar.

**M.**: Mais ideias.

**H.**: Eu, por exemplo, ainda respondendo à questão. *(...hã...)* Nós sabemos que, regra geral, os políticos dão atenção a quem faz mais barulho. *(...hã...)* E, muitas vezes, é assim. *(...hã...)* Acho que, mesmo em assuntos tão específicos, técnicos, como este, convém também haver essencialmente, haver uma, um crivo antes de, antes de chegar ao político, exactamente para não receber 500 estudos diferentes. Aí, na minha opinião, as Universidades é quem tem dever e a possibilidade, pela sua proximidade com, com a tutela, de, de fazer essa, essa análise do que é que é relevante. Relativamente a, a como, no fundo, o que fazer com, com esta ideia. *(...hã...)*Acho que é importante, na minha opinião, conseguir perceber o que é que, neste ano, por exemplo, onde é que houve mais problemas de saúde, por exemplo, *(...hã...)* onde é que houve um maior aumento relativamente, por exemplo, a anos anteriores, e então o que é que diferenciou este ano do ano anterior e aí sim conseguir perceber o que é que são os possíveis problemas do futuro, porque há muita coisa que nós já sabemos que causam problemas, como é o caso do amianto, *(...hã...)* mas há muita coisa que nós não sabemos o que é que causa. Os microplásticos, por exemplo, ninguém sabe exactamente... sabemos que existe no ambiente, mas o que é que nos afecta a nós, ainda não sabemos, e é um bocadinho projectar essa ideia no futuro para, para conseguir depois fazer alguma coisa a tempo quando o problema realmente atingir a maioria da, da população.

**M**: OK. *(...hã...)* Ias dizer alguma coisa? Deixem-me, deixem-me, antes de concluirmos, só lançar aqui uma pequena provocação. Imaginem este cenário: estamos a gastar muito dinheiro, neste estudo, OK? Daqui a três anos arranjávamos maneira de que, em termos de política, *(...hã...)* política de saúde pública, ou de biomonitorização pública, fazermos uma coisa do tipo, todas as crianças, todos os anos, têm que, para continuar a estudar, a escola obriga a que lhes tirem sangue, que recolha de urina, porque a biomonitorização é fundamental. E já agora, sempre que eu faço uma, uma, um exame num laboratório qualquer, *(...hã...)* uma parte do meu exame, da minha urina, do meu sangue, vai para um banco de biomonitorização. Isto é um filme, quando pensamos em políticas públicas de, nestas áreas, um filme possível. *(...hã...)* Outros, políticos de facto de informação, o Estado ter uma atitude muito de sensibilização, alguém disse isso, já não me recordo, foste tu, não sei, ou o C., *(...hã...)* sensibilização ao público, isto é tudo importante, portanto, se vocês foram contactados para fazer, para participarem num estudo, aceitem. Isto é mesmo muito importante para compreendermos melhor *(...hã...)* o efeito dos químicos no corpo humano. Isto são medidas, não é? Isto é bom, é mau, como é que vocês pensam em relação a isto?

**H.**: É muito problemática a questão da privacidade e dos dados recolhidos *(...hã...)* e nós já ouvimos falar noutros sítios que a utilização de dados médicos para calcular o valor do seguro de saúde que vai pagar e a partir do momento que nós *(...hã...)* deixamos os nossos dados se utilizados, nunca sabemos exactamente para o que é que vão ser utilizados. Podemos pensar que vai ser apenas para isto, mas no fundo, *(...hã...)* perdemos logo o controlo, quase aquela coisa de colocar uma fotografia, fazer um *post* com uma fotografia no Facebook, não sabemos onde é que aquilo vai dar. *(...hã...)* Seria necessário haver uma grande confiança *(...hã...)* nessa recolha que os dados realmente nunca iriam ser ligados a um indivíduo, isto é, se eu fosse, se um filho meu, eu ou um filho meu *(...hã...)* fosse sujeito a uma colheita, que essa colheita nunca iria ser conseguida ligar a uma pessoa em específico.

**M**: OK. Preocupações em relação aos aspectos éticos de uma medida deste tipo.

**C.**: Penso que, numa situação dessas, era já num estágio de desespero, de toda a população tem que ser biomonitorizada, ou seja, a gente já estava de tal maneira contaminados que tínhamos que fazer qualquer coisa, os tais alertas, de travar *(...hã...)* e isso seria, era, era, mal.

**M**: Era essa a mensagem que soava, não é?

**C.**: Eu acho que, pelo menos para mim soava.

**M**: Então, eu exagerei, não é, criei um cenário de facto preocupante, então, mas só para vos situar sobre o que é que estamos aqui a falar de políticas, o que é que era interessante daqui a três anos então? Se calhar, não iríamos tão longe, não íamos obrigar as pessoas todas a fazer biomonitorização, mas o que é que seria uma política interessante daqui a três anos, com base nisto tudo, conseguem imaginar alguma alternativa mais razoável?

**J.**: As questões de fundo são complicadas, por acaso. *(...hã...)* Esta questão da obrigatoriedade *versus* a liberdade de o fazer é complicado.

**C.**: Não é pela obrigatoriedade, é todos, todos. E quando é todos é porque a coisa está mal.

**J.**: Eu consigo perceber as duas situações. É assim *(...hã...)* nós...

**C.**: Porque a liberdade em excesso também não é bom. As vacinas, por exemplo.

**J.**: As vacinas.

**C.**: Eu quando [IMPERCEPTÍVEL] levava o boletim de vacinas em dia porque senão não me podia inscrever.

**J.**: Exactamente.

**C.**: Mas porque é que era isso? Era porque a gente tinha de estar vacinados, tínhamos de estar protegidos, e as vacinas não nos faziam mal. Depois agora surgiram as teorias de que a vacina faz mal.

**B.**: Isso não é uma teoria. É um absurdo. *[riso de vários participantes]* Mas é verdade.

**M**: [IMPERCEPTÍVEL]

**C.**: Mas isso é uma coisa diferente de um estudo, de uma biomonitorização.

**J.**: É, é, é. Mas as vacinas o que aconteceu é, é não havendo obrigatoriedade, existem grupos que não estão a ser vacinados e constituem risco para toda a gente, não é? *(...hã...)* Portanto, esta fronteira é muito, muito complicado.

**C.**: Mas a biomonitorização é uma coisa diferente. A gente está a ver se você está doente ou não. Não é você está doente. Portanto, eh pá, venha aqui que a gente vai estudá-lo. Não. Toda a gente está doente, à partida.

**M**: Isso é interessante. Portanto, para o C. a biomonitorização passa muito para detectar se estamos doentes, não é? *(...hã...)* É um pouco essa a lógica.

**C.**: Eu passo. Não. É para a gente estar alerta para os perigos de, das substâncias poluentes.

**I.**: Eu faço uma pergunta: é um problema de saúde pública ou não é um problema de saúde pública. Porque dependendo da resposta a esta pergunta, pode-se ir mais à frente em relação às liberdades individuais ou não. Um médico de saúde pública pode mandar suspender, se for caso disso, um, um concerto.

**H.**: Uma, uma possível escapatória, um caminho quase para essa questão da obrigatoriedade de todos é, nós já temos um, um banco de dados, por assim dizer, bastante  grande em termos de análise de, de sangue, por exemplo, que são efectivamente as doações que conseguimos ter uma panóplia de idades, géneros, profissões, quase muita gente mesmo doou sangue, se calhar, uma alternativa menos problemática seria, simplesmente, que parte dessa doação fosse *(...hã...)* guiada para o estudo, para o estudo, até porque já se garantia aí, à partida, *(...hã...)* o anonimato. *(...hã...)* São normalmente as doações são feitas em grandes grupos, não é?

**I.**: Mas aí também se perdia o controlo, se queremos fazer um estudo específico relacionado com determinado assunto localizado, o universo a ser estudado não pode ser desta forma, tem que ser pessoas que vivam naquela zona, por exemplo.

**M**: OK. Mais ideias de políticas de saúde, ou políticas de biomonitorização daqui a três anos que seriam interessantes, mas...

**J.**: Isto acaba por ser uma espécie de um rastreio, não é? *(...hã...)* E os rastreios funcionam mais ou menos em Portugal *(...hã...)* Portanto, se calhar, se se usasse os mesmos mecanismos, se calhar conseguia-se fazer. Normalmente os rastreios, quando vêm para a área da saúde, as pessoas acabam, de certa maneira, por, por aceder. *(...hã...)* Não sei se, apesar dos rastreios serem muito fragmentados, e etc., mais vão funcionando.

**I.**: Sá há duas formas possíveis: ou através de uma boa mensagem que leve muita gente voluntariamente a aderir ou através da imposição. A imposição, como eu dizia há pouco, só se for muito bem justificada. Uma boa mensagem às vezes faz mais do que a imposição, porque tal como se viu na vacinação, embora a vacinação não seja obrigatório hoje em dia e não tenha aquelas consequências que o C. falou, não é, há muita gente que se escapa da vacinação porque não está bem informada ou porque está mal informada, porque também há a teoria da conspiração que diz que "ah, o senhor vai apanhar uma vacina contra a gripe, mas lá puseram-lhe isto e puseram-lhe aquilo"...

**M**: Mais?

**C.**: Eu penso, eu penso que isto vai ser uma boa base de trabalho para quando a coisa estiver complicada. *[riso de vários participantes]* Portanto, não está, não está, tanto que não está que isto não é uma imposição, está-se a estudar o que é que se há-de fazer, porque se já estivéssemos realmente todos condenados, se calhar, a coisa era muito mais rápida. É como nas guerras.

**D.**: Eu apostaria mais no rastreio. Porque é uma forma, digamos que entre-aspas, mais suave, porque as pessoas têm aquele conceito do que é que é um rastreio *(...hã...)* e que não choca.

(1:42:15)**L.**: Uma boa informação.

**M**: Portanto, aí apostar mais em termos de políticas de informação.

**L.**: Sim, como se estava a dizer, às vezes é preferível e é mais fácil do que a obrigação, porque a obrigação a pessoa tende, há sempre as pessoas que atacam, como no problema das vacinas. Eu acho que uma boa informação é mais fácil.

**D.**: Com sensibilização.

**M**: Sensibilização.

**D.**: Com sensibilização.

**C.**: Mas penso que este estudo é muito importante, muito importante.

**M**: OK, acho que é uma boa maneira de terminarmos. *[riso de vários participantes]* Muito obrigado pela vossa disponibilidade e por participarem connosco nisto. Nós vamos *(...hã...)* transcrever estes dados *(...hã...)* que vão ser analisados, há-de haver aqui um ou dois escravos *(...hã...)* a transcrever isto e depois a partir um pouco a cabeça a ver as grandes conclusões desta conversa. *(...hã...)* Nós tentamos sempre, embora às vezes demore bastante, mais do que queríamos, mas gostávamos depois de enviar-vos *(...hã...)* resultados para vocês lerem, pelo menos um pequeno resumo, e depois ao lerem, se quiserem acrescentar mais alguma coisa, *(...hã...)* era, era óptimo. *(...hã...)*  Às vezes é difícil para nós fazer isto em tempo que seja útil e que, e que, enfim, e que as pessoas ainda se lembrem do que aconteceu aqui, mas vamos tentar fazê-lo. Muito obrigado e até uma próxima.

(1:43:42) [FIM]

**Transcript of the Irish focus group (September 2019)**

Transcript of HBM4U focus group 26th Sept 2019 , As amended 08/10/2019

Dictator 1 (Male): Alright, so, yeah, that is working, then, I have an app which a colleague of mine gave me, which does / tells the same thing. So, if you want to practice being Frank Sinatra or Amy Winehouse, now is the time to do it. So, with the number of questions to get through us, so I am trying to get to stagger the ideas, we get a good conversation going on in this topic.

Q1 So, the first question is do you have any concerns about being exposed to pollution in Ireland? So maybe we could just indicate or just kick off and we will go around the table if there were any particular concerns, so.

Dictator 2 (Female): Yes, I do, in general. Cycling here today on a relatively free road, I am still conscious of breathing diesel fumes, no more than what Joanna said also you know I used shampoo this morning, and I have a toothpaste which I believe is better than the fluoride ones, but how do I know is there something still in that toothpaste that is not good, and I tried to have as little as possible in terms of makeup, and you know, I am using really good pure baby-type creams and things like that, but what about all the stuff I do not know about.

Dictator 1 (Male): What about anybody else, is anybody else?

Dictator 3 (Male): I suppose I take her point about diesel fumes, and I am probably most concerned about either atmospheric or if it is a water pollution in our drinking water. Living where we do in Stillorgan, I am probably not that concerned about the atmospheric pollution out there, but if I lived in a big house in Sandymount, I would want to make sure that the effluent from the incinerator was being very effectively scrubbed, man, I do not know, I can see is we often walk Sandymount Strand with our dog and there is a non-ending plume of what is supposed to be steam coming from it but what’s in steam but I presume that EPA are monitoring that.

Dictator 2 (Female): As well as that there Cobh in Cork has an incinerator quite close by and it has one of the highest cancer rates in Ireland. t is, known, like it has been published and it is quite an obvious area for cancer in Cork due to probably pollution in that area.

Dictator 3 (Male): But again then yeah, you know, pharmaceutical plants in little island.

Dictator 2 (Female): Yeah. They are all there. Yeah, they are all kind of based in that area. It does not come from nowhere.

Dictator 1 (Male): I presume it is not true, but there was a story going around about 10 years about when Pfizer started making Viagra in Ringaskiddy, the birth rate increased by a couple of about 100%.

Dictator 4 (Male): I am quite concerned about a good few things, mainly water pollution, drinking water. My friend said to me last week that we are drinking a credit card piece of plastic in our water every week, and I did not locate that myself now but sunblock, I am concerned about the chemicals in sunblock and cosmetics and that kind of stuff and that is just scratching the surface.

Dictator 2 (Female): I think there is so much to so many things but if it was not for food I suppose, just recently, I had cut an apple from a tree, from a tree in my father’s garden and cut it in half, and within a minute, it starts to go brown, but if you cut an apple from the shop in half, it stays perfect, so what is making that stay perfect, there something, you know, whereas when they just pick it off the tree with no pesticides or anything, it would start, the minute you cut it, it would start to go brown, but when you buy them, they don’t, they stay like that.

Dictator 3 (Male): That is probably because fertilizer going around the tree.

Dictator 2 (Female): The apple tree, I do not know, I think that maybe I think.

Dictator 1 (Male): The browning is dependant on the sugar content it can cause a Maillard reaction. It occurs is of course in all the fruits and vegetable, and the more sugar you have in, the quicker it will go brown.

Q 2 Dictator 1 (Male): So, I might move on because of what you have mentioned around a range of kind of those vectors and sources, you know, we call them, what do you think the main barriers are to solving problem of pollution in Ireland, that is why I moved you on because I think this is quite a big topic.

Dictator 4 (Male): For me, it is getting young kids in primary schools, giving them a certain, how should I call this, a lesson to say what is right and what is wrong.

Dictator 1 (Male): Okay.

Dictator 5 (Male): But, it is not really a consumer, I mean, like I do not know the exact figures, but a huge, huge proportion of the pollution in Ireland is big corporations, it is not consumers. It is like, oh turn off your light when you are out of the room, and you know, do not use plastics straws anymore, but at the end of the day, like, it is as a consumer the blame is not really on us as much, not to say that we should not things, I think we should but this is also on big corporations to stop it.

Dictator 1 (Male): But, what are the barriers to dealing with pollution?

Dictator 5 (Male): I think that what I mean.

Dictator 2 (Female): It is surprising that we do not hear our government, where is the media involvement in any of this. It should be on our screens. We should be, we have been told about buy this nugget and McDonald’s this and that, why are we not getting the balance, there is none. There is not the information out there, why?

Dictator 6 (Female): And in the same way as the Irish Government protected Apple for the reason of creating jobs, there are probably other chemical industries that are being protected because they bring in money.

Dictator 2 (Female): I can it is the economic-politics diad where money drives politics and politics drives big business, they all have their own agenda hidden in the middle of it, and they want to bring in money. They will close their eyes to a lot of things, they will close down their doors to opening of avenues that they should open up to create healthy wholesome environment for others just to get that money into the country. Money drives so much that is very, very sad at the expense of its own citizens, at least in Ireland, and the other thing is I think that we do give a good amount of credence to pollution as a small country, but we have very, very insignificant compared to some of the other big countries internationally. So, I think we do quite a bit clad wise for our size, relative to other countries internationally, but I do not think we do enough within our own country from the political standpoint.

Q 3 Dictator 1 (Male): Are there anything in terms of pollution and health and environment that you would like to know more about.

Dictator 6 (Female): Figures, facts, honest education, honest facts, presented for the public.

Dictator 5 (Male): I think it has been plain English, because a lot of the information that comes out are, so even if you think food packaging, very few people understand the contents of and even that I believe if you would take education and you put it to the education system, you get the next generation on the level of awareness. If you want to educate ourselves and others, you need to be targeting that talking about it. I understand in my bracket, some one else understands in their bracket, so one size does not fit all, the wording, the terminology that the use needs to be related I think to the individual _______ 08:17 so you can get an acceptance and understanding.

Q 4 Dictator 1 (Male): Okay, the next thing now is to move on, and let’s talk a little bit more about biomonitoring the field that Joanna kind of introduced us to earlier, so, has anyone ever heard biomonitoring before, you heard about it today.

Dictator 2 (Female): There is a girl from the Guardian newspaper there, a journalist, they monitored her, they put a …..I am not familiar with what the word was, but they put something on her wrist, a bracelet, yes, and they monitored her for I think like 48 hours, everything that she did. They took her back and did a range of tests on her, and I there were, of all these chemicals, it is what we were saying earlier on that some are good and some are not, and a of them she was fine, and there were about four that were outstanding and that were raised in her body, that she got, and pesticides was one of them, and mercury was another. What she was saying was that she had had her teeth done years ago when they were using amalgam and stuff and this all came through, but she was saying what you will say to take those out was with worse to touch them, you know just leave it, I thought was very, very interesting.

Dictator 1 (Male): Is anybody else who has heard about this field?

Dictator 4 (Male): The Olympics, all right are.

Dictator 1 (Male): Yeah the drug testing aspect of it.

Dictator 3 (Male): Having in the hospital as I have all of my career. It is what I did for living under a different name.

Dictator 2 (Female): Yes, the terminology, I was not familiar with, but I know a what it is.

Dictator 1 (Male): I think that is it. It could be screening, it could be medical and clinical testing. It could be giving urine and hair samples,

Dictator (Female) Exactly

Dictator 1 (Male): ….which what they use, they use hair, teeth you knows, sometimes foetal blood, that can all be used as what they call matrices _______ (10:20). No one is taking part in this study themselves, not in this frame…you maybe that you did a blood test with your Doctor.

Q 5 So now, is this really about making changes as a result of the project, okay, what I want you to do is to imagine that you have been beamed into the future. Let’s assume it is 2023, the project is over, and we are all a few years older, no one wants to be a few years older, but let’s pretend. What things should we have found out about Ireland between now and then, what things should we have found out, what things would you like us to find out about in the next four years over human biomonitoring product for Ireland. What would we like to find out, what would you like us to find out?

Dictator 5 (Male): I would like you to find out that there is a lot of information already there about the effects of certain chemicals, especially in the food industry that are very harmful to health and to back up them studies and to expose them.

Dictator 1 (Male): So you are interested in the food sector and food products. Is anybody else interested in any other products or any other products or any other sectors.

Dictator 6 (Female): The things you cannot see, you know, food, you can to some extent control, you can read the ingredients, and if you do not understand you do not buy basically that is my philosophy about it, but it is the things you cannot see or feel, what you are ingesting in other ways, so breathing and drinking.

Dictator 1 (Male): So anything inhaled or ingested?

Dictator 2 (Female): I think maybe consumerism advertising would be adjusted so that it is not targeted to the young population that are so consumer driven without any understanding or insight as to what is going on that maybe that the advertising has to have some component of awareness like the cigarette alert or the cigarette notification that you just do not go buy all this makeup by the ton and apply it, there is no consequence on you. You eat this or you follow that hypothetically or speaking about the vaping situation, the one that is in the United States at the moment, there was no consequence. People were aware it, they were promoting and promoting, and when the FDA were aware of it, but they were not doing anything either to minimise or advertise it as a harmful product. I think if there is product being advertised to younger people that there should be an alert associated with based on the facts that they find out through the biomonitoring.

Dictator 1 (Male): I think facts is the word probably. So, I suppose is what we are saying is in four years, what facts would we need, what main facts would you demand really? So, are there any particular products or mention to kind of food or things you inhale, are there any other areas or particular products? Sunscreen is mentioned in one of the earlier questions?

Dictator 2 (Female): Pesticides.

Dictator 1 (Male): And you mentioned consumer products.

Dictator 6 (Female): Pesticides. Yeah.

Dictator 2 (Female): And antibiotics in animals.

Dictator 1 (Male): In terms of?

Dictator 6 (Female): Coming through the food chain where it is not actually stated. These animals have been injected with XYZ antibiotics and we do not know.

Dictator 1 (Male): And because of, what aspect would you worried about there?

Dictator 6 (Female): Because it is not natural. It is, you know, even organic meat can have certain antibiotics administered, and it is not stated along with the process.

Dictator 3 (Male): The thing to worry about there is the increase, dramatic increase in bacterial resistance which is down to widespread veterinary use of antibiotics. Another thing I would be concerned about was of the female persuasion would be what I am painting on myself, I mean how toxic is the make up in my wife’s dressing table, it is like a chemical store room.

Dictator 4 (Male): Can I just add, see lakes and rivers and the effects of being in them, and say like the adverse of the effect of being in them, I did a lot of water sports so.

Dictator 1 (Male): So, it is recreational water and salt water, and rivers are all affected. Q 6 So, what type of impact do you think the project would have had four years, a project like this what impact do you think it would have had?

Dictator 3 (Male): I do not know what impact I would like it to have but being the cynic I am, I would actually be doubtful have you got the clout to influence government.

Dictator 1 (Male): Goes back to the challenges and the barriers earlier, does it not, the influences and the questions you asked Joanna at the beginning.

Dictator 3 (Male): I mean the whole thing is...

Dictator 1 (Male): And the awareness raising words and that came off earlier.

Dictator 2 (Female): I think it should be, it should create much more awareness for everybody that everyone is aware of this, the terminology as well, that’s more you know....

Q 7 Dictator 1 (Male): Do you feel empowered by information that you currently get, do you feel you get enough information about the environment and do you feel empowered by it?

Dictator (Male) No

Dictator (Female) You have to ferret it out yourself

Dictator 2 (Female): It is there. I think it is all there, that is not very easily accessible, so I think if you do look, you know, but it is not easily accessible.

Dictator 6 (Female): If you have given the importance of There may be an abundance of information that maybe have targeted in eduction level in that book. Secondly, it is in miniscule print that God bless us, and older people cannot even read without the magnifying glass. So, it is not really, it may be targeted in an ideal sense, but in practical sense, it is not targeted at all.

Dictator (Female): Make is user-friendly, you know, so that everybody will understand it

Dictator 1 (Male): Well, like what Michael I said earlier on, we need more information, but it needs to be in the format that everybody can understand. And I mean I have seen some of the environmental stuff, the ones the chemical safety data sheet came out, and to me, with a master’s degree in biochemistry an awful lot of those were totally unintelligible, even the one for if you are exposed waters, if you’re exposed to water, contact the doctor, seek medical attention.

Female: Yeah, yeah

Dictator 3 1 (Male / Maurice): And that’s the key aspect of the project turning scientific knowledge into understanding, how are we going to say, and reverse really the concerns are raised and the chemicals are targeted based upon public concern as much as maybe knowing the risk, that is part of the project.

Dictator 1 (Male / not Maurice): And again, if I am being very cynical selective to get it through to the politicians politics and we you are going to have to have it in language a 5-year-old can understand.

Dictator 3 1 (Male/ Maurice): So, communications and messages, it seems, of both ends, hearing from the beginning, what the risks are, knowing more, like, we are all uninformed about something. They are to get information readily accessible, is not it, and then the project really is trying to point each one into new directions, going forward, and new chemicals and new emergent chemicals.

Dictator 6 (Female): and maybe opening up avenues so people can get earlier and alerts or earlier concerns can be addressed, that maybe because of this there will be new avenues to get those in place sooner rather than later, like if there are concerns, instead of waiting six years for that to actually come to that, there are new avenues that things can be addressed earlier or at least looked into earlier.

Dictator 3 (Male): I think the carrot and the stick method is you get good information out in the right format to particular target audiences will be faster.

Q 8 Dictator 1 (Male): You are moving a bit into the next question range, and I want this to be free flowing, so I am letting it run and I am adding a few questions in it as we go along. So, we are still four years ahead and we all look exactly like this still, okay, how are the population made aware of the results, so yeah, how do you want to be made aware of the results four years down the track when you look at this raft these lots of chemicals.

Dictator 3 ? (Male): As we you said earlier, your honour, all of those it all boils back down to the education.

Dictator 2 (Female): But I would want it coming from those who are working in the field, who have the science, who have the facts, facts, facts. You can hear something on television like Oh, this is good, this is bad. I want somebody to say this is wrong for you because and scientifically, you know, people need that certainty because, what, this thing of fake news again. We do not know. It has to be coming from people who have a under standing and the an authority to give that information out, and then people may look to it.

Dictator (Male) …and independence

Dictator (Female): Yes, not coming from the pharmaceuticals or whoever it’s coming from

Dictator 6 (Female): And that there will be a watchdog who actually has teeth.

Dictator 2 (Female): And something watching the watchdog.

Dictator 3 (Male): I think that the other thing too is that we will would probably find out depending on our line of work, it will either be driven through, say the as a health & safety authority target and that’s how we would find out about it. We don’t normally find out from the Media.

Dictator 2 (Female): That child, Greta, standing up there in the United Nations, saying to the government, who know all of this, she said you know, and she just says look to the science. That was her big thing that she can stand there and talk, and that was very powerful as a child, being the adult, and the adults were sitting there like children. It is a The whole reversal of it, but at the end she said, look to the science, and that just sticks with me that if you can get the truth out, people are crying out for it.

Q 9 Dictator 1 (Male): I want to come up and question you the thing you raised. How do you want to be told about the risk around the sub chemical because it seems that when you look at any newspaper or the TV, the risks are everywhere. My wife says what is safe, you know. How would you like to be told about the relative risks of each of those different chemicals, for instance, that we are going look at. How would you want to communicate to you so you can actually make sense of what it means in terms of you and how you function, and the choice that you make in life?

Dictator 3 (Male): It may be How about something like one a month, you know, and this month it’s its chemicals in water, next one is chemicals in meat.

Dictator 1 (Male): And who should do that, how should that happen.

Dictator 3 (Male): Independently, but you know, I mean I know that health-wise, I mean I’m in to meant to have base diet this stuff, a lot doctors are independent, they would not go near big food companies, they won’t go near governments, you know what I mean, so that is how I judge it to be good information. So, if we have governments looking to independent bodies like an independent water authority, you know, that is monitoring waters, water pollution levels.

Dictator 1 (Male): So independent science seems to be....

Dictator 6 (Female): A I question it though because it is all, again it goes back to money. These people have to be funded and there has to be funding available. Where is the funding come from, even the independent bodies are funded by someone. And in order to get the information out, the money has to come from somewhere, and it is usually either funded by government or farmer or something else, so there is always a question as to how it gets out there or when it gets out there, and secondly, four years on, I mean, paper may be absolute in four years’ time. Environmentally it’s probably a good, but yet you have got technologies. They are going to be some of these. You look at a product and something zaps up on your phone to tell you that you know this is.

Dictator 3 (Male): Sorry for butting in but the money that the social media companies are making, it is fantastic. You open up an app on your phone and you get free advertisement, should it not be going through there? That’s it, that one of your ??? The other thing is if the state buys in to it and I am talking about many companies you will only get a message to out to put on someone’s pay slip, you put it on where they login, there is a number of things that we can do as a body to get that awareness away that is out there and what people do with the information is down to the each individual. If we’re going to get as message across, the only way to do it is through repetition. I do some of the training with our people and it’s through Maybe with something to try _______ (22:57) repetition, same message coming in on a regular basis, ok it may eventually become subliminal and you don’t read it but the point is, it’s there.

Dictator 5 (Male): I And do think there is a point to be made from not, for like actually making it in some way interesting like I mean, you can talk about like oh independent scientist presentings, newsreader are like scientists do not know how to present these things in such away that people will actually care, like, no one cares about climate change until Al Gore I will go or made a film about it. I mean, that was very alarmist, I mean you need some sort of something interesting, something alarming, something interesting. Yes Or else , I mean, no one cares otherwise because it is just another you know boring governmental scientific thing, yeah.

Dictator 3 (Male): I think people hear the truth though. Whenever you look, you can see something on the news or see a headline in a paper that says, you know chemicals in meat, lead or meats a carcinogen or you know. I think you pick up on if they got that something that is ral and true and relevant, you know what I mean, do if I think there is good information coming out, and someone is saying this is bad for you I think . . . .

Q 10 Dictator 1 (Male): And the results to come out, you have spoken a lot about you know consumers, I suppose, members of the public, and we are all members of the public, but are there other groups you think that particularly need to be targeted with the information and facts....

Dictator 2 (Female): It is going to be young I think very young kids school because I have two younger children, and they will often come home and something and be very passionate about it, and it could be a cause or something. So, you know, did you know what it is mom, like that, you know, you might have known bits not convinced about it, but I think, you know, they might not be able to do anything about it, but I think they can make parents more aware, or even make you have a conversation with them about it of what it is about. It is like it is very hard to fob for a 5 to 7-year-old off and they are you know, tell you that you should be, you know different things. I think, I don’t think it is the only way to go, but I think it is one avenue definitely like the very young, someone gets someone in, maybe into the class, to give us some kind of talk, making it obviously child friendly, not just sitting there, you know, make it fun. They will come home with that information, and then want you to know and want to tell you about it.

Dictator 1 (Male): So, it is the education thing that you talk about.

Dictator 3 (Male): I have a 5-year-old kid and he is in 4th class in school in Clonee and they got a talk about what is good and what is bad for the body, and this 5-year-old kid comes home and says “Dad, that’s bad for you,” and I am going to say how does a 5-year-old kid gets taught about that, and that is where it was coming from.

Dictator 2 (Female): They do, because they are kind and passionate about things I think and they want you to know and to do so then they will go back and tell the teacher that we did this in our house last night. Do you know the one to do that and I think it is a very simple way, I do think you need people that are interesting for them to listen to going to the schools and start there. Obviously there are loads of other areas the media, everything is as well for everybody, but I do think this one way and I think would have an impact, but if they you are not going to listen to a young child you will try going on and on at you, you’re going to do the thing so you go and talk to them, and that is nothing to do with influence.

Dictator (Male): They will influence your shopping

Dictator (Female): They will, going around the shop with me, definitely, they do influence

Dictator 6 (Female): I think five years is a very short time to see a lot of change, but we are making the you could make early ?? information to start the change, but I do not see it making change, I think the change would be generational. I think the change in people’s behaviours to address what they are trying to inform us, I think will take a lot of time.

Dictator 2 (Female): But the change itself is also a problem. Remember we were all told eggs, no no, not more than two eggs a week, and doctor so and so said this, but then doctor so and so said no no actually that’s is wrong. It is constant Oh, coffee no, of coughing, you know, of this or this or whatever it is, the constant yes / no change from authorities and people, and the food pyramid is changed now, yeah, exactly.

Dictator 3 (Male): Is there a need then to, this is, do not shoot the messenger, one, that we have to have a department of health or HSE, the people have confidence it because lets be quite honest about it, the confidence factor within the HSE. So that needs to be worked on that we are the authoritative body or whatever and people have that trust, now how we get around that I couldn’t tell you but the trust in the people within the organisation, but the organisation itself which is responsible for health, need to be the same to build up public confidence.

Dictator 4 (Male): I think we need a really big shift because you know, diet and chemicals, you know have pushed the planet to the edge you know, so, I mean, something big does have to happen, and you know, it had to be we have to realised things like eggs you know from where I come from, diary, meat, all that stuff is lethal for you, and there are studies out there that show it because there is so much advertising has been going on for so long that this is a normal diet but and same with chemicals because they have had their own run for so long, with so little blocks put up against what they are putting in products and food and all that, so we need to call a major halt and really say you know this has to stop right away.

Dictator 6 (Female): I think that there are concerns, or at least I have some concern about the way we address “health”. We seem to put health in with medicine, and they are not the same thing in my book. Health, health promotion help disease prevention, is far more important particularly in this arena than it is with meds and treating the after-effects of all of these chemicals, and I think we as a society, because maybe of the influence of medicine or money or whatever seem to put medicine as the umbrella that fits over all of these, and we do not segregate out health, health promotion and disease prevention as a bigger or better way to look at the world than we do about putting billions and billions and billions into treating the stuff or treating the diseases that all of these chemicals have caused, and I think it is a massive change in how we look at life with health as the primary focus, not as medicine.

Dictator 1? (Male/ not Maurice ): Great point.

Dictator 3 (Male): The problem with that is that it’s the pharmaceuticals they are driving. It is not in their interest to move into a preventative field _______ (29:23)

Dictator 2 (Female): It is like some things you cannot find in a health food shop _______ (29:25) anymore because you know it is not licensed, but they have not got the money to promote this, but it has been shut down by pharmaceuticals, so the rise in after natural products available that you can’t buy….

Dictator 3 (Male): _______ (29:42) This is bringing it down to a microscan, if you look at even housing, people do not have the option to have a small garden or anything else, you know, people would encourage you to grow things. I know when I was kid, the grandfather had an allotment. Now there’s nowhere where they can develop an interest in it.

Dictator 1 (Male): Okay, so you have mentioned a lot of things there. What seemed to come out was a lot about having an authoritative voice about information and there’s another question here about information, which I am looking at, and we have already kind of touched them, how you would like to get information in the future, you know, out of this study or whatever else on biomonitoring, it seems that we have touched them because you have mentioned about you want authoritative and you would want to be able to interpret and you want it to happen in early age well with kids and the rest of it.

Dictator 6 (Female): _______ (30:30) And what about like on-going I am going in from progress kind of reports like it is all well and good to say yeah we love have answers in four years’ time, but we all would have forgotten about this where as if we got some feedback, you know, on going, this is where we were at, this is where we hope to be at that it might keep people engaged too and interested.

Dictator 1 (Male): Yeah, okay, well they mentioned a website for that. I suppose in general, it is about you have to go looking for information, don’t you, and it seems where do you go for information that informs you in a way you want to be informed, you know, that you are happy with, and maybe you can have trust in, and it goes back to the point you raised about independence trust and the rest.

Dictator 3 (Male): I think the old fashioned way of paper, we have the referendum, we have in every house in the country that is 20 or 30 page booklet in the letter box explaining the referendum, maybe a the four page booklet…

Dictator (Male): Yeah, I agree, yeah

Dictator 3 (Male): …giving the details of where to get this online and if you’re on run any social media, what address is there, so the people will then know where to go looking for the information.

Dictator (Male): Billboards, yeah, reliable, simple.

Dictator 4 (Male): Go back to the old fashioned way, as I remember growing up as a kid, every time there was a ballot on or a referendum or an election you get it was like and nine times out of ten, family members would fling it in the bin, why can’t you cut it down in size and just give the basics.

Q 11 Dictator 1 (Male): It goes back, there was an earlier question that we had which was know what does certain groups need to hear what seemed to come out what you were saying there is the young can get information quite quickly, but as you get older, you have a pattern of accessing information, you buy a newspaper instead of going on online, maybe still, or you don’t really use the internet, so is there an issue about the people maybe need to hear this most or not hearing it enough, or is it an issue the young who are hearing it but are so young that they cannot communicate properly to older people and is it a mixed kind of communication method that we want.

Dictator 3 (Male): It’s a mixed communication, you need to target the different The difference, demographics that you have in society, and I would agree start with the kids and in primary school, but that is you are looking at a generation away there. It needs You need to then go out to teenagers, so you go on Facebook or whatever there, and the point is sometimes when you get up to the people in their 60s likely lately, they actually prefer to get a small it on a piece of paper.

Dictator 4 (Male): I think if you You do a leaflet drop, but that should not be just one department be one time, the booklet that goes out should be something like he said, it does not go in the bin, it is something that you want to retain, so there should be other information about electricity, water, everything, so you keep that as a little manual in your house and maybe you put in doctor’s numbers at the back and everything anybody else, but it had to be a multi-departmental approach.

Dictator (female) You’re manual for living.

Dictator 4 (Male): It is so had to change people’s behaviours, you know, like diet, like water use, you know, people turning off their tap when they are brushing their teeth, some people just cannot get it in their head, you know, they will just leave the, you know, water going down the drain, so hard to get people to change their habits.

Q 12 Dictator 1 (Male): And how do you get behind that?

Dictator 4 (Male): (still Maurice talking) Because, it does seem to be true that.

Dictator 1 (Male): We talked a lot about getting information, looking in the right place for information and getting information together and get them out to everybody but then, you know, when it gets to some people, it goes over their heads or they decide to ignore it, or...

Dictator 6 (Female): There usually has to be an incentive or something.

Dictator 4 (Male): the calorie count in like McDonald’s and restaurants. I have heard a lot about that people noticing knows you know there is 1500 calories in that muffin, you know, holey moley.

Dictator 1 (Male): It is that, I feel like the point you made then, it is really about awareness at the point of when you are consuming, yeah, whatever you buy, or the point of contact, yeah.

Dictator 5 (Male) (Female): In the future world, I mean, you asked us to think about the future, I mean, I do not want to see us all, well I an antiquated anyway that is the stage of technology I am at, but I see people with all of these fancy phones, you know, you can scan a phone over something and the information comes up or, you have your phone beside you when you come to this particular section, it’s cosmetics, and you can dial in your own cosmetic with you whenever, but, I do not know just something like that, but I also think that going on what Colm Corey was saying to get that information to translate into changing behavior. There has to be some sort of an incentive, and I am not saying money, but there has to be something for someone to buy into it that they feel like they are gaining something by doing it, and if that is not applicable to them, there is going to be no way that things are going to change chance.

Dictator 3 (Male): you have a very good point there, if you pick up a product and I don’t have my glasses with me I can’t read the lable.

Dictator 2 (Male): And even with these, I still sometimes cannot read it, it is so small.

Dictator 1 (Male): And influences that is the social media so much these days. Is that something that is not really used enough that you do not have people who stand up and say, you know, who have some reputation from what reason or other and say I believe in using this, I don’t use this. Does that have a part to play in biomonitoring that people should be, you will follow some people’s views news, we talk about trusted people and information, but do you need to go so far as to have credibility, is an issue, not it, yeah.

Dictator 2 (Female): I would believe in somebody who has lots of followers because if I know they are going to say something.

Dictator 1 (Male): So you have seen behind it. It is a bit like the point you made that the truth will out without kind of thing that people…

Dictator 6 (Female): Give You need transparency like even for example with bloggers the way there was no kind of policing with them before and now they are promoting something, they have to say hashtag sponsored, so you know you they are getting paid for that, so we need to know like if people are genuine about it.

Dictator 3 (Male): I think that is why you need to be linking with facebook, Google, to have a sponsor as a state, I and get the advertisement, but to get that you have to have credibility in the government information services that you get reliable source.

Dictator 2 (Female): Well, if you take it back, sorry Michael, if you take it back to the climate movement if you like that has happened, that happened on social media. Just a young child, 15 or 16,

Dictator (Female): when she was is 14 when she started, she is 16 now

Dictator 2 (Female): …which is so young to have the motivation of the idealism that there is in young people. This is our world. You are going out on, bye bye, but this is our world, and look at the mess you have made, they are motivated, and it is in the social media, and that is what captured everybody. It was not papers, it was not television, it was not….

Dictator 1 (Male): It was the girl’s credibility,

Dictator 2 (Female) : Exactly, her truth, and her credibility

Dictator 2 (Female): And I suppose as well, it’s It is an EU kind of directive as such as well, you know if it was is happening across the board as well, you would be more, you know with social media and say for like that girl with the climate change, it is everywhere, you know, it is not just in Ireland, these are your legislations and this is this like everyone knows to wear sunscreen, you know, in any country, I know there is a product of chemicals in it, but you know, and it is widespread.

Dictator 3 (Male): And is there any other EU countries, that have come up with a model or are we all in the same place, any of them more advanced, have they tried some of these and have not worked.

Dictator 1 (Male): I might answer that after we finished the session because I do not want to leave the questions, but there are some that obviously there is a market leader in every country on something, and it is no different in biomonitoring.

Dictator 2 (Female): But listening to the BBC the other night I on world news and they were talking about in Sri Lanka, the number of suicides is off the scale, and the lives have been taken through pesticides. People are taking pesticides, just the only way out, and no money, and for once, the government has stepped in. And they have put an advertisement that you are not allowed, just willy nilly get, they have closed it down because it was such a huge numbers and that was the only government thing I ever heard for, great, sorry, government is doing something, yeah.

Q 13 Dictator 1 (Male): So, do you believe the results of the biomonitoring, especially this initiative is relevant to, do you think they are relevant here in your everyday life? Do you think there is enough of it?

Dictator 3 (Male): Not enough, there could be more.

Dictator 5 (Male): Then, in someways I think there is too much sometimes, like I have come quite ambivalent towards the whole thing, like oh this is going to kill you, this is going to get inside your brain, in your toast there’s carbon and you are going to get cancer, and that keeps changing and it is just like too much information for me, and I just do not want to watch it, it is like either way I am going to get something clearly, so what is the point, you know?

Dictator 1 (Male): Okay, so like, information overload can lead to apathy kind of thing, so it is an issue fully about targeting the information.

Dictator (Male): I do think you need to target more priority substances than eighteen. There are probably 18 toxins in a smoked rasher. _______ (40:20).

Dictator (Male): Are you looking at any specific target groups?

Dictator 1 (Male): Sorry I’m going to close the window, one second, lets just close the window.

Dictator 3 (Male): _______ (41:00) … that would have an higher exposure level ???? we are very conscious that we protect our planet _______ (41:05) staff from the environments we send them in to ?? but you go in to any one of the hospital for instance and this comes back to yourself and Temple Street, there are cleaning products that we’re exposed to every day, antibiotics, wipes, you know there’s a greater level of exposure to health care workers _______ (41:20) or emergency workerts thn there would be say for than it would be safer for a person may be working walking in an office 9 to 5 might find.

Dictator 3 (Male): Particularly in hospital labs and histology, my wife works in histology in Vincent, I mean formalin is highly carcinogenic and xylene.

Dictator 5 (Male): Sorry what is histology?

Dictator 3 (Male): It is study of cancers, body tissues, things like that, my own work I used quite a different organic solvents, by the bucket quite literally.

Dictator 2 (Female): Which is But sure the room we are sitting in. The amount of formaldehyde in all the furniture. Sorry, HSE, I do not mean, it is everywhere, in my home, it is everywhere, all our furnishings, we are breathing it in, paint air conditioning system, curtains, everything

Dictator (Female): and at least in the labs you do kind of occupation exposure limits, you test it, whereas general public population do not, like if you are going for biopsy taken, it will go into formaldehyde but like they are not testing the limits, we do in the lab because we are using an it ongoing but like the general public don’t.

To 1:04:31.61 on Cora’s recording on her phone.

Dictator 1 (Male): Okay, so look, we might wrap up. We have got through the questions and I think we have navigated them well, and we might carry on with the few other conversations that we have got through the pain part of the questions, so thanks for attending and everything else, but I might give you some perspective maybe on the study. I think it is a tremendous project because what they are doing is they are looking at chemicals of interest or concerns, depending on which phase they want to use, there are either old chemicals that have long track record of you know disease, illness, end points that we would call them and then there are also starting to look at new and emerging chemicals and they are looking at far smoothing product lines, say like an use of soft plastics in plastic bottles and rest where the chemicals may migrate into the water that you drink, you know, there is a small sweet of chemicals compared to the thousands that are out there, and there are only so many chemicals you can use as a marker, they call them biomarkers, this is what biomonitoring is, so they really just kind of looking at one chemical as an indication of exposure and maybe for that group, the group though is full of Europe’s experts on everything to do with, even uptake of chemicals, and there is a new field called ecogenetics, which is about changes in the body even from small exposures, and what the future might bring is that they may be able to max early effect of markers so they can tell when something is happening earlier because of inflammatory markers in your system, so this goes more into the clinical world. So, I have wound up now and this can be some kind of information. So, it is quite fantastic that this project is started, but it is only limited, but it is brought on well together with people that are involved in very fascinating science, and the whole issue about human exposure measurement, there is a field called Exposum, which is the, which is whole life exposure from birth to death, and it is very big in America, Exposum, which is a very big field in America, and it is about looking at life course and key interventions at all of life, so from your early age development up until late years and early years, and it is kind of so critical, so the group has also has noted the early life exposure and mixtures, to two issues that are quite phony because you mentioned that we are involved in so many chemicals in our lives, at work, when we got home, and everything else, and it maybe even hard to measure, what we call the level of detection that you are in, but when they mix with something else so they are going to create some other kind of reaction, so it is like that known knowns and the unknown knowns, you know that quote, but to me, it is a great starting point. It is a brilliant project. It involves 26 to 27 members states, now Ireland is involved in a small way, we are small country, but we are in the tent, if you like, and we are trying to learn to manage a small group of chemicals, but the priority chemicals lists hopefully will move on and on to try and catch up. So, what I think is good is that the old daters say on mercury or lead, they are mining the whole of Europe’s status on this and they have a website called _______ (46:16) of the European Environment agency, Joanna mentioned it and they are going to pool all of this data and then they are going to analyze it, so the good studies will be kept, that studies will be put on the sideline, and then they will look at those response, relationships, and they will look at health impacts, and the idea is it will inform risk assessment, in other words, what level do you want of this chemical and this food product, or this chemical in that cosmetic product. So, it is trying to look backwards to look forwards, if you get me, so this what research does.

Dictator 3 (Male): Basically they take the negatives and turn in to a positive.

Dictator 1 (Male): It is about taking all, so what they are going to look at, they are going to look at all chemical data that has been looked at so they do not have to render new studies, and then they are going to start new studies or new chemicals so that we are no falling behind the curve too much, and the plan then is that the information from this flows into the European Commission, flows into DG Research, Directorate-General Research, DG, to represent grow or whatever it is called, and to enterprise group, and European Food Safety Authority, and the other risk agencies, European Health & Safety Authority, so that there is a learning in terms of what the exposure levels are. It is a small initiative in money ways, but it is linking with work in Canada, in Japan, and other countries where there are big biomonitoring products. The biggest biomonitoring product that I feel is the National Health & Nutrition Study in America where they follow large numbers of people over four-year cycles, and they look at their exposure to tobacco, whole range of chemicals, and then they look at whether it is relevant to certain sectors where there is a certain ethnic group or a certain age group, and it is really informative because, you will say, look that chemical is actually going down because we put a lawyer in place, so even for enforces, it can be really valuable because you can see that reducing certain point of level of standards of on mercury, and you see mercury exposure goes down in the population, particularly in young people is what you are looking for, and then you can see over time subtracted, so it is fascinating, well it is, to me, it is tomorrow’s world’s kind of science that we are moving towards now, and I think that is why Ireland is involved because we see that it is not just going to look at chemicals in blood, it is looking at inflammatory markers in blood, and it is going to start putting together questions, and I really like, I think the Woody Allen film where you went into the blood or submarine. It is turning the outside world inside, so like chemical exposure and turning inside out is they way I would put it. But what you have done today is you have kind of, you have reminded us how important it is to give people information and to listen to people, and that there is a huge amount of interest in environment health as well as student and what you seem to be cry out for is information that you can trust that is timely, and that is coming from a valid source, and you can get immediately when it is relevant to what you want to do in your life, you know, so, I want to thank you but also have we missed anything.

Dictator 2 (Female): Well, that is one of the questions to ask the group, have we missed anything.

Dictator 6 (Female): How much data do you think should begin to the public when making them aware of the results, have we covered that well enough?

Dictator 1 (Male): Not really maybe.

Dictator 3 (Male): There should actually be more of these forum groups not only people are working out of HSE (50:11) let them know what is good and what is bad, I would have 15 to 20 members a day coming in, and they are all eating different foods. and before today I could not go up and say oh by the way you cannot have that because it is not good for you or the way that you cannot have that because that is not good for you, but now with some learning I can say, do you what is in that.

Dictator 1 (Male): Okay, and we should say there are legitimate sources of information, maybe there is not enough information there, but the large agencies like the EPA, HSE< like the Foot Safety Authority of Ireland do have websites and links, often to European groups and projects they are involved in, and if you are looking for, you know, like mainstream research findings, that where you will generally find it, and it is not just a web search on whatever platform you like to use.

Dictator 4 (Male): That is coming back to the point that you need to inform people that these agencies actually exist and how to contact them, so leaflet in the door goes back to I think is the most effective way to communicate in that.

Dictator 2 (Female): But I don’t think we are going to get absolutely everybody, some people would have an interest, but if you can increase, you know, people’s awareness and people’s willingness to buy into it, and that is the one to be something, everybody in the country is not going to suddenly change reviews or liking about it this is about increasing awareness and that in turn will increase, you know, future people coming up and . . . .

Dictator 6 (Female): I supposed to open up as you say discussion groups to the general public and get a broader range of people, I know, we have quite a different range of backgrounds here today, but I suppose the more vary, the better.

Dictator 1 (Male): Those things I think that, it is a personal view, so it will be taken out probably when I do it, but it is the issue of trying to not have a fear factor in the way we give information because people can react to every single message, and over-react, and then they can ran away from food group or away carrying on their life to probably consume with the way the exercise even maybe, and actually it is not really, it is only something that would need to modify, but not give up altogether, you know, so to me, I think it is very important that we convey the messages in the right way, but in a proportionate way people can take the common sense out of it, you know. You kind of say you can see it that they do not over-react, and that is the skill in itself, I think, the way you communicate messages to people.

Dictator 2 (Female): Also, there is that thing that you know people feel, why are you telling me this, it is at a point of becoming nanny state, I do, but you know this saying, do not interfere with my life, you know, it is advisory, but people sometimes do not even that, just do not come in to my life, a lot of this, you know, why is this nanny state or being perceived as that, but it is not, why, some people might think.

Dictator 5 (Male): The U.N. Summit a few years ago, and their slogan was do one thing, you know, so you are not overwhelmed, you know, like smart, you know, advertising or smart sentence, like that, you know, do one thing, that is how you are starting, do one thing, you know, stop using so much chemicals, you know, poisoning yourself, you know, do one thing.

Dictator 1 (Male): You asked about other countries that are leading.

Dictator 5 (Male): Yeah.

Dictator 1 (Male): Slovenia is I think the first country, as you have mentioned Czechoslovakia, Slovenia introduced biomonitoring legislation, which meant it had to be done, so they became quite a leading authority on doing work on biomonitoring.

Dictator 4 (Male): _______ (54:25) If you have a good education in every society you have influence _______ (54:30) social inclusions and you educate the person there one day just awareness then they will pass that and you will get a ripple effect that will be given to people in the right language and the right environment.

Dictator 1 (Male): So, I am not actually into give any comment. She has?

Dictator 6 (Female): Yeah, no, I just ask from _______ (55:15) I work in the research area in the EPA, so why we are not involved in the research, we obviously provide funding to researchers around the country to do research projects, some of which will be around biomonitoring, but even just getting our perspectives today on the communication of the results and things like that, I mean, it is something that is certainly I will bring back just in the ongoing work that we are doing around, I mean obviously a lot of our research will be focused around how is this going to form the policy or how is it going to inform government, now obviously, a lot of it as it would be for the scientists, but also to public, and maybe they are just not doing that as well as they could be, scientists I suppose by their nature, they are scientists, they are not communicators, so it is a difficult task, but potentially we need to you know help come along in that a little bit and try to get the information out there to you in tailored timely way that you can use for your own purposes and your daily life, so I just thought that was, it just kind of brought a little bit more home to me that probably we could do a little bit better on the communication site and things that the money that we are putting into research and the work in research that is going on, but it was really useful discussion today. I thank everyone for their input, it was great.

Dictator 1 (Male): Corey, do you want to add anything? Have we missed anything that you can think of?

Dictator 2 (Female): Yeah. I think we fairly much covered all the questions and all the ground, unless I will ask again if anybody thinks we missed anything would you want to add to just get off your chest or.

Dictator 1 (Male): I think it has been a great conversation from my point because I think that you can sometimes work in the vacuum, and it is great to meet real people with real views because as I said to my friend earlier, you know, if you are not going to change things for the better, then why you doing what you are doing, you know, and

Dictator 3 (Male): Just look in to where I am, I am stuck in an office all day and it’s like send an email after emails after emails, to all of these different companies trying to to get their orders in for the following week and it’s like why am I sitting in an office like this where I can go out, like we all are here today, we are all having a conversation, and that is what really kills me when I am in the job, means talking ______(57:47).

Dictator 1 (Male): Okay, is there any closing comments or remarks that you want to make or before wrap up and release you.

Dictator 4 (Male): The best way to get information I believe that is go back and tell my missues, it is a secret.

Dictator 1 (Male): Very good, thanks very much, so I think we have lunch. Thanks, really genuinely, thanks a lot, it has been great. Find a way through emails and then this is what it is all about, so, we will be pushing together what you want across the board, and we will bring all this stuff back and try and make it a reality. It is funny to think about the bracelet. We nearly did that study, but there was an issue about ethical approval. We are going to get yourselves to wear a bracelet so you could not quite swing it, because it is quite nasty because you have to be worried about the fear factor to it, Oh God. A lot of the issue about biomonitoring is that when you take this chemical samples is actually then communicating the information to the group because you might have a clinical, you know, you may have when you get older you always end up with something, you may have something that you are predisposed to and that communication to you, and you can also freak out if you hear you got something, _______ (59:15). So, it is tomorrow’s world, some of us are old enough, there was a TV program called Tomorrow as well, this really is. Thanks a lot.

Dictator 4 (Female): And by the way, thank you very much. We have got your gift vouchers here and there is an explanatory leaflet in it.

Dictator 5 (Female): And you can only go to chemical free.

Dictator 1 (Male): Thank you.

**Transcript of the UK focus group (October 2019)**Chemicals FG 1.10.19. Part 1

**SUMMARY KEYWORDS**

chemicals, regulations, autumn, people, legislation, antibiotics, information, concerned, pollution, problem, environment, plastics, public, pesticides, water, manufacturers, pay, exposures, bit, government

**SPEAKERS**

Female Member 2, Facilitator 3, Male FC Member, Facilitator 2, Facilitator 1, Lady FC Member, Lady FC member 2, Male Member 3, Male FC member 2, Male member 4

**Facilitator 1** 00:00

aims to investigate the actual exposure of chemicals, you know, just

**Facilitator 1** 00:05

even in Europe.

**Facilitator 1** 00:07

And with the data from this project, we can inform policymakers in designing new chemical regulations for enhancing or evaluating existing policies. So this is really important to us to have your views that we can feed back to this project. And before we delve into this interesting discussion, I'd like us to introduce ourselves. Tell us why you decided to participate in this discussion today. And your favourite thing about autum.

**Male FC Member** 00:38

I am [name] as most of you probably gathered as I haev been rabbiting like crazy. Hmm. I've been worried about chemicals in the environment for a long while, as I say knew about fish with high levels of mercury in a long, long time ago. Finding out and I will say life has probably most people here lived through the problem of farming chemicals leaching off the land and into the river. That was a big worry when I was a lot younger and and it just seems to go on and on and on. And very little is done about it until it becomes a major problem ah which I find very disappointing.

**Male FC Member** 01:37

Yeah, if something should be doing being done even only on a small scale as soon as a problem is found, because when you start doing something about it even on a small scale, then you start learning and the more you learn, the easier it is to cure the problem, if not completely, then to a point where it's not going to be a health hazard. So I find the idea like chemicals I find it a very important subject. To me personally, it affects my life and it may even be affecting how long Im gonna live? It might be taking years off of how long I'm gonna live without me knowing about it.

**Facilitator 1** 02:30

What's your favourite thing about autumn?

**Facilitator 1** 02:33

Your favourite thing about autumn,

**Male FC Member** 02:35

Autumn, change in the leaves all the different colours I would like to. I like to go to various places like North America and Japan where changing leaves is much more noticeable, more vibrant colours. I mean, it's nice to see all the different colours in this country but it can be overshadowed by other countries. I would like to be able to see things like that but they live with the fact that I can't.

**Lady FC Member** 03:20

Yeah, just what are my favourite things about autumn!

**Facilitator 1** 03:25

You need to introduce yourself. And why you decided ....

**Lady FC Member** 03:30

I had a lifelong interest in, I suppose keeping healthy and I think that extends to the environment and myself so recently I've had much more interest in medicines antibiotics The side effects because of having had you know an ongoing lung condition. And I am increasingly concerned about the, I don't know, it's like it appears to be almost the un-licenced way in which the manufacturing industries have launched on us, things which don't appear to be regulated. Hm, we are much more regulated in this country through the EU then say in America, Americans pretty appalling the way they the chemicals seem so let people get away with.

**Lady FC Member** 04:35

I think there's sort of issues about the wildlife, for example, the illegal use of pesticides, which are actually killing bees, and that we don't have systemic pesticides they've been outlawed. But with our departure from the EU is that concerns me greatly that some of those extreme measures. We, what are we going to put in place? You know, and I'm hoping that this discussion group is going to start informing that process about actually the regulatory systems that we as a country have developed through I think this is this chemical strategy is proposed. And so it's it's doing that. So it's, it seems like a really crucial time. For us, we've already got a lot of good things that are going on, but we still got a lot further to go, especially with the sort of crisis to do with plastics and pollution. And the sort of it appears, for example, aircraft aren't really regulated and they're dumping a lot into the atmosphere. The in terms of 'particulants'.

**Lady FC Member** 05:54

So this this, those are all the interests I've got. Ive ... to present because of City Airport proposing an expansion and now in London, it's it's a problem. So it's the need for more regulation.

**Lady FC Member** 06:16

Autumn, dont really like autumn, actually. But what I do like is, I realised last night I actually enjoy the fact that evenings are getting it's getting dark quite quickly. So there's that sort of building up into this sort of making yourself bit snug at home sort of nesting in so I like that feeling. Yeah.

**Lady FC member 2** 06:45

Im [name] I'm gonna sound really, really boring. I feel like I'm a bit ignorant. I'm a fitness instructor. So obviously I'm concerned about health but I feel like I'm a bit ignorant with with with regards to chemicals and everything like this. But, obviously I'm concerned about the environment I've got kids so yeah just I would just like to find out more really and I feel like I should educate myself a bit more. And yeah the same day and I love the colours of the leaves. When you when the leaves from the fruit crunching through there.

**Male FC member 2** 07:32

Im [name], ive been on the peoples panel a few times. Ive been asmatic for a while. I got interested in this through Brexit actually. The effect that Brexit is going to have. From the point of view that a lot of the protections we have been put in place by... (poor quality recording) ...

**Male FC member 2** 08:15

When we come out the effect that it will have, alreayd last week there's a ,,so you need to say of getting rid of red-tape, but it is red tape that protects us. But a trade deal...which presents...chickens mixed with chlorine which we eat don't contest ...yeah .Got interest in this amongst other discussion groups have been involved in interested to find out more. ( poor sound unable to hear all the words) Autumn - the start of the football season.

**Female Member 2** 09:40

My name is [name]. I'm a retired civil servant. And at the moment I work with challenging adults. So I deal with chemicals every day I work. How to keep them safe from the lads and make sure we have some of them that ... you put a cup or glass of any liquid down that and they will take it up and drink it. So you have to be very, very careful with that. So that's part of our training, chemicals under lock and key at all times. So I'm used to that.

**Female Member 2** 10:18

And sometimes we watch them and you know when they want to pick up something to drink. 'cause they'l bewatching you with your cup of tea or whatever and before if you just have to take your eyes off or if you've taken them for a walk, and they see the neighbour query, you know, opportunity and they see the neighbour with their kitchen door open. They will run in and grab that cup of tea. You have to be very fast to catch them you know, so that's something to have to deal with. We're autumn is ocncerned... Where chemicals are concerned, I'm worried about the amount of chemicals we are ingesting into our bodies or it's in our foods. Unless you think even if we say we produce our own foods, what you're buying in the shop have the chemicals in it. So what do you do? How do you get around that is typical the chemicals are in the food we're buying in the shops, because of what they're feeding the seeds and the plants with, they put in the chemicals in. And then when you are going to prepare it, you can add more chemicals to it. And I think there's something that has to be looked at, and something has to be done. Otherwise, our children will not be here to benefit anything on this planet. As we know it. It's simple as that. Now we're autumn is concerned I love the changing of the seasons I love spring, which is bringing in new life. Summer when you can enjoy the sun and take the whatever benefit you get from it, and autumn the changing of leaves or the change from different colours. And you know, it's just magic as far as I'm concerned and then I love winter. I love winter. I love winter. Thank you.

**Male Member 3** 12:28

Yes, I'm [name]. I've worked in mental health. But before that I worked in environmental biology and chemistry, and think chemicals are marvellous, amazing sayings. We've got things like chemotherapy, preservatives, flavourings, all sorts of things like that, and we've got pollution. And we've got a lot of chemicals as a sort of in between with, with good and bad things about them. But one of the earliest bits of research I was involved with with chemicals was lead in petrol. And at the time, we were trying to prove, well we were monitoring lead in the environment and in people's blood and children's blood. But the scientists or the scientists were trying to prove that lead was damaging children's brain development and producing triethyl lead, I think it was producing companies were trying to dirty that and say oh no and that's not happening and it's not relevant. And I think that's relevant to a lot of the things today. I wonder what's changed in that time. And I think one of the things is people's trust in scientists, and this is very worrying we're sort of in a 'Post Truth' situation. You know, how much people know who to believe and where to get their knowledge from is an important thing.

**Male Member 3** 13:59

The thing I like about Autumn is the spring flowers because autumn isn't really a time of death. You dig under the leaves and the bluebells are already coming up and the buds on the oak trees already swelling. You know its jjust a rest and things are growing. Thats whats amazing how many plants grow in the autumn.

**Male member 4** 14:22

I'm [name]. I'm a retired business consultant. I like autumn because it's for me, in my mind at least a change of season. And I'm looking forward to what I hope will be he lots of bright, cold blue sky days. Whether we'll get any this year remains to be seen. This particular autumn is heaven on earth for me because of that shirt that [name] wearing: the Rugby World Cup

**Male FC Member** 15:01

Ive got a black one of these

**Male member 4** 15:02

Oh, so I'm glued to the television from 5:45 most mornings. It's driving my wife up the wall. It'll be over in a few weeks. I tell her.

**Male member 4** 15:18

So far as pollution is concerned, I wouldn't pretend as I've said to [fascilitar] to be an expert on this. But I have a view as a consumer about all the different kinds of pollution that we're subject to. In a former business life. My wife and I lived in the US for 10 years, and in China for seven and a half years. In the US, despite its reputation, the level of visible pollution is negligible. Maybe in a few cities that are called Rust Belt towns, like Detroit, for example. You can taste it sometimes in the air. In China, it's very, very different. This is a country that economically is struggling to get from the 18th to the 21st century, in three or four decades. And so in my mind, there is a direct link between economics, the activity of economics and the levels of pollution and the types of pollution as well. In the north of China, where a lot of the heavy industry is based around Beijing, for example, and going up towards the Mongolian border.

**Male member 4** 16:44

There are many, many days consecutively when you never see the sun. It doesn't look like that sky there where the cloud is fairly low. It looks like what smog used to look like in London in the 1950s that's what it looks like and it sometimes has a kind of orange sulphurous glow to it. And so it's very common in Chinese cities to see pedestrians and cyclists and moped riders with face masks. And occasionally I wore one myself all I did feel a bit self conscious about it. And I think there's a very good reason in China why people do wear face masks. Were they do any good? I've no idea. So I have an interest in the subject. I've read a few articles about it. I listen to the radio and read newspapers and so on. And I think it's a very important science of the next few decades.

**Facilitator 1** 17:56

Thank you

**Facilitator 2** 17:59

Im [Name] I work for Public Health England as a public involvement officer, I forgot then! And just.. I think.. like a lot of people here I like the colours of autumn colours.

**Facilitator 3** 18:24

I think you said earlier I'm [name] Like [names] I work for Public Health England but I'm in the chemicals part of Public Health England, PHE has a huge remit. Chemicals are obviously part of my day job. And also part of my job at home. Bit like you were saying. Autumn: I like autumn, as when you opened the back door on autumn morning. The smell is very different to other seasons. And of course like you said the garden is or another you said the garden is still growing. There's still things in the garden. I think thats just wonderful.

**Facilitator 1** 19:02

thank you, I'm [name] do the same job as [name]. And my favourite thing about Autumn is Halloween. That's it for the session. So I have to pass on to [fascilitiat 3] to carry on the other half.

**Facilitator 1** 19:24

So thank you all. I mean, it was really fascinating to hear all your different thoughts, and we're in our thoughts are influenced by the work we do by things that are out there. And as [fascilitator 1] said earlier, the focus of the next hour and a half I think we've got now is really to ask you or to give you a little bit of information from what Public Health England does a bit about chemical regulation, and then to get some more views from yourself because the purpose of a focus group is not for us to sit here and talk but to really to understand your views as citizens, somebody mentioned that your scientists are that there's less confidence in scientists. And I think one of the reasons for that, this is a personal view, is that we often don't ask the citizens, what do you think we make assumptions on your behalf? So this is really important for us to get to understand what you think. Please go ahead.

**Male FC Member** 20:28

I think another problem with believing what scientists say is that who's paying them? Who that who they work for?

**Facilitator 3** 20:44

I will come on to that when I talk about what PHE does.

**Male FC Member** 20:49

You can't guarantee that what they say is not biased.

**Facilitator 1** 20:54

'They' is a .... yeah [laughter], I mean, I totally understand what you're saying. So, again, from a personal point of view, I used to work in academia. So I wasn't academic scientists and not affiliated with government. I am now a civil servant, as we all are, and I work for Public Health England. Now Public Health England is what's called an executive agency of the Department of Health. What that means is that the Department of Health, government pays our salary. But we are supposed to be scientifically independent. I could discuss this for hours, you know, how can you be totally scientifically independent if the Department of Health are also inputting into your business plans, for example, but we can perhaps get some time. So Public Health England, I'm only going to talk about one small part of PHE and that is the chemicals part and [names] work in the toxicology department. So we look at how chemicals affect our health and we look specifically about public health because that's what we do. So as you all know, as many of you already mentioned, in fact, all of you already mentioned that chemicals are everywhere in our everyday lives, we can't avoid them. And in some ways, and I think you said, [name], that you know that chemicals are also useful. So we have to have that balance.

**Facilitator 3** 22:23

We need chemicals for things like antibiotics, for pesticides, for the seats, were sitting on the tables, you know, the computers, etc. So there are good things about chemicals. But of course, from a public health perspective, what we are doing is looking at how chemicals potentially affect our health. So somebody has already mentioned air pollution, there's a lot of work going on air pollution on particulate matter how does particulate matter affect our health? How do we reduce those exposures you may have heard about water pollution. I think, [name], again, you mentioned lead. And I'll come back to lead because lead is a classic example of how we've tried to control pollution. But there are many other things that are potentially in our water. And therefore when we drink water from our taps, we may be exposed and that's when we come on to how things are regulated. In the UK, we have a lot of land that has been used in the past for industry, so therefore, there's potential contamination on the on that land, how do we then use that land, we're not such a big country that we can afford to leave land, you know, to, to them, if you like, naturally naturally recoup, we often use what was previously contaminated land, but how we do that has to be well understood. So what we do in in our group so I have a small group of toxicologists, and we assess exposures to.. potential public health exposures from the from water from contaminated land from waste. We have a different group who looks at air pollution. Air Pollution is is a separate topic on its own. But that's what we do. That's what we do in our department. And our mission is to protect public health. That is what we are paid to do. And I was discussing this with one of my colleagues yesterday and I told him, we wouldn't do this job for the money because we could work for chemical industry, for example, and earn a lot more. Quite honestly, we're quite passionate about ensuring that the public is protected as far as we can from potential chemical pollutants. So what do we need to know? In order to do that, we need to know which chemicals are in the environment and in our food, for example, or in cosmetics. And we need to know how much is there and how much are we potentially exposed to. The other bit of information we need to understand is what the effect of those chemicals are on the human body. So because we are a public health agency, we we don't deal with environmental pollution we don't deal with as in to the environment, we're really thinking about public. So that's really our mission. That's what we're here to do. Very, very briefly to talk a bit about chemical regulation. Some of you mentioned that in in your introductions. And so we have regulations, we have policies, we have legislation, and we have numerous pieces of legislation that deal with potential chemical exposures. So pesticides and biocides have their own legislation that not only protects, .. aims to protect the environment it also aims to protect the worker. So the person who is actually spraying. Then you can think about somebody mentioned that you're potentially getting exposures through the food we eat. So there are regulations about the residual pesticides that can that are, if you like allowable on your food, and they are monitored by the Food Standards Agency. So that's not what he does. But we work very closely. You can choose to have organic, if you wish, as we all know, it's more expensive. And that's another debate. Then there's a whole raft of legislation that looks at Pollution Prevention and Control. So it's not only putting the onus on industry, it's also putting the onus on the Environment Agency and ourselves to look at how pollution is prevented. And if it can't be prevented, how is it controlled? And how do we reduce Population exposures. Then cosmetics toys, they will come under consumer products. We have consumer product legislation that limits the quantity and the amount of potential pollutants in cosmetics or in toys or in any other consumer product. And that is that piece of legislation is led by BEIS, which is the Department for business, energy, Innovation and Skills. I think we'll get that right. And then of course, we have a whole raft of environmental protection legislation which is led by the Environment Agency, but again, we work very closely with them. Somebody mentioned the the B word Brexit and what will happen with regard to legislation. When we leave Europe and how we leave Europe I think is the imprtant point. My heaart is with.. my head is saying, we need to have an i can't, shouldn't comment.

**Facilitator 3** 28:09

If you've got time later, maybe have a discussion on that. But it's a very important issue.

**Facilitator 3** 28:15

To be honest, the UK has a very good reputation when it comes to environmental protection and population protection. That doesn't mean we can't do better. But it has a good reputation compared to some European countries, other European countries are much more conservative than we are. We're seen as a as a balance, if you like to talk about that, as well. One of the ways we protect and if you like, give information to either users or manufacturers is that you've probably seen product labels. They're not always intuitive. For example, the one that's labelled one that's for serious health hazard things like carcinogens But it's not necessarily intuitive. then number three is hazardous to the environment. So you probably start to look at products you pick up and start to have a look what what labels are on there. gases under pressure, that's more of like a physical hazard. So you have to be cautious about that. And then things that may be fatal to health, that classic poison sign.

**Male FC Member** 29:24

The bit about the serious health hazard.

**Male FC Member** 29:27

I think the government has a problem with getting the necessary information out to the public. Yeah, I mean, it's only it's only very, very recently. I found out that new cars are more polluting in different ways, than old cars, mainly from the brakes and the tires. And if I hadn't chosen to look at whatever programme it was on the telly I wouldn't know that. But it's been known about for quite a while. So somewhere along the line part of the link between knowing about the information and getting it over to the public is either not there or not working very well.

**Facilitator 3** 30:19

I have to agree with you in some extent, yes, it is difficult to get all of the information to all of the population that yourself you seem to be quite interested. Therefore, you're going out and looking for that information. But we mentioned about the train tickets. It's a similar thing. You know, people don't come and give you that information. You have to go and look for it. That is a an issue.

**Lady FC Member** 30:44

Yeah. I agree. I think it is an issue it it's to do with a lot of information. Unless you're very savvy at home, search on the internet, which I am. You're not going to know unless that I think there's doesn't seem to be any duty for manufacturers to actually disclose that sort of information so that if you make it known that the information is there, if you don't find it, I think that that's it's just a chain of Yeah, where that responsibility Yeah,

**Male FC Member** 31:21

it could be so hard to find you can go on to the right site. And it can be so difficult to find what you actually want. I mean, I have very little patience on the computer. Again, because of this. And I'll get a get on the computer and I think I've got to look at so and so and so and so. And I'll spend quarter around 20 minutes trying to find something and it's so difficult to find the exact thing what... I give up.

**Facilitator 3** 31:56

But just to come back to your points manufacturers are legally bound to have that information, how easy it is to find your right is not always that easy, but for many things they are duty bound to do.

**Male FC member 2** 32:13

Manufacturers dont feel they have a real responsibility to make this information available, or more easily available and most say it is on the website.

**Male FC Member** 32:28

Most of them want to release the minimum amount possible at any one time

**Male FC member 2** 32:35

If, if the laws, say state, you have to provide this information. For example, is the traffic light system will have. That’s something quite simple for helping everybody. I look at the stuff, and I think, this si all green and partially red, ok find. How long is that taken to be on our food especially foods with fats. The manufacturers don't take the moral responsibility, to say this is not allowed. And I would do everything everything to get to green, before actually implement the law, thats the way I see it. them myself

**Lady FC Member** 33:41

Can i ask you to clarify ... is it regulatory or is it legal because this is a different that is an that that that possibly is, what the problem is. That people aren't obliged, or they won't be penalised. They're just advised.

**Facilitator 3** 34:02

I think as I've tried to say earlier, there are lots of bits of regulation. There's lots of bits of legislation. So, it depends as you say, on where, where the chemical fits, and what they are actually obliged to do. There are, so for example, if you have a breach of the limits of pollution in drinking water, your water company can be fined and would be fined. So there is there are disincentives out there. But of course, it's it's the monitoring side of things that how often do you have your water monitored? And you know, and actually understand whether you have got an issue or not. So, yes, totally take your points. I don't think we're in an ideal situation. And maybe it's for us to think us as citizens. How can that be done better. In some countries, you can find information more easily, but not necessarily all the information. So, yeah, it's it's a minefield, perhaps in many ways, but with the labelling that I was trying to say, a company who produces any product that has a carcinogen in it is legally bound to have this sign put on it. And if they didn't, there would be huge repercussions to the company for that.

**Facilitator 3** 35:40

Same with things that hazardous to the environment. We can come back to that.

**Facilitator 3** 35:48

So then I just had to just just again, to give you a bit of a feel of what else is out there. You've probably heard about the regulations that deal with recycling. So The top picture there is computers and electronics. There are regulations that deal with how we recycle electronics, which is a relatively new piece of legislation because we're in an era now where, you know, I've got two phones and a laptop and I've got Kindle at home. And what do I do with all of that when it's not working anymore? As you can imagine it, that's a big issue. I think when we were having our pre discussions, people talked about plastics. So that's a picture there of plastic pollution. I don't remember where I got the picture from, but you can often see that in, in rivers and, and I think somebody also mentioned it's also to do with our choices, the way what we do, it's not only at manufacturers, how we deal with things. There's a sewage plant in there. So in the UK, we can drink water from our taps. We know it's good for you. It's healthy, mostly. In other countries, you can't do that. Even in France, I wouldn't drink the water from a tap in, certainly not in Paris, because we know that the systems aren't as good. I talked about cosmetics already. And then of course, there are chemicals in pharmaceuticals. And they are very well regulated. We think about cleaning products. And they if you go home now and have a look at the labels on your cleaning products, it will give you all those hazard warning signs I spoke about. And then of course, there's a whole range of chemicals in consumer products. And we have a consumer product legislation that's at present, it's European, and of course, when if we leave Europe, it will be transferred into UK legislation. And there's more. I could talk for hours on the kinds of legislation that's out there. But as the discussion has gone, you can see that there are also areas where we can do better. This is really part of the discussion. So what do we do in Public Health England, we do what's called a risk assessment. We try and understand what the hazard is. So that's a chemical what is its hazard and understanding it's how much we might be exposed to, we then carry out a risk assessment. So to give you an example from from a domestic situation, if I go back one, you can see the other the cleaning products, you can imagine that the strength of the chemical in your toilet cleaner. Just when you use it, you probably realise that it is a very powerful chemical. The hazard is the potential that is the property of that chemical to cause ill health. So if you drop some of the toilet cleaner on your hand, it's a corrosive, you would feel it straight away. Sadly, some people actually drink it by accident or otherwise. And you can imagine that the damage it does Because of its corrosive nature, but what we do is we put it into a bottle with a hopefully a child friendly lid. And that's how we are reducing the risk, because we're trying to stop the exposure. So that's what we do on a day to day basis at work is if you get chemicals in different media. And we look at what are the health effects? Are they serious health effects? Are they more mild health effects?

**Facilitator 3** 39:28

Are we as a population exposed to those chemicals and then we do the risk assessment. That's what we do in a nutshell that that's my job description you see on one slide.

**Facilitator 3** 39:42

Now, over to you. This si where we would like you to start to think about a number of questions. So, I'll give you a couple of minutes to think I'll show you the questions. Give me a couple of minutes just to have a thing. And then we as a group will talk to you.

**Facilitator 3** 39:59

So the first question is what are your perceptions of chemicals in the environment? And I will leave the other questions up. So you don't need to write the questions but think about what your answers are.

**Facilitator 3** 40:12

What chemicals are you concerned about? And if possible, why.

**Facilitator 3** 40:19

Do you feel, Ive given you a really quick Whistle Stop basic description of regulations and policies, but from your perception, do you feel they are sufficient to protect us? So that's the legislation for chemicals in air, water, food, cosmetics?

**Facilitator 3** 40:38

And finally, what information would you need or want to answer those questions? If you really can't answer those questions, what information do you as a citizen really want? So I will give you a couple of minutes to thing about that and then we'll we'll get together.

**Facilitator 3** 41:23

Fascilitoatro plays music ....

**Male member 4** 42:11

My perceptions of the general subject so largely governed by what I've seen on television heard on the radio, read in magazines and newspapers. For example, David Attenborough's documentary about ocean plastic made me aware of the issue in a way I think that I never was before. And then one reason that paper and so on about the exhaust fumes from diesel engines and stuff like that, so, awareness is growing, the natural tendency of bureaucrats is to rush in and manifest a whole load of regulation to stop it. Often it's a blunt instrument, when something much more focused and targeted, and well thought through would do a far better job. And I think the official mindset runs to this thought. But having put the regulations in progress, and if we can create legislation around the core of those regulations, anyone breaking the regulations is de facto breaking the law. And then we make Thames water pay for polluting the reservoirs as they did recently.

**Male member 4** 43:40

So I think there clearly is a case for both regulation and legislation in this field. But I think there's a risk that we create too many regulations. Of an imprecise nature. That are not properly thought through.

**Male member 4** 44:03

And it's politicians and bureaucrats saying, 'Well, we've done something'. You the public told us that you're worried about this stuff that we've done something. We've passed an act that makes it illegal.

**Male member 4** 44:20

The the issue about communication in my mind runs along fairly similar lines, which is that I and others might argue that government talks to us far too much already.

**Male member 4** 44:39

And they often don't talk to us about things that matter to us, but rather about things that matter to them. This isn't a party political point. This is a comment on politics in general and not just in the UK either. Hm! So, if we want to change that one of the things we have to do is to convince them that the first of all the basis from which they get their information, organisations like yours is focused on the right kind of communication needs to be made.

**Male member 4** 45:21

So that your job is to explain to politicians, what ought to be communicated, and what can be communicated. It's up to them and their Spin Doctors to figure out how to do it. And you will get feedback from the public in all sorts of ways on these issues.

**Male member 4** 45:41

And like many others, I'm tired of being told you didn't know what you were doing or what you were voting for three and a half years ago, when you voted for or against Brexit. I think most people knew exactly what they were voting for. Whether they They voted to remain or to leave, there was a tonne of information available, if people could be bothered to look. And the reason many of them didn't is because of a lack of awareness.

**Male member 4** 46:14

And that brings me back to the end of the loop here, which is that the public, I think, are becoming more and more aware of pollution, and heavy chemicals in water and food and plastic in the ocean, and all of these kinds of things.

**Male member 4** 46:36

And what we need, I believe, is a guide from government or from executive agencies, or maybe both about a broad ballpark area where we can look for more information on these topics, and perhaps specific websites, and where we can find answers to questions. So I think the objective communication in this realm is to make the public more aware. And more aware in a more focused, targeted way than government is generally very good at so that we can begin to ask the right questions of people like you (PHE scientists).

**Male Member 3** 47:29

Could I just say something about that. It was also to do with the quality of information, wasn't it? Yeah, if I use BREXIT as an example. Yeah, there's a lot of the information that was brought out was a bad quality and I say a lot of the information about chemicals seems to me to be the same, especially in the likes of newspapers.

**Male Member 3** 47:50

One thing I'm really quite angry about is the way things are policed and the example that makes me angry mad I think is Volkswagen with their criminal activity of doctoring the figures on particulates that their cars are producing.

**Male member 4** 48:10

And not just them,

**Male Member 3** 48:11

Not just them. That's right. And getting away with it. The people who were in charge who you could probably calculate how many lives have been lost something like that with-in confidence limits, but they got away with it. And I think for me, you know, that makes me think, well, regulations are okay. But if people can just get away with breaking them.

**Male FC Member** 48:43

They've already paid 49 billion in fines in court fines, and they do they've just been they've been taken to court in Germany now. They're expecting another 37 billion from that.

**Male Member 3** 48:58

Yeah, that to me, that sort eccos the tobacco industry and the fines and the court cases and things like that. Which, yes, to some level of punishment, but to me, not enough of the individuals who were involved to make these decisions.

**Facilitator 1** 49:18

So am I hearing at least from first two gentlemen that you feel that there aren sufficient regulations? It's more to do with the policing of them.

**Male member 4** 49:28

No, no, I think there are too many regulations in some fields, not necessarily around pollution. And those regulations are a form of government bombardment. We bombard the public and companies and organisations with all these regulations, no one has time to read them. And nobody even knows what they are in many cases.

**Male member 4** 49:54

And I've almost finished. So my argument is that the regulations need to be there. But they need to be much more precise, much more focused on the kinds of things that [name] was just talking about.

**Male FC Member** 50:10

I think a big problem is, when you look at Westminster when you look at all the people involved, there's thousands of people looking at all these various things. And at the end of the day, all these various things that those thousands of people are looking at, go to the individual.

**Male FC Member** 50:30

So the individual has got no chance of making sense of all that stuff that has come from thousands of people, there's too much okay. You just can't cope with it all.

**Male FC member 2** 50:47

What I was going to say is that, with the amount of information you're saying there's too much regulation. Every law or regulation is needed at this created, somebody will justify it.

**Male FC member 2** 51:09

Whereas you'd say this to too much that somebody will come up, come along with a counter arguement and say this is why it's needed. Already, as I said earlier, at exit time from Europe. People are looking at reducing regulation. But I know when I was working some of the regulation which came in came through Europe and that there to protect us. Now from my mind, you said to protect us? Is it too much? First question.

**Male FC member 2** 51:52

Sorry. There's too much. There's already somebody who say Oh, But if we get you out of Europe we can get rid of this red tape. So companies can do what they like, big business can do what they like without people stopping them, or someone saying you cant do that or somebody taking you to court sometime.

**Male FC member 2** 52:24

And often it's very much a balance. I think it is easy on one side to say yes red tape there too much red tape. But the consumer asks what is the red tape there for. There's always somebody will say hard so by giving you this, then somebody else just got there because.....

**Facilitator 1** 52:43

QUESTION 2 Okay, so that's the bit on the regulation so what chemicals are you concerned about? From an environmental perspective?

**Male FC member 2** 52:50

For me its, basically its general. Whats in the air? You breathe something one day, like pesticides, you know, allergens. Cars for instance, the company at themoment selling diesel. Years ago, they certainly said get diesel (car). What does diesel do in the environment. So they.....

**Lady FC Member** 53:34

I was gona talk about chemicals. The concerned about. One of the one of the things that I think goes on in research that you do is you look at single chemicals. And one of the things that I think perhaps isn't looked at enough is the cocktail of chemicals that we're absorbing. Because we seem to have a lot more stuff that's going on. And the, it's actually how, how, what, what can be done about that, for example,

**Lady FC Member** 54:16

I just wrote down some examples that was factory farmed animals are routinely fed antibiotics as a prophylactic, rather than actually to treat condition and those antibiotics, presumably and I'm saying presumably because of, I don't know, got access to research, but they're in the meat and therefore when we eat that meat we're taking in antibiotics. Now we know there's a crisis with antibiotics. in so much as, you're getting super bugs which got much thicker proteins shells, which don't respond to the antibiotics that we currently take. And it's that sort of thing, which seems to me to be like a pandemic situation, that it's not really being addressed. For example, meat, if it's not organic, doesn't say might contain antibiotic. So if there's research that will actually show that that's the case. So that that's sort of something that bothers me as a member of the public,

**Facilitator 1** 55:27

So the Food Standards Agency would monitor the amount of antibiotics in meat again, that's not my area of expertise. So I don't know, again, they can't look at every single carcus that is slaugthered.

**Lady FC Member** 55:38

...... but it's actually to do with I think part of what we're talking about is some measuring, later on in the discussion. But it seems to me if there is a sort of .....roles that you have, there are these crosswalks that are going on, and I would say that antibiotics in some ways are a dangerous chemical so much as they're going to invalidate themselves at a future point in time. How they are used.

**Lady FC Member** 56:12

The other concern that I've got hm... concerns pesticides. Currently in the EU, the banned systemic pesticides, and the systemic pesticides actually are absorbed by the plant and stay in the plant, whereas the ones which are regulated through the EU, they could be washed away they're on the surface. And I started looking in the supermarkets at where things come from. For example, if grapes come from Spain, that's that's a washable off. But when you get in things that are coming in, outside Spain for outside the EU, for example, from South America, taking the example of grapes. What? How do we know they're not filled with pesticides?

**Lady FC Member** 57:07

You know, it's actually this? You know, that's a labelling issue. It's, it's how far does the research or the regulatory function cover that, you know, we've got regulations about what we produce, but actually what we're bringing in, is that similarly legislated?

**Facilitator 1** 57:33

Again, not everything can be measured, but if you're importing food or other items, there is legislation to say, you know, the levels of in this case, pesticides that can be found on the right, I'm not saying it's perfect,

**Facilitator 1** 57:47

yeah. But it is. I think, as a concerned member of the public then it's sort of like I don't know that.

**Facilitator 1** 57:54

I'm getting back to the information, the information I'm fairly good at doing my research, but I I just started deciding not to buy things from outside Europe for that reason.

**Lady FC Member** 58:09

So, I guess those are sort of the main current concerns that I've got.

**Facilitator 1** 58:15

Yeah. And I'm sure we'll come back to that. Thank you

**Lady FC member 2** 58:20

Yeah, I'm not I kind of feel completely ignorant and I also feel like maybe I'm a bit too trusting because I assume that I also I have, I have issues with weighing up the benefits to me and the benefits to the environment because for example, I would trust that the water that comes out of my taps is fine. And I always drink tap water and I drink loads and loads of water and I have a go at my family for constantly buying bottled water because of single use plastics and I'm trying my trying to get away from that. Yeah, in my house, but that but now I'm like, What is my water, alright? You saying that it's not? And then and then similarly, things like I'm trying to use, like not use loads of bleach, you know, and like, I've tried to use more natural things to clean with. Because the more not so much that I'm concerned about my health, but more with the environment and things going into the sea and stuff like that. So I don't know, I just I feel like I'm just there's obviously stuff out there. I feel like I have no got a clue, really and I don't have is that I haven't looked at or I'm just, you know, it's just I don't read enough or ....

**Male member 4** 59:30

the lead. Sorry.

**Facilitator 3** 59:33

Maybe just let [name] say what she thinks. Then we'll come back.

**Female Member 2** 59:38

I was listening to everything and I thought came into my mind when a problem arises. And you hear it on the news, oh.

**Female Member 2** 59:52

Legislation has been passed to solve that problem. How far does that legislation go? Are they using it as a plaster to cover up something that is what came into my thoughts it may seem that is what's going on. Because they bring the legislation like legislation in to cover that problem. It wouldn't happen again. But three months down the line it has occurred. So how far did it go? It just was covering a sour that is not going to go away.

**Facilitator 3** 1:00:28

Do you mean things like plastics?

**Female Member 2** 1:00:29

Yeah, that sort of thing.

**Male Member 3** 1:00:30

I think it's covering a politicians backside that is wat it's doing.

**Female Member 2** 1:00:34

Well yeah, I wouldn't .... And isn't is our work with them. And most of the time, they're discovering their own backs. Literally. They're covering their own bass they don't care a damn about the rest of us once they are okay.

**Male member 4** 1:00:59

I must put in a word of defence for politicians the poor sods.....

**Male member 4** 1:01:05

They often know less about these topics than we do as members of the public, even though they're surrounded by advisors, but what they are is under extreme pressure to do something. I better say something publicly on TV, or at a party conference or seminar somewhere, give a speech which is widely reported in the media, or even to pass an act in Parliament to do something. And, and back to my original point, it's all the pressure creates problems of its own. We must have regulation but as [Name] explained a few minutes ago, that didn't stop the German car manufacturers driving a 'Coach and Horses' through all the regulations that existed at the time on diesel emissions. And whether or not it was criminal in Germany, we're about to find out, as you say, but in many cases it is. And in many cases it isn't. I just feel a little bit sorry for politicians who are being pressured into doing something, when no one's quite sure what it is that should be done

**Male Member 3** 1:02:25

Thats what they have committees for.

**Male member 4** 1:02:27

If you take the first point up here, I mean, how can any of us answer this question because we're not expert enough. So in my case, the discussions I have with my friends and my wife were concerned about particulates of plastic in drinking water and the oceans generally, we're concerned about heavy metals like lead coming out of diesel engines and smokestacks we're concerned about toxins and dioxins. We're concerned about, but but do I know? And do my friends know what any of these things actually mean? in detail? Not really, we're repeating cliches that we've heard on the television. So we need a lead from. To answer this question, I believe we need to lead from medical practitioners, people with medically trained who can say there's a whole load of chemicals in the air in the soil, we eat them, breath them, drink them, many of them are not dangerous, but some are and here's a list of them. And here's a list of the things to government. I mean, here's a list of the things that are dangerous, and where they can be found in what food products cosmetics and so on, and which groups are most at risk from these chemicals, whatever they are. Only then I think should government say, hang on, we have a point to make here to you, the general public, and it is this and give us some guidance rather than more and more regulations.

**Lady FC Member** 1:04:16

Does something like like not already exist?

**Facilitator 3** 1:04:18

It does, you know, the what the issue. Again, personally not talking as PHE, one of the issues I can see is that most of you are very level headed, but then you get other members of the public who say, No, no, we've got to do this. We've got to do that. And you're right, then the politicians have to do something. So, it's a balance, ......

**Male Member 3** 1:04:42

Yeah, I'm sorry, I did make the list. But, as far as I'm concerned, personally, I'm happy with the water I drink. If there's anything that affects my health, it's probably the unhealthy food the eat, the biscuits. That's all consuming this mini interview. Things like that. But I think what concerns me most is vulnerable groups, so workers and things like asbestos, which seems quite old fashioned now, but we still thousands of people dying from asbestosis every year. And I'm concerned about how the compensation rules seem to change as well in how much you can prove who is subjected you to asbestos and things that affect children. So I've got endocrine disruptors, things that are gonna muck about with your hormones and the effects of that. And I've got these aerosols and particulates, whatever you want to call them. And I guess that includes hm plastics is now now micro particles of plastics. I think going on from what you were saying, We need someone to say, you know, here's a list of bad things. And here's a list of the things we should we should do things about. But I know I did some work on PCBs, polychlorinated biphenyls, which were thought to be wonder chemicals and buried in that and not going to damage anything. And then I made it a point to try and learn a lot about them when I was researching where they were in the environment. And it was still a matter of opinion as to how much harm they were causing, that you were saying about when you come to other chemicals, what, what harm they're causing. And maybe we shouldn't be looking for these straightforward answers. We should be thinking, well, how much do we personally consider the precautionary principle to be important? You know, how much do we say, well, we're gonna take this risk or not, but there aren't. Unfortunately, there aren't any straightforward answer. There are somethings, isn't there, smoking is bad for you give it up. Yeah. But a lot of it is a matter of degree isn't it!

**Male member 4** 1:07:17

Can I offer one other thought? I'm moving on to a slightly different topic really, which is what to do about all this. The clearly people in this room if we are representative of the general public, don't worry about this stuff or concern might be a better term. And there's a concept in economics called external externalities, which is the management of costs in a chain of events. And at the moment, I walk around London as we all do, or wherever we live.

**Male member 4** 1:07:55

And I see one of the visible externalities which is polystyrene food packaging lying on the pavement, plastic bottles like that one. And beer cans and all sorts of other things. These are visible external externalities, the manufacturer of the cans or the retailer of them has passed on the externality to the general public. Because the local authority where we live has to employ someone to go around the streets and pick this stuff up. Otherwise, we'd be waiste deep in it by next weekend. I mean, it's that bad I think.

**Male member 4** 1:08:40

Now, this may not be dangerous to public health, but it's a visible and easy to explain, easy to grasp, easy to understand concept of how externalities work. Now, how do you stop this? How do you stop litter? In the UK Well, the answer quite clearly I think, is not for the government to spend millions hiring Richard Branson or somebody else to be the public face of the 'Litter Tsar' and encourage us all to stop doing what we're doing.

**Male member 4** 1:09:17

The answer is to force the externality back to who created it and pass the cost back to them. Believe me if this were done, and I've seen it done in business in other areas, lots of times, it would stop within months. The litter problem in the UK would stop within months.

**Female Member 2** 1:09:42

I dont understand what you mean, how does that work?

**Male member 4** 1:09:44

Okay, how it works is that legislation is needed to force the producer of the beer can or the polystyrene or this Tetra pack here to pay for it's just dispousal, okay? Instead of the taxpayer paying for it in the form of refuse collection on the street,

**Male FC Member** 1:10:11

You know that that way you're not punishing the person who's responsible for it?

**Female Member 2** 1:10:16

No, you're not, you're saying that someone has to pay. And at the moment the general public have to pay

**Male FC Member** 1:10:23

The general public cause the problem. It's not the manufacturer that causes is

**Male member 4** 1:10:30

The general public who cause the problem, but unless you fine everybody who drops one in the street, 80 quid, for which we would need 27 million.

**Male Member 3** 1:10:41

They do that now anyway,

**Facilitator 1** 1:10:42

But maybe we should expand that not to dropping it in the streets. But how do you recycle it?

**Female Member 2** 1:10:47

Yeah, yeah, maybe.

**Male FC Member** 1:10:50

You should, an easy way around it would be to put the tax on the product that the consumer pays for and that Tax goes back to the manufacturer for them to respond being responsible for disposing of it.

**Male member 4** 1:11:07

Some of us in this room are old enough to remember as a child when we collected Corona ball or any bottles Yeah, and took them back to shore

**Female Member 2** 1:11:23

Money for bottles

**Facilitator 3** 1:11:27

So I need to so bring this back. I totally agree. I think recycling is another another big issue and that is a is also a chemical issue. It does go back to who pays? What do we do? Do we do we do tax to the companies or do we tax us as citizens, big debates and there are new legislations coming in sadly to say about recycling as well.

**Lady FC Member** 1:11:56

Just don't go to the supermarket. I've stopped going so much to [Supermarket name]. They send me vouchers to give me discounts on buying my fruit and veg from an organic.. Behavioural change. It's a behavioral change.

**Male FC Member** 1:12:09

I think that the parents, the onus, the onus, a lot of theonus ha has to be on parents to teach their children what they're doing besides throw a can away yes, yes. I mean,...

**Facilitator 3** 1:12:22

Lets take short break. Thats is very true for many things. I blame the parents and I am parent. So should we have a short break for 5 -10 minutes, and then we'll move to the next session, which is about the project that [name] and I our working on. Brilliant. Thank you very much. Yeah,

**Facilitator 3** 1:12:52

BREAK -

**Chemicals FG 1.10.19. Part 2 V2**

Thu, 8/13 4:29PM • 59:48

**SUMMARY KEYWORDS**

people, chemicals, samples, project, results, programme, countries, gp, uk, population, exposure, levels, called, lead, information, data, doctor, question, study, absolutely

**SPEAKERS**

Facilitator 3, Male FC Member, Facilitator 2, Facilitator 1, Lady FC Member, Lady FC member 2, Male Member 3, Male FC member 2, Male member 4, Presentation

**Presentation** 00:00

Then hours after closure, like Bisphenol A, excretion occurs mainly through urine, also through faeces and to a lesser extent through breath or through sweat. To sum up the type and severity of health effects depend on how much of the chemical enters the body, the frequency and duration of exposure, the target organs and tissues reached and the length of time that the chemical remains in the human body. The measurement of chemicals and human body fluids such as blood urine sweat and tissues is called human bio monitoring. It is also possible to detect substances and human body fluids that indicate effects that impact on health. The human bio monitoring initiative for Europe hvm for EU coordinates and advances human bio monitoring in Europe by generating data on the concentration of chemicals in the human body people living in Europe and by investigating possible health effects HBM for EU is for you working to improve your health.

**Facilitator 1** 01:13

So there you are. So that was just a little clip that was produced by the project that we're working on to try and introduce what biomonitoring is. One of the issues we have in the UK is that we don't have a national biomonitoring programme. Many other European countries in the US, China, not China sorry Japan, Korea, have national biomonitoring programmes. What we do more often than not, is that we take environmental samples so we could take water samples, soil samples air samples to understand what's in those media. But we don't always know what we as the population are actually exposed to and that's what biomonitoring would give you.

**Male FC member 2** 02:07

Don't get any of that when people go to hospital for any reason to have a blood test. There's loads and loads of things on the sheet that people search for the I haven't got a clue what they are, don't you get any information from that.

**Facilitator 1** 02:23

Most of the things that your GP would ask to be measured in your blood or urine samples would be measures of, for example, liver function or kidney function.

**Facilitator 1** 02:39

Your GP wouldn't necessarily ask for chemicals to be monitored. Having said that, for example, you can have blood lead measurements done. So that's looking at lead, we'll come back to lead in a little bit. So generally speaking, the GP wouldn't ask for chemicals to be assessed. He or she would ask for effects to be assessed that he can then say, well, your cholesterol is high, you need this medication, your liver function is low, high, you need this medication. So it's if you like, it's one step down the line from a chemical exposure.

**Male FC Member** 03:12

It's just the way things are going with all these various worries about chemicals, plastics, whatever. I think it's going to be the next step in this country anyway to do something like that. Because it's it's sensible,

**Facilitator 1** 03:28

brilliant, healthy Britain.

**Male member 4** 03:29

It costs money though

**Facilitator 1** 03:31

We'll come back to that. You're absolutely right. It costs money. So, again, not wanting to stifle discussion, but I will ramble through this and then we'll come back to the questions

**Facilitator 1** 03:42

When we're talking about HBM. And biomonitoring that in that little clip, it talked about the samples you take. So you know, if you go to your GP and say I want to be I want to know what I've been exposed to. He or she could take certain samples. What kind of samples do you think he could take asked for?

**Facilitator 1** 03:58

What do you normally when you go to GP what samples do give?

**Lady FC Member** 04:03

Urine and blood

**Facilitator 1** 04:06

So you could have your blood taken. Okay, good. You could have a urine sample taken.

**Facilitator 1** 04:14

Nobody's going ..errr!! [Showing a prop] ...it is Tea. What else something such as ...., y

**Lady FC Member** 04:22

sputum

**Facilitator 1** 04:23

you can have sputum samples taken. Absolutely. Well, what else do you think?

**Male FC member 2** 04:26

Feaces checking for cancer.

**Facilitator 1** 04:30

Yes, you could again, that's for. Absolutely right. But that's a, if you like health endpoint rather than exposure. I've been doing by monitoring for many years. And I'm very happy to say I've never had to take a feaces sample.

**Facilitator 1** 04:45

Many, many urine samples.

**Facilitator 1** 04:48

What else?

**Male FC Member** 04:50

skin scrapes

**Facilitator 1** 04:52

Yes, you can take skin samples, right. And it's very simple to just like a piece of sellotape to take a sample of the top layer of skin,

**Male Member 3** 05:01

Sampling dead people,

**Facilitator 1** 05:03

yes, again, and again Im really glad I have not had to do that, that's on the forensic side. From a public health perspective, if the poor person's already dead, I have failed.

**Male Member 3** 05:15

But you can do that for things like dioxins.

**Facilitator 1** 05:17

you can, and we do look at stored samples, so not necessarily from a dead person, but from biologically stored biobanks we do

**Male FC Member** 05:27

hair?

**Facilitator 1** 05:28

Many chemicals in hair. Now you might have heard about that from sports, where you can look for drugs in hair, but you can also look for many other chemicals

**Male FC Member** 05:38

..like DNA swabs,

**Facilitator 1** 05:40

you can absolutely take a swab from inside your, your mouth to get the cells and therefore look at the DNA.

**Male FC Member** 05:48

Do they do fat samples from people?

**Facilitator 1** 05:50

You can I know that there was a study in I think it was in Oxford where they were looking at fat samples. There are two ways you can do it. One is to actually take a little plug of fat usually from person's bottom.

**Facilitator 1** 06:06

Not Not everybody wants to do that I can totally understand why but you can actually take, or get a fat sample from your blood. You can spin down the blood and take fat and DNA actually also from a blood sample. So I asked my husband if he had any....nails.

**Lady FC Member** 06:23

screws

**Lady FC Member** 06:26

nails.

**Facilitator 1** 06:28

You could also look at nail clippings. And these are the main tissues we use for environmental chemical exposures. So blood samples or urine samples, blood and urine are the most used and you can imagine taking a blood sample from a child we try very hard not to do that. You can in some cases take a finger prick sample rather than having to take a whole tube of blood. And then we will take that sample and do an analysis on it. [refering to slides] This is what's called the GCMS

**Facilitator 1** 07:00

So it's basically a piece of kit that you can take part of the sample, put it through and you can, you can chemically analyse what's in that sample. And that's what we do. So we do have these facilities where [name] and I work.

**Facilitator 1** 07:15

Okay, so what I thought I would do now is give you one or two examples of what we have done in the UK. And these are historical examples, and then we'll talk a little bit more about the project that we're involved in. Now, this was already mentioned, [showing a slide of lead in paint etc] what am I referring to?

**Facilitator 1** 07:34

Anybody know.

**Lady FC Member** 07:35

lead

**Facilitator 1** 07:35

yes, absolutely. So we know the lead has been removed from petrol since the 1970s or 1990s, depending on where you are. It used to be used in cosmetics used to be used in paints, it's again there is legislation to it bans lead in paint that will be used by the public or in any kind of product that would then a child may use as a toy,

**Facilitator 1** 08:05

Lead in water is very well regulated and we cna talk about this later if you wish. It is very well regulated in public water supplies. So where you've got Thames water,[example given as the FC was in London] for example or any other water company. If you've got private water supply, then that's another debate to be had.

**Facilitator 1** 08:25

The reason I mentioned lead now so glad [name] mentioned it earlier is because it's a classic example where human biomonitoring has been used to understand whether legislation has worked or not. So when lead in petrol was banned. Many countries banned lead in petrol at different times. And they took biological samples that took blood samples at that time, and they took subsequent samples from the population. So not from individuals to understand are, are the exposures actually dropping. And these are data from the UK. Lots of different pieces of data unfortunately, we didn't have a long standing assessment. And you can see our, [refering to a slide] our data stops at about 1995. We don't have population based data after that. But you can see the trend is a clear drop from when .....when lead was taken out of petrol. And you can see the levels have dropped. And this picture is reproduced in all of the countries where they've also removed lead from petrol. So that to my mind, is to show how, biomonitoring in this case is really shown that legislation has worked from an exposure perspective.

**Facilitator 1** 09:40

So that's a but the question now is, what are our levels now, we don't have that information in the UK population we do ...

**Male FC Member** 09:48

...and also authorised to use input from instead of lead

**Facilitator 1** 09:53

exactly so what was used in one of the things that was used instead of lead was benzene. So you could then look at the levels of benzene or its metabolites and see that the levels have gone up. But again, it is regulated. But that definitely the exposure would have gone up to an extent.

**Facilitator 1** 10:13

But that's a very good point. I'm so glad you metioned that. Because when you remove one chemical, you have to think about what goes in instead. So substitution of chemicals is another big issue for us in public health.

**Facilitator 1** 10:27

Mercury is already been mentioned. Sorry.

**Male FC Member** 10:30

This...Is the sort of information used for pinpointing hotspots around the country as well.

**Facilitator 1** 10:36

Yes. Absolutely. So nowadays, at least for lead as an example, we don't do a population based screening, because we know the levels are quite low on a population basis, but there may be hotspots where we are more likely to do so focusing your efforts in an area where you think there's a problem rather than just doing a blanket screen. Right so very quickly from the previous project.

**Facilitator 1** 10:59

It was the precursor to the project I'm going to mention where we will we use in the UK we're involved with 17, I think other European countries, and we collected hair and urine samples and looked for certain chemicals. We look for Mercury, for example. And this just gives you a snapshot of the results we found [refering to slide]. So the, the type of mercury we're looking for is the mercury you'd find in fish. So it's from food consumption. And you can see where the UK is. This is just a depiction of the the average and then the countries that are above average and the countries that are below average and

**Male FC member 2** 11:34

it's extreme isnt it?

**Facilitator 1** 11:36

It is pretty extreme. It's not that the results are not surprising in the sense that the Spanish and the Portuguese and then the Cypriots going down, eat a lot more fish. They also know the type of fish that are potentially exposed of a potentially contaminated with mercury. But the issue you must also keep in mind, although you can see a huge difference between the UK and Spain, the levels we found, were not at levels where you would be concerned for the general population. You would be concerned if that was a child or a lady of reproductive age because mercury affects the unborn foetus so somebody mentioned susceptible groups. There it's, it's okay for the general population, but you wouldn't want certain susceptible groups to have that kind of level of exposure.

**Male FC Member** 12:30

[name] was mentioning about things in combination as well. Mercury with other exposures seems to have more of an effect. How can this be monitored? What do we do about mixtures?

**Facilitator 1** 12:38

Yes. So the classic example is radon and smoking. We know that radon and smoking and if you're a smoker, you have a certain risk (Addition by transcriber: of cancer). With radon you have a certain risk. If you're living in an area with radon and you smoke your risk level goes significantly higher. I don't know the exact numbers, but it goes incredibly high. But it's a big challenge for us mixtures is a big challenge. What do we do about mixtures? Okay, last example, phthalates you may have heard about them. They're a group of chemicals a very large group of chemicals that are used as plasticizers and preservatives in cosmetics and consumer products. And we looked at some of the metabolites in that previous project I talked about, and you can see again, the blue mark, it doesn't quite see it, but that's where the UK is, in comparison to other countries.

**Facilitator 1** 13:28

So this shows you the sorts of things you can do with biomonitoring data. And then maybe we then want to say, well, in Slovakia, they might need to do something about the levels of phthalates in their consumer products, (for example).

**Facilitator 1** 13:42

Okay, so those are the examples. What are we doing now? Those are, say our historical examples. What are we doing now? So, [name] and I, and other colleagues, we, as you know, work in Public Health England, believe it or not, we're actually working with other government departments. We're working with the Food Standards Agency with Defra, which is a department for Environment, Food and Rural Affairs. We work with the Environment Agency, and the Health and Safety Executive on this European project, which aims to look at population exposures to chemicals be it from food, be it from the environment. And we're also looking at some occupational exposures its a big European project. And I think one of the successes from that project for me personally, is the fact that we have a government wide Steering Group, a UK government wide steering group.

**Facilitator 1** 14:36

It's a five year project we're halfway through, it'll end in December 2021, there are 28 countries not only European it's also includes Israel, Switzerland, Norway, and of course, depending on what happens on Halloween (2019 ). We'll see where we are with regard to this project. It's a cooperative project in the sence that it really is bringing in scientists and policymakers from all of those countries. And what we're hoping to do is to improve environmental health and public health in Europe. And there's a website there [ reference to website]. Obviously, I can give you that. So if you want to look up more about the project, there's a lot of information, newsletters etc... I wanted to give you a feel of what we're trying to do. So from those 27, European that shouldn't say European from those 27 countries.

**Facilitator 1** 15:29

[referring to slides] we are doing what is called an alignment of studies, so we have taken studies from each of those countries and tried to bring together to try to harmonize the protocols that are used and to analyse the same chemicals across all those countries and compare the data. [referring to slide with a summary of sampling frame] In order to get a geographical spread we have divided Europe into four areas, and these areas are actually agreed by the UN. They also use them when they're doing their assessments. And we've got a certain number of sampling groups in each of those areas. We're looking at children aged six to 11, teenagers which are classified as from 12 to 18. Sure, 18 year olds would think they were still teenagers, but for this purpose they are and then we're looking at younger adults. And the reason the main reason for that was to try and capture women of childbearing age so that we're having a proxy for for potential foetal exposure and cost. We could if we wanted to also look at an old age group, but the cost was inhibitive. Maybe in the next project, we can do that as well. So we've looked if you like, and the more vulnerable groups. These are just the acronyms for the countries that will then be collecting samples in those areas. In Well, in the northwest, etc, and the different age groups, and I'm sure you're looking for UK in that list. I'll come back to that in a second. The the ones in red are the countries that have a population based project, whereas the others have either hotspots or more regional. So it's a compromise. It's a compromise. Yes.

**Male FC Member** 17:27

21% plus 41 is 62. doesn't add up to 100. [referring to data on slide]

**Male FC member 2** 17:40

Yes, because each one is just to the nearest to see everything.

**Facilitator 1** 17:45

Yeah, it was not accurate. So and I didn't say that's a as the approximate percentage of population in those areas.

**Male FC Member** 17:58

So how did I How did I make it representative of the whole population.

**Facilitator 1** 18:02

So for example, in Germany, and they've had a population representative sample for many, many years, so what they do is they take the whole population, obviously on paper, and they would look and they do a random selection of regions, then they will do a random selection of areas within those regions. And then a random selection of people in those areas until they get the the number of people they need for their centres.

**Male FC member 2** 18:27

I find this a high take up right.

**Facilitator 1** 18:31

In Germany, yes, at present, because they've been doing it for a number of years, and people are used to it. So I mentioned here now in the UK and present, we haven't got a nationally representative study, except for the health survey for England over whether you've heard about that. But that is only in England. They don't do chemical analysis. That's what we're trying to incorporate into the health service in England. They look only at health endpoints, those sorts of things that you mentioned in Nothing's like your cholesterol. And when you go to a doctor, what what does he ask you for cholesterol levels? kidney function, liver function?

**Male member 4** 19:09

What can we learn from a driven study that's been running for a number of years? So have we access to their data?

**Facilitator 1** 19:16

Yes, we do. In fact, their data is, is almost all of it is available on the internet, if you wish to look, the Germans by coincidence, also coordinating this project. So we are learning a lot from them, not only from our German colleagues, but also from the French, the Belgians who have also had long standing projects. So one of the aims of this project really is to learn from those in RED for those who are then trying to set these things up.

**Male member 4** 19:45

But is it too early to say whether or not there are some key findings from the German study

**Facilitator 1** 19:51

or the Germans, they've got lots of key findings, but it's only focused on the German population.

**Male member 4** 19:58

German population industrialised western country its pretty much aligned with other parts of western Europe.

**Facilitator 1** 20:07

Yes, exactly. And this is why we've split Europe into those areas is not only geographical, in fact, the UK is within the North, is to try and to see whether in certain areas do you do see a similar pattern? Also, if we didn't want to invest in a big study, could we use the German data as a proxy for the UK? We do at present, but we don't know that for sure.

**Male member 4** 20:34

I mean, do we know for example, in Germany, whether people in cities are more or less exposed to these chemicals?

**Facilitator 1** 20:46

I could easily just say, yes, it depends on which chemical but yes, that's the kind of information they have. So for example, with pesticides, they have a good understanding of exposures in rural areas as compared to urban areas. And they also have A lot of information hotspots. So what are we actually looking for? So what we've done is, what you do is not only do you collect samples, these biological samples, but you also administer a questionnaire. We asked them about the things or the things we've asked you. What's your age, obviously, what's your gender. Also want to know where you were born, how long you've been living in the area when the samples were collected, because that is difference. You've asked about education as a proxy for your socio economic status. also asked about the environment in which you live. So it's a very extensive questionnaire in order to get as much information as possible. And this is just a flavour of the sorts of chemicals we're looking at. We're looking at phthalates, which is what I showed in the previous slide is the chemicals that are found in consumer products. And some of them have now been substituted and they've been substituted for a compound called dinch. So we're also looking for the substitute looking at flame retardants. As you might have heard in the in the news, there are many issues with flame retardants and chemicals are called PFASs these are perfluorinated alkyl substances, so they are long chains of carbon with lots of fluorines on and then we have a few issues with that, but also looking at chemicals called bisphenols. You might have heard of Bisphenol A, which was found in most plastics again as a plasticizer. It's now being banned in food contact materials being banned and children's baby feeding bottles etc. Again, as a means to reduce exposure to the vulnerable groups, looking at cadmium polyaromatic hydrocarbons, these are just some of the environmental pollutants that we're looking at to give you a flavour of what we're trying to do. We don't have results as yet because we're halfway through the project we're collecting or our fellows in the other countries are collecting samples as we speak, and then we'll do the analysis and then we'll Be able to produce data to show the sorts of exposure levels for the different countries, the different regions, and will be able to compare and contrast with how legislation has also taken up in these different countries.

**Facilitator 1** 23:17

So, I've spoken a lot. I'm going to take a rest now. And see if you want to have a quick look at those questions, and then we'll go through them together. So the first, although maybe put that on there. I'm sorry. So the first question I'd like you to think about is not on the list.

**Facilitator 1** 23:39

What I'd like to understand is, before you were invited to this focus group, had you heard of biomonitoring before?

**Male FC Member** 23:50

No,

**Facilitator 1** 23:51

nobody Not at all.

**Male FC member 2** 23:52

Yeah.

**Facilitator 1** 23:54

Especially with your background, we talked about lead etc.

**Male FC member 2** 23:57

I try and keep up

**Male FC Member** 24:00

I realise that there were these things out there that I didn't realise what it was called until I actually looked into it.

**Facilitator 1** 24:06

That's so true. Yeah, that's true. I mean, you probably know when you go to the doctor he or she is doing biomonitoring, but that's more for clinical effects, whereas this is what environmental exposures. Good so really only [name] had heard of biomonitoring before being invited.

**Facilitator 1** 24:25

Next question - have you ever taken part in a study that either sort of thing I mentioned before that either looks at chemical exposures or the effect of the environment on your cells?

**Male FC member 2** 24:39

No.

**Facilitator 1** 24:40

No one's taking part in UK Biobank for example.

**Male FC member 2** 24:44

Yeah, yeah.

**Facilitator 1** 24:46

So Biobank, I did too. So we are biobankers. So UK Biobank is a huge study funded by the Wellcome Trust, and it looks at probably had blood samples taken urine samples, your height, your weight, And lots of different questionnaires are similar to what we're trying to do. But they won't be assessing environmental chemical exposures that's the difference but it is it's also not a representatives UK representative study because you're there as a volunteer. I was obviously interested there's no way I would say no and you obviously intersted and have taken part. Anybody else taken part in any other kind of study where there may be looking at environmental exposures

**Lady FC Member** 25:33

When I was diagnosed with pancreatic cysts, Barts asked me to...they had a questionnaire to I think was to do with. Do you lifestyle made lifestyle lifelike,

**Facilitator 1** 25:47

but it didn't take samples to analyse fro chemicals

**Lady FC Member** 25:49

no can it never I never heard any more about it. I thought about the question. We're trying to monitor what triggers whether it's actually environmental or to do with disease, childhood diseases,

**Facilitator 1** 26:04

okay? Very interesting. Okay, so let's think about these questions and we haven't got long, so I'm gonna have to shimmy us through this.

**Facilitator 1** 26:16

So the first question is about if we're at the end of this project in 2022. And we're coming to a conclusion. What do you as a citizen want from the project and I know we've given you lots of information in a very short time, but just to get a feeling for what you would want with regard to let's say results of content and what we're calling impact which is more to do with legislation or regulation.

**Facilitator 1** 27:15

Okay, then the second question in that that little group, ideally we would have had a meeting like this before the project started, and I would have asked you these questions before. But now we're near the end of the project. And you as a citizen, if you were, you know, just a member of the population, not a participant, how would you like the results to be communicated to you?

**Facilitator 1** 27:44

And then if you're an actual participant if you had your blood or urine taken, how would you like your personal results to be communicated with you? Give you a minute, and then let's have a quick discussion. Then we'll move On to the next

**Male FC member 2** 28:01

Is that some something that is within the remit of this project? So are they deciding what to do about this individual results?

**Facilitator 1** 28:09

Yes. And interesting question because it's different in different countries. Because I didn't mention this, I couldn't mention everything was that you have to have ethical approval if you're going to take a sample from anyone. And it could even just be a urine sample, which is non invasive, you have to have ethical approval. And during your ethics application, they will ask you, so how are you going to report the results back? And if you find a result, that's alarming. What will you do about it? Because it would be unethical for me to for example, to have a high blood lead level and tell you nothing about it.

**Male FC member 2** 28:46

Will all the results in the public realm, they would be anonymize?

**Male FC member 2** 28:52

So that wouldn't be an incentive for private companies to be buying the results because they're available to everyone.

**Facilitator 1** 28:58

Absolutely. So the other parts of the project with we've got a website called IPChem, it's a European site, and all the data will be uploaded. And as you say, there'll be anonymous data. So that I mean, researchers then can ask, can we have more detail, but you'd have to apply for that. And I don't know whether you've heard of the new GDPR, which is the general directive for data protection, exactly, which is causing us some issues with what you can and can't then put on on even on anonymous website. So a few thoughts.

**Facilitator 1** 29:46

Let's go to the second question. If you were just a member of the population, what have you like the information we talked a bit about how information should be communicated? What would you think is the optimum way of communicating the results with you, as a member of the public,

**Male FC member 2** 30:04

if biomonitoring was started, and a voluntary thing. Your GP could ask anybody who's having a blood test. The GP could ask them if they'd be prepared to participate in the biomonitoring so that, you know, at the time that's happening, the doctor could give you an information leaflet about what it is and say when you go back again, to get results of your blood test if there's information out there about any results of anything that's been done, it could give you an information leaflet for that

**Facilitator 1** 30:54

well. It should go through you would be more comfortable if it goes through your GP.

**Male FC member 2** 30:57

No. I think people would take more notice if it, you know it people see a programme on the telly, horizon panorama, whatever they are now there's a good film on the other channels that switch over, or they turn the radio off or they're reading a paper and there's some information in there and they just skip that. If it came from your doctor and you'd offered to participate in it, then I think you'd be far more inclined to take some notice of an information leaflet that you're given.

**Facilitator 1** 31:39

So there you're talking as you as as a participant. What about if you were not a participant? Well, as a member of general public, what would you like to know how,

**Male FC Member** 31:48

if you were a memebr of the general public, A. you may not be aware what is going on. Then when the results come out, publish the results. And that should be by general media. because you cant expect everybody to go to their doctor.

**Facilitator 1** 32:11

no, no, absolutely. So what I mean by General media

**Male FC Member** 32:15

newspaper, radio, TV, social media. people are made aware that the project is complete.

**Male FC member 2** 32:24

Oh, that doesn't work. You've only got to you've only got to look at the past. And people don't want to see things like that. They don't want to read about

**Male FC Member** 32:34

they may not want to what say and of course, okay. But there has to be available there. The a choice is yours when we go further information or not? The choice is yours

**Lady FC Member** 32:55

somes time social media ...

**Male FC Member** 32:56

is Yeah, but it has to it has to be there. for the someone to see. but we can't make say right you got read it all or whatever the information is this is a project that is the most if any of

**Facilitator 1** 33:11

If you were a participant, then what would you like

**Male FC Member** 33:13

have participant that's different because you are a participant or something's being found untoward , in my blood or whatever, obviously you got a duty of care to me to say right you've found this problem

**Facilitator 1** 33:30

how would you like the results conveyed to you as a participant?

**Male FC Member** 33:37

often as two choices how to be told them personally or contacting personally or they contact your doctor. But then when you start the project youre saying, you've got to give your doctors detail, at the start of the project. So therefore I'd have thought it would be better that you are advised to see your doctor. We can download or whatever all the way to whether the problem is built see. or is it a case of going to your a doctor can advised to go for a further blood test or futher investigation

**Lady FC Member** 34:19

I think if I was a participant because it always causes anxiety if you get called in by your doctor, I think it would be like the everybody who participated went back to see the doctor to get the results. There's no sort of, a bit like worrying if you got cancer or not because of the negaive test. I think that might take the sting out of it a bit.

**Male FC member 2** 34:50

But do doctors have the skills to analyse to interpret the results

**Lady FC Member** 34:55

I think it would be like -if it was interpreted for the doctor and they convey information

**Facilitator 1** 35:09

You really do feel it should go through your GP? You wouldn't be happy with

**Lady FC Member** 35:14

....you could get a letter...

**Facilitator 1** 35:16

... that letters is one way.. or I don't know, one way or the other example are German colleagues do actually even the Canadians, they have a centre where people can go and you can have maybe a meeting like this. So there are other ways

**Lady FC Member** 35:32

Thats a good idea. Yeah.

**Lady FC member 2** 35:32

I cant see them paying out for that in this country.

**Facilitator 1** 35:37

Sorry can you say that again..

**Male FC member 2** 35:38

the problem at the moment is getting a doctors appointment. Yes.

**Facilitator 1** 35:50

That's another issue you should to think about.

**Male FC Member** 35:52

If you just put the information out there that this project has started. Papers, social media, whatever else how many of the population are going to actually see it? How many of the population are actually going to do anything about it? Whereas if you go into the doctors, the doctors got all the information that you're going through a blood test anyway, um and he says, Oh, theres this project, would you be prepared to participate in it least somebody is getting to find out about it. Yeah.

**Male FC member 2** 36:32

But that's a different problem... as [name] said is that's a different thing. You could you were talking about the results. Yeah, there you get the results of the project. We can only the people who organise it can only do so much to say right this is the project, this went ahead. This is where the information is. These are the results. Now when I.. Because you in a way when you have to do

**Male FC Member** 37:07

yeah, people have got to see that information first before I can do anything. But so

**Facilitator 1** 37:12

If I could just say so for example, with the Health Survey for England, because it's again, representative, you would get a letter through door saying, Would you like to participate or a child in your household also like to participate? Yeah, so that's how they would approach you. So an example.

**Male FC member 2** 37:34

So what we're talking about is when the project is over and you get the results

**Facilitator 1** 37:38

Yes, exactly. So again, with it, just taking that as an example, what the Health Survey for England would do is that then they would send you your results in a letter. And then you could have a discussion with somebody over the phone. But there, they're not looking at chemical exposures. It's very different, but that's just one model. They use.

**Male member 4** 38:01

David Ogilvy 60 odd years ago 70 odd years ago said, I know that most of my advertising budget is wasted. And it's about 90%. But the trouble is, I don't know which 90% is natural. It is living in a fantasy world to think that you can communicate in PHE or any other organisation with everyone on the planet. And you would need the entire spending power of the world to be able to do it. So the way people do it is they approach the problem in much more targeted ways. They choose media and distribution channels that the target audience will look at. So for example, most youngsters seem to me to spend at least three quarters of their waking lives on their phones. So it might be possible within existing laws and GDPR regs and so on. That messages could be beamed to young people about the issue in general, asking them if they would like to respond. By answering a questionnaire. It would need to be quick or trying to leech and look at a website that they could focus on to get more information if they were interested. How many people out of 100 young people would respond in the right way, somewhere between three and six, but that's somewhere between three and 6% ahead of where we are now. Then there are local opinion formers local newspapers. U3A [note - this stands for University of the Third Age, for people of retirment age] groups who cater to people of my age. There are evening classes run by local authorities. There are loads of occasions in which the information can be made public, so that the public, I mean, you could argue the first rule of advertising is awareness. It's to make people aware that the system, the protocol, the product actually exists. And then I can find out more information about it fairly easily. GP surgeries. There were about 200 leaflets in my local GP surgery. On the occasions I've been there. I've never seen anyone pick one up.

**Male FC member 2** 40:32

I'm not talking about that now. I'm talking about the GP giving it to you personally.

**Male member 4** 40:36

Yeah, my guess is, if you said to GPs, we'd like to do this as well. They'd say, Oh, you've got to be kidding, right. I've got 10 minutes.

**Facilitator 1** 40:45

Yeah, that's true. So we've only five minutes left. And then we'll move on to the next two questions

**Male Member 3** 40:50

I'm not sure if it is a good idea to give people their individual results. It produces all sorts of issues. Especially when Children are involved and parents are interpreting results for their children. You can see we're seeing this like prostate cancer and PSA readings how people can start worrying unnecessarily. So I'm not so sure that people getting their individual results is a good idea.

**Lady FC Member** 41:16

If you've done a study then you want your results

**Male member 4** 41:19

it wasn't because..

**Male Member 3** 41:20

As a I might not feel that some I would have enough knowledge to interpret that properly would I start worrying that I got so much if

**Male FC member 2** 41:32

I wasn't talking about personal results, I was talking about you getting an information leaflet about the results of the study and the results of the study be either by population or targeted, okay, not not nothing to do with individual data points.

**Facilitator 1** 41:51

Very good points. And so, generally we would give population based information, but an individual has the right to Ask for their data if they wish to. And then we would have the obligation not only to get the data, but to give an interpretation and support if you like, so that people aren't worried unless there's something that they need to do. Now, I'm really sorry, I need to, maybe we can try and come back. I apologise because I would love to go on allday.

**Facilitator 1** 42:21

Last two questions, and they're sort of coming back, if you like to the beginning of the project which started in 2017 18. And to ask you, now that we've had this bit of a discussion very quickly, do you think that the government government should prioritise a programme to monitor levels of chemicals from the environment in our bodies? In other words, a bio monitoring programme?

**Male member 4** 42:45

Yes, all the lines of one being done in Germany that you've described, so eloquently. Government is a question of priorities, isn't it? Someone has to decide, you know, what's more important than something else? Public Health, I think has a high priority for most people. Not just their own personal health, but the health of the population at large. for all sorts of reasons, I think it's a good thing that people are more aware than was the case in my parents' generation, or even my grandparents' generation, they simply weren't aware of these issues. So they all smoked, they all drank too much. They all you know, whatever. And they all died at the age of 37.

**Male member 4** 43:38

Now we know more, and because we know more, we want to know more still. So it's a question of what can be afforded, as always with any issue, who best to carry it out what questions to ask. How do we avoid generating so much data that no one has time to understand it? And then you may as well not bother doing it. But yes, I think the public actually is trying to form this question. What is it that you guys, whoever you guys are: GPS, our hospital, hospitals, governments, local authorities, school teachers, whoever you are, what is it that you're doing to improve our health as a population or as a group? And I think a lot of people would like an answer to that question.

**Male FC member 2** 44:39

Which we hope I never find out worried about my health personally. Because I've got various beliefs and I don't care about am I going to be hit by a bus and that because I believe I'm gonna live till I'm 77 and then 'snuff it' [colloquialism for 'to die']. So I don't care about between now and 77 because I'm because I'm still going to get there. What I care about more with, with these sort of things more than anything is future generations.

**Male Member 3** 45:11

I presume you look at the traffic before you cross the road.

**Male FC member 2** 45:14

Obviously, you've got to be you've got to be sensible. I don't care about I don't think I worry about will it get cancer? Or will I get this? Or will it get that? I just, I just don't worry about it.

**Facilitator 1** 45:27

But then but then you say from a futuristic perspective

**Male FC member 2** 45:30

you know, I'm thinking, I'm thinking about, you know, I think by me, so know me, you know, your grandson. And I think about the generations after that. And I think to myself: I don't want them to be worrying about things like this in 50 or 60 years time, I want to know that something has been done in the meantime.

**Facilitator 1** 45:58

So that leads us to the Second question on that second blue group is that you've heard from us, you know, again, personally, do you think the results would affect you on your day to day life? Would you make different decisions? What kinds of things may these results influence?.

**Lady FC member 2** 46:18

Yeah! You would be more aware, more aware of what I am doing.

**Male member 4** 46:28

Smoking is probably a good example. In my parents and before them, generations, everyone smoked men, but a lot of women too, I think. Hm, and no one came in for well, this is going to kill me. And so they never stopped. And then my father's generation, they stopped a bit. My generation we stopped. My children's generation never started.

**Facilitator 1** 46:57

That's it. You think that's because of knowledge.

**Male member 4** 47:00

Because of more public awareness and the danger being quick

**Male Member 3** 47:06

Sorry, you should go to Clacton [small sea-side town in England]. Yeah yeah yeah.

**Male member 4** 47:13

You know, that says that stuff is bad for you. It's then up to each individual as to whether they say, okay, but I'm going to carry on

**Lady FC Member** 47:22

legislation about not being able to smoking inside that's hugely helped, hasn't it?

**Male FC member 2** 47:25

Yeah, its become socially unacceptable.

**Male member 4** 47:30

Sometimes I think sorry ....

**Male FC member 2** 47:34

Now you watch an old television programme, like ...I'm sorry 20 years ago 30 years ago, that you see people smoking, you see people on an old Parkinson [Popular TV Chat Show from 1971 to 1982] or something like that, they get out their cigarette. You don't see people smoking on TV now. In actual ctual fact that time is actually unrealistic. Because people do still smoke is much more.

**Lady FC Member** 48:07

They started to vape now though.

**Male FC member 2** 48:10

At the NSA

**Male member 4** 48:16

last night on the television news, I'm sure most of us saw this. The parents of that child who died on an aircraft two years ago, and they were Lebanese French that family. ...... So she the little girl I think she was like 15 or something. She bought a sandwich from pret at the airport ate it nad had a seizure on the aircraft and was dead before the pilot could land in Spain or somewhere. And now the parents have been campaigning and they've got something called Natalie's law, I think that was the girl's name, in front of Parliament. And my first reaction was I'm terribly sorry for the family who wouldn't be. But how many people in the future will be saved by this? And the answer is I don't know. And I don't know if anyone else knows either. But if the answer is four or five, then it's not a priority. If it's four or 500 or 40,000, that could be saved by not eating these chemicals in foods. Absolutely. It's got to be a priority.

**Lady FC member 2** 49:41

So it's the proportion

**Male FC member 2** 49:44

I don't have any effect on me. I don't think I think it will have any effect on me because I'm not capable. influencing people enough. That's why we're here. Because the people who can do the influence are there. And unless we tell them what we're worried about, nothing is going to happen.

**Male member 4** 50:17

Thats not how networks work and ..

**Male Member 3** 50:20

Thats how manufacturers work

**Male member 4** 50:21

Networks work by, is you have one Jesus Christ, who manages to persuade 12 other people, and over millennia, they persuade. However many million it is to believe it. One at a time.

**Male FC member 2** 50:37

And how long is that going to take her?

**Male member 4** 50:39

Very long? But yeah,

**Lady FC member 2** 50:42

But you have to start it

**Male FC member 2** 50:44

But we don't want it to take a long time because we're all worried about it. Otherwise, we wouldn't be here.

**Male member 4** 50:49

Im not saying its the only means of communication and evangelization. It's one means that the government should not overlook

**Facilitator 1** 50:58

Well, if you take David Attenborough programme for example, [Name], you haven't had a chance to speak.

**Lady FC member 2** 51:04

I love listening to everybody and I think they've put my point across. Okay.

**Facilitator 1** 51:12

So we really are, I'm really sorry that we're running out of time, because I think it's been extremely useful to hear your views. And just to let you know that, obviously we've taped these things, we will then take more detailed notes, we'll look at what you've written down. And this will then go back to me. So we're doing these focus groups not only just here, but my colleagues in Ireland and Portugal and next year, two other countries will do the same. And we'll bring the information together to have not only a UK view us, but also more European view. What are we all concerned about? What should be done on a European level? Have I forgotten anything?

**Facilitator 2** 51:56

No, thank you so much. Everything is just the feedback forms. They need to feel complete. Okay. Yes. Do

**Male Member 3** 52:03

Do you sort of look around and say, Oh, I wonder how representative of the whole population this group is. Maybe? Yeah. How do we do things? Maybe get children's views? Yes.

**Facilitator 1** 52:15

I iwll let [Fascilitator 3] answer that in a minute. But also for us. I think the people who volunteer are the ones who are interested. How do we get to the people who aren't interested? That is a real problem.

**Facilitator 3** 52:31

Not sure I can answer that really. Yeah. if you know, and as [name] knows as well as you're on a panel. It's only the people that you know, that take part in our survey in the Ipsos Mori survey that should have come to your door. That will actually that we get your name addresses from. So there are a certain type of people that might take that survey or be at home in first place to do it as well. Retired people, people that are in working for themselves and we are aware that, you know, know, we ar aware that they are not represetaive of the entire home. We try as much as we can to go out to voluntary organisations and charities to find out the people as well.

**Facilitator 1** 53:34

It's difficult, it is not an easy task.

**Facilitator 3** 53:38

We've sneaked in a chemical questionnaire actually in our general feedback form. So just really quickly, don't put too much on it. Just fill that in for us.

**Facilitator 1** 53:54

But I'd really like to thank you. It's been a very good discussion its been excellent.

**Facilitator 1** 54:01

When you have to stop people discussing things you realise its working well.

**Facilitator 1** 54:35

okay for Halloween you can buy fake blood [props used to illustrate samples]

**Male Member 3** 54:44

But how do you know it's fake?

**Male FC Member** 54:52

Because it say so on the label [laughter].

**2 Integrated code list**

[Below you can find the coding scheme that was used for the Austrian focus group discussion (text segments were attributed to these codes; text segments with the same codes were compared). As mentioned above, even if general themes of the discussion are fixed by the guideline, the participants have room in the discussion to bring up new related topics, which then also have to be reflected in the coding scheme. Considering the very general questions asked in this focus group and the very broad target group, there might be great variations between the discussion regarding the topics the participants bring up and the emphasis they place on them. Therefore, this guideline should only be used as a starting point and can be adapted during the analysis of the material. The first level of the codes was almost never used (only if aspects of the topic were mentioned that weren’t covered by the subcodes, were mentioned only once or were very general in nature).]

Personal access to and interest in topic; prior knowledge

Concerns reg. exposure to harmful substances

Concrete pollutants

Areas (of consumption)

Responsibility of various actors to prevent pollutant diffusion

Science

Politics

Economy

Consumers

To create political pressure

To inform oneself

To change consumption behaviour

Limits of consumer responsibility

Attitudes regarding biomonitoring

Concerns regarding biomonitoring

Effectiveness

Data protection

Expectations of biomonitoring

Creating new regulations

Dissemination of results

Handling of Data

Disseminating information about harmful substances to consumers

Information deficits of participants

(Evaluation of) different ways of information dissemination

Barriers to the implementation and evaluation of information by consumers

Obstacles in the fight against the spread of pollutants

in society as a whole

in/for science

in/for the economy

for consumers

in/for politics

Ideas for policy measures against the spread of pollutants

Diffusion of knowledge

Substitutes for harmful substances
