# Supplementary figures and images for: Chemical Exposure: European Citizens’ Perspectives, Trust, and Concerns on Human Biomonitoring Initiatives, Information Needs, and Scientific Results
Source: Int J Environ Res Public Health. 2021 Feb 5;18(4):1532. doi: 10.3390/ijerph18041532 (PMC7914422; doi:10.3390/ijerph18041532)

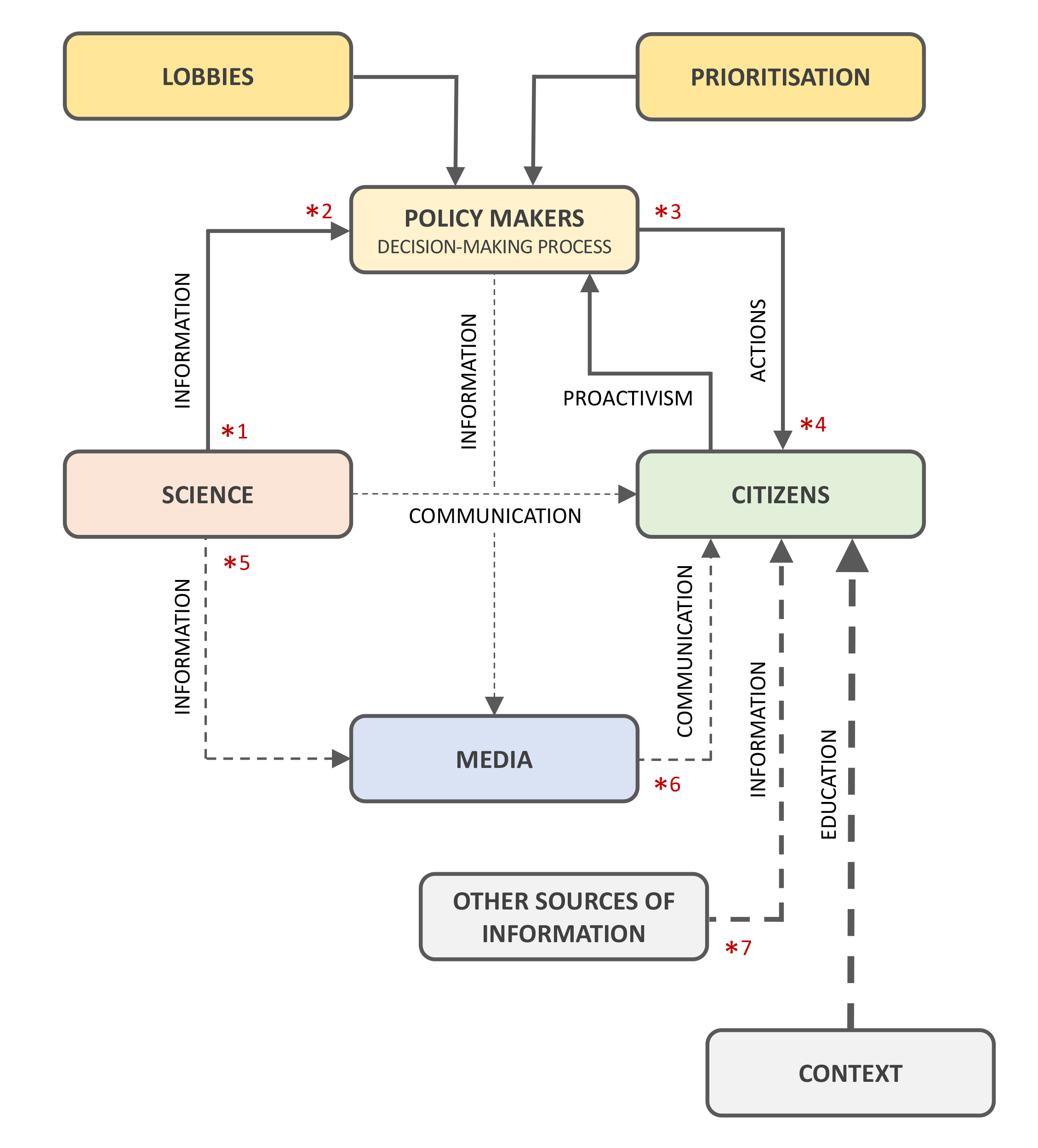

Supplement: Supplementary file 1 [file ijerph-18-01532-s001.zip › Modelo_HBM_FG.jpg]
